# MiR-320a as a Potential Novel Circulating Biomarker of Arrhythmogenic CardioMyopathy

**DOI:** 10.1038/s41598-017-05001-z

**Published:** 2017-07-06

**Authors:** Elena Sommariva, Yuri D’Alessandra, Floriana Maria Farina, Michela Casella, Fabio Cattaneo, Valentina Catto, Mattia Chiesa, Ilaria Stadiotti, Silvia Brambilla, Antonio Dello Russo, Corrado Carbucicchio, Giulia Vettor, Daniela Riggio, Maria Teresa Sandri, Andrea Barbuti, Gianluca Vernillo, Manuela Muratori, Matteo Dal Ferro, Gianfranco Sinagra, Silvia Moimas, Mauro Giacca, Gualtiero Ivanoe Colombo, Giulio Pompilio, Claudio Tondo

**Affiliations:** 1 Vascular Biology and Regenerative Medicine Unit, Centro Cardiologico Monzino IRCCS, Milan, Italy; 2 Immunology and Functional Genomics Unit, Centro Cardiologico Monzino IRCCS, Milan, Italy; 3 Cardiac Arrhythmia Research Center, Centro Cardiologico Monzino IRCCS, Milan, Italy; 4 Laboratory Medicine Unit, Centro Cardiologico Monzino IRCCS, Milan, Italy; 5 Department of Biosciences, Università degli Studi di Milano, Milan, Italy; 6 Human Performance Laboratory, Faculty of Kinesiology, University of Calgary, Calgary, Canada; 7 CeRiSM, Research Center Sport, Mountain and Health, Department of Neurological and Movement Sciences, Università degli Studi di Verona, Rovereto, Italy; 8 Department of Cardiovascular Imaging, Centro Cardiologico Monzino IRCCS, Milan, Italy; 9 Cardiovascular Department, Azienda Sanitaria Universitaria Integrata di Trieste, Trieste, Italy; 10 Molecular Medicine Laboratory, International Centre for Genetic Engineering and Biotechnology, Trieste, Italy; 11 Department of Medical, Surgical and Health Sciences, Azienda Sanitaria Universitaria Integrata di Trieste, Trieste, Italy; 12 Department of Clinical Sciences and Community Health, Università degli Studi di Milano, Milan, Italy

## Abstract

Diagnosis of Arrhythmogenic CardioMyopathy (ACM) is challenging and often late after disease onset. No circulating biomarkers are available to date. Given their involvement in several cardiovascular diseases, plasma microRNAs warranted investigation as potential non-invasive diagnostic tools in ACM. We sought to identify circulating microRNAs differentially expressed in ACM with respect to Healthy Controls (HC) and Idiopathic Ventricular Tachycardia patients (IVT), often in differential diagnosis. ACM and HC subjects were screened for plasmatic expression of 377 microRNAs and validation was performed in 36 ACM, 53 HC, 21 IVT. Variable importance in data partition was estimated through Random Forest analysis and accuracy by Receiver Operating Curves. Plasmatic miR-320a showed 0.53 ± 0.04 fold expression difference in ACM vs. HC (p < 0.01). A similar trend was observed when comparing ACM (n = 13) and HC (n = 17) with athletic lifestyle, a ACM precipitating factor. Importantly, ACM patients miR-320a showed 0.78 ± 0.05 fold expression change vs. IVT (p = 0.03). When compared to non-invasive ACM diagnostic parameters, miR-320a ranked highly in discriminating ACM vs. IVT and it increased their accuracy. Finally, miR-320a expression did not correlate with ACM severity. Our data suggest that miR-320a may be considered a novel potential biomarker of ACM, specifically useful in ACM vs. IVT differentiation.

## Introduction

Arrhythmogenic CardioMyopathy (ACM) is a genetic disease characterized by a progressive fibro-fatty substitution of the ventricular myocardium, particularly pronounced in the right ventricle (RV), associated with life-threatening ventricular arrhythmias and heart failure (HF). With an estimated prevalence ranging from 1:2000 to 1:5000, ACM affects men more frequently than women^1^, representing one of the most diffuse cause of sudden death in young athletes^2^. Up to 50% of individuals with ACM harbour an autosomal dominant mutation in one of the genes coding for cardiac desmosome components^3^. Desmosomes are complex cellular junctions with structural and intracellular signalling functions, mediated by the WNT/β-catenin pathway^4^, which is a key regulator of cardiac development, response to injury^5^, and cell fate^6^.

**Figure 1:**
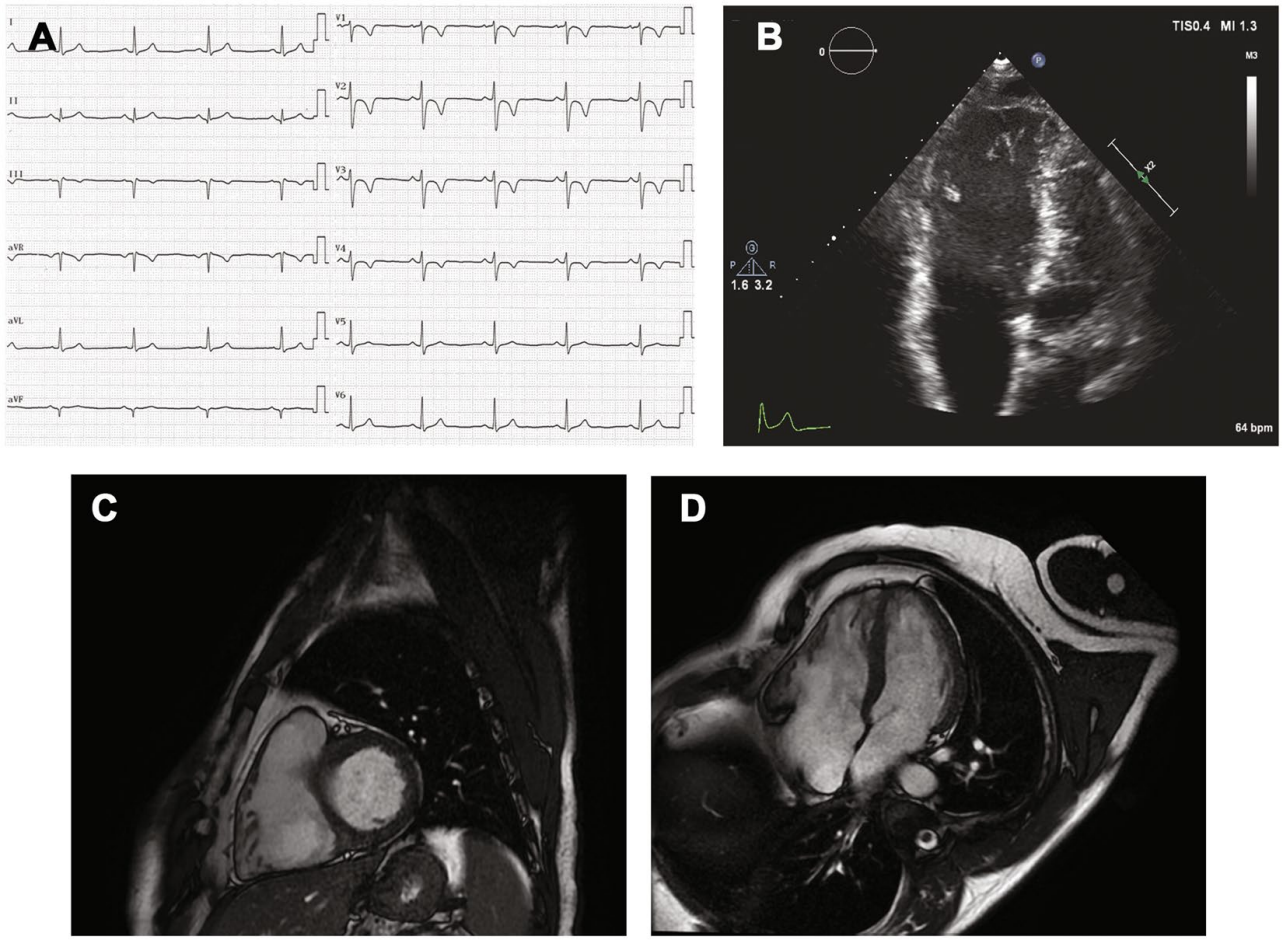
Non-invasive diagnostic tests in an Arrhythmogenic CardioMyopathy (ACM) patient. (**A**) Baseline 12-lead ECG shows regular sinus rhythm with inverted T waves in the precordial leads from V1 to V4. (**B**) Four-chamber echocardiographic imaging shows enlarged right ventricular diastolic volumes. Cardiac magnetic resonance: four-chamber short-axis (**C**) and long axis (**D**) images reveal dilated right ventricle with segmental kinesis abnormalities, mainly involving the inferolateral wall.

The diagnosis of ACM is challenging and often late after disease onset because of the broad spectrum of phenotypic presentations. Currently, no single gold standard diagnostic method is available. Even the identification of an associated genetic defect, when possible, may be not conclusive because of the high variability in penetrance and expressivity. According to the International Task Force recommendations, the decision-making process relies on a scoring system of major and minor criteria, including clinical, instrumental and genetic tests. ACM is ascertained in the presence of either two major, one major plus two minor, or four minor criteria^7^. Importantly, using non-invasive tools only (e.g. Fig. 1), a conclusive diagnosis is not reached in a substantial number of patients. Invasive procedures such as endomyocardial biopsy and/or electroanatomic mapping may be needed to confirm the presence of the disease^8, 9^.

Particularly challenging is also the differential diagnosis for ACM, which includes Idiopathic Ventricular Tachycardia (IVT), a primary electrical cardiac condition with a similar arrhythmic phenotype. The two entities share several phenotypic similarities: left bundle branch block with inferior axis ventricular tachycardia, symptoms aggravation after exercise, predominant occurrence in young, otherwise healthy individuals. Nonetheless, while ACM is a life-threatening disease, IVT has a more favourable outcome and different therapeutic options^10^. Thus, early and definite distinction between ACM and IVT is of the upmost clinical importance. As a consequence, novel ACM early diagnostic biomarkers are currently under investigation. The plakoglobin mislocalization assay is a promising candidate as a new diagnostic tool^11^, however it is exploitable only in bioptic or autoptic samples only. Plasma BIN1 has recently been proposed as predictor of arrhythmias in ACM, although restricted to subjects presenting symptomatic HF^12^.

In the last few years microRNAs (miRNAs), short (22–24 nucleotides) non-coding RNAs, have been found to be stably expressed in the systemic circulation of both animals and humans^13^. Notably, several reports from others and our laboratory described the diagnostic potential of circulating miRNAs in different heart conditions, including myocardial infarction^14^, HF^15^ and atrial fibrillation^16^.

No previous study has evaluated circulating miRNAs as potential biomarkers of ACM. In the present work, we assessed miRNAs expression in plasma samples obtained from ACM and NON-ACM subjects. Our results indicate that miR-320a is significantly less expressed in ACM patients’ plasma and that it may be helpful in differentiating ACM from IVT.

## Methods

### Ethical statement

This study complies with the Declaration of Helsinki and was approved by the Ethics Committees of Centro Cardiologico Monzino (06/06/2012), Centro Cardiovascolare dell’Azienda Ospedaliera Universitaria di Trieste (Project Number: N.O.43/2009, prot.2161) and Dipartimento di Neuroscienze, Biomedicina e Movimento, Università degli Studi di Verona (March, 30/03/2012, #99). Written informed consent was obtained from all participants.

### Study population

A total of 110 male subjects were enrolled in three independent recruiting centres: 53 healthy controls (HC), 36 patients affected by ACM and 21 patients affected by IVT. Baseline characteristics of the different cohorts of patients are reported in Tables 1 and 2 and Supplementary Tables S1, S2 and S3.

**Table 1:** Baseline characteristics of enrolled subjects.

	HC	IVT	ACM	p HC vs. ACM	p IVT vs. ACM
N	53	21	36	—	—
Age (years)	42.88 ± 1.57	48.05 ± 3.09	48.39 ± 2.22	0.07	0.96
Male gender n (%)	53 (100)	21 (100)	36 (100)	—	—
BMI	24.36 ± 0.35	24.40 ± 0.43	24.39 ± 0.43	0.95	0.26

HC, Healthy Control subjects; IVT, patients affected by Idiopathic Ventricular Tachycardia; ACM, patients affected by Arrhythmogenic CardioMyopathy; BMI, Body Mass Index.

**Table 2:** Established Arrhythmogenic CardioMyopathy (ACM) major or minor diagnostic criteria^7^ and clinical characteristics in Idiopathic Ventricular Tachycardia (IVT) vs. ACM patients.

		IVT	ACM	p IVT vs. ACM
Global or regional dysfunction and structural alteration n (%)	Minor	2 (9.52)	12 (33.33)	0.04
	Major	0 (0)	18 (50)	<0.01
Tissue characterization n (%)	Minor	—	2 (8.69)	—
	Major	—	12 (52.17)	—
Repolarization abnormalities n (%)	Minor	4 (0.19)	9 (0.25)	0.61
	Major	1 (0.05)	17 (0.47)	<0.01
Depolarization abnormalities n (%)	Minor	0 (0)	8 (22.22)	0.02
	Major	0 (0)	10 (27.78)	<0.01
Arrhythmias n (%)	Minor	21 (100)	15 (41.67)	<0.01
	Major	0 (0)	17 (47.22)	<0.01
Family history n (%)	Minor	0 (0)	4 (11.11)	0.11
	Major	0 (0)	8 (22.22)	0.02
LVEF (%)		58.85 ± 1.29	58.55 ± 1.75	0.91
TAPSE (mm)		24.72 ± 0.93	19.94 ± 0.83	<0.01
MAE n (%)		2 (9.52)	24 (66.67)	<0.01
BNP (pg/ml)		21.88 ± 3.11	46.85 ± 8.34	0.15
miR-320a (relative expression)		1 ± 0.10	0.78 ± 0.05	0.03

LVEF, Left Ventricular Ejection Fraction (n = 21 IVT, n = 35 ACM); TAPSE, Tricuspid Annular Plane Systolic Excursion (n = 18 IVT, n = 35 ACM); MAE, Prior Major Arrhythmic Events (n = 21 IVT, n = 36 ACM); BNP, Brain Natriuretic Peptide (n = 20 IVT, n = 32 ACM).

HC without previous history of heart disease, age- and sex-matched with ACM patients have been selected. Healthy long-distance runners, classified in group D of cardiovascular risk according to the COCIS score^17^, were classified as HC athletes. Blood samples were withdrawn in a resting period.

ACM diagnosis was reached according to the International Task Force criteria^7^. Among ACM patients, 13 subjects were classified in group D of cardiovascular risk^17^ and defined as ACM-athletes for comparison with HC athletes.

IVT patients were characterized by right or left ventricular outflow tract tachycardia in the absence of any sign of structural heart disease^18^ as documented by 2D-echocardiography, magnetic resonance and/or electroanatomic mapping^9^.

### BNP assay

Brain Natriuretic Peptide plasma levels were analysed using the BNP ARCHITECT chemiluminescent microparticle immunoassay on the ARCHITECT i2000 System (Abbott Diagnostics), following the manufacturer’s instructions.

### Electroanatomic Mapping

Electroanatomic mapping of the RV was performed using CARTO system (Biosense Webster). At least 150 mapping points were sampled with an irrigated-tip Navi-Star catheter (Biosense Webster). Bipolar and unipolar electrograms were analysed. Reference values for identifying normal endocardial bipolar and unipolar signals were defined as >1.5 and >5.5 mV, respectively.

### 2D-Echocardiography

2D-echocardiography studies were performed with an iE33 system (Philips Medical Systems) using the S5-1 sector array probe. Complete standard 2D-echocardiography was performed according to clinical practice. In particular, biplane left ventricular ejection fraction (LVEF) was measured from apical 4-chamber and 2-chamber views by the area-length method. From the 4-chamber apical view, the M-mode cursor was positioned at the junction of the tricuspid valve plane with the RV free wall to evaluate tricuspid annular plane systolic excursion (TAPSE), defined as the difference in the displacement of the RV base from end-diastole to end-systole and used as an index of RV function^19^.

### Magnetic Resonance

Patients were evaluated in a 1.5-T scanner (Discovery MR450; GE Healthcare). CMR data sets were analysed with a dedicated software (Report Card 4.0; GE Healthcare) as previously described^20^.

### Plasma and RNA preparation

Blood samples (5 ml) were collected in EDTA coated tubes and centrifuged at 1500 × g for 15 min. Supernatants were collected, centrifuged again at 16000 × g for 15 min to obtain cells- and platelet-free plasma, and stored at −80 °C as 400 µl aliquots until usage.

Total RNA was extracted from plasma as previously described^21^. Briefly, 1 ml of TRIzol reagent (Life Technologies) was used for each 400 µl of plasma followed by phase separation and RNA precipitation with 1 ml of isopropanol after addition of 30 μg of glycogen (Roche). RNA pellets were obtained by centrifugation, washed with 1 ml of 70% ethanol, and centrifuged again for 10 min at 12.000 × g. Supernatants were discarded and pellets were resuspended in RNase-free water.

### miRNA Screening

TaqMan Human microRNA Card A Arrays version 2.0 (Life Technologies) were used for expression screening of 377 miRNAs, using 3 µl of total RNA from 3 ACM and 3 HC subjects. Reverse transcription (RT) and pre-amplification steps were performed according to the manufacturer’s protocol. Data were analysed with the ExpressionSuite software v1.0.3 (Life Technologies), normalized by the Global Normalization method. All miRNAs presenting a Ct ≥ 35 were considered as not expressed. miRNAs whose expression in ACM patients consistently (t-test p < 0.05) differed from controls more than 2-folds were considered for the validation phase.

### miRNA expression analysis

The expression of each single miRNA was evaluated using TaqMan microRNA assays (Life Technologies) following manufacturer’s instructions. No RNA quantification was possible neither by spectrophotometric methods nor by using 2100 Bioanalyzer (Agilent Technologies). Therefore, the same volume of RNA (2 µl) was used in the RT step for technical consistency. As previously described^14, 22–24^ we used an internal reference, so that all subsequent calculations were independent from RNA input. We selected 3 miRNAs, basing on screening data analysis (miR-210, miR-375) and on previous reports^24^ (miR-16) for validation in all samples as potential references. Our results showed high intra-group data variability in all groups when using miR-16. Similarly, data obtained using miR-375 showed a less than optimal expression stability, as it was undetectable in a few samples. Conversely, miR-210 showed a strong expression in all samples with very limited intra-group variability when used for normalization. In addition, we confirmed, using NormFinder (http://moma.dk/normfinder-software) that miR-210 has the lowest intra group variability and the best stability value (data not shown). Thus, miR-210 was selected as reference miRNA. The 2^−ΔΔCt^ method was used to calculate miRNA expression relative to the mean expression of a calibrator group, indicated for each comparison.

### Statistical Analysis

Continuous variables are presented as mean ± SEM, when not differently indicated. Comparisons between classes were performed by either parametric (One-way ANOVA, t-test) or non-parametric (Kruskal-Wallis, Mann-Whitney) tests, after assessment of the normality of the data by Kolmogorov-Smirnov test. Categorical variables are reported as frequencies and compared by Fisher’s exact test. The Random Forests method^25^ was used to rank the importance of selected variables in classifying ACM vs. IVT, by constructing multiple decision trees, bootstrapping 70% of samples 100 times. The mean decrease in Gini index was used as measure of the variable importance: Gini index measures the divergences between the probability distributions of the variable values. Therefore, variables with higher mean decrease in Gini index are more important for classification of the samples. The area under the curve (AUC) of Receiver Operating Characteristic (ROC) plots was used to assess diagnostic accuracy of selected variables: each plot shows the mean curve of 100 reiterated analyses through different bootstrap sampling of training and test sets. Correlation of miR-320a expression with clinical variables was calculated and reported as Spearman rho (ρ).

A two tailed p-value ≤ 0.05 was considered significant. Statistical analysis was performed using GraphPad Prism5 (GraphPad Software), and randomForest (v. 4.6–12) and ROCR (v.1.0–7) packages in R (v 3.3.0) environment.

### Data Availability

The datasets generated and analysed during the current study are available from the corresponding authors on request. In particular, the raw data generated by the screening can be analysed using the dedicated free online tool (http://www.thermofisher.com/it/en/home/cloud.html).

## Results

### miR-320a expression in Arrhythmogenic CardioMyopathy patients and Healthy Controls

**Figure 2:**
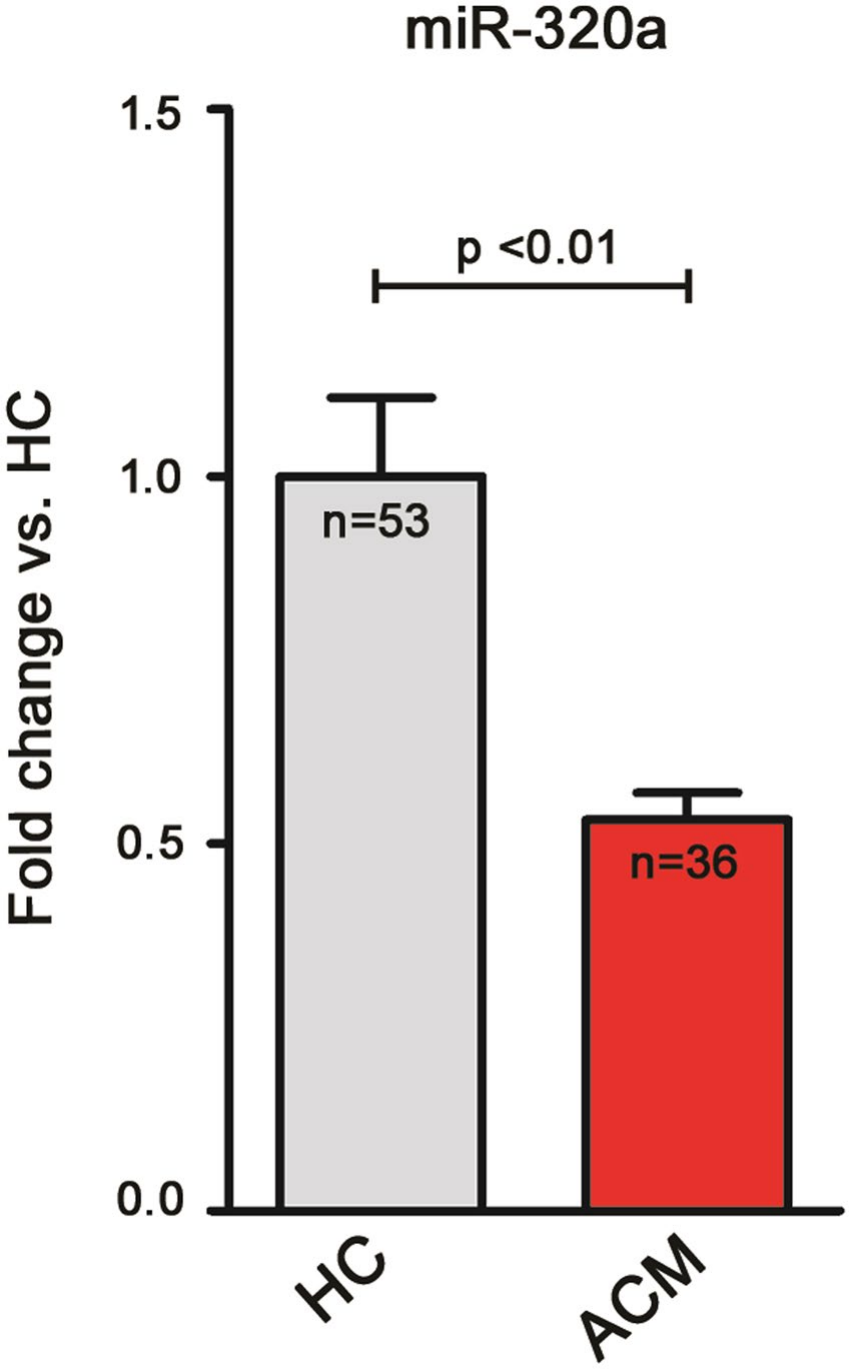
miR-320a shows a lower plasma expression in Arrhythmogenic CardioMyopathy (ACM) patients when compared to Healthy Controls (HC). Relative expression of plasma miR-320a in ACM patients (n = 36) with respect to HC (n = 53); the mean expression value in HC was arbitrarily set to one (HC 1.00 ± 0.11; ACM 0.53 ± 0.04; p < 0.01, t-test).

We conducted a screening of expression of 377 miRNAs in plasma samples from 3 symptomatic ACM patients and 3 age- and sex-matched healthy donors. 121 miRNAs were detectable in all screened plasma samples and 5 miRNAs showed a potential differential expression (see Supplementary Table S4). We then tested their expression by RT-qPCR in the whole cohort of ACM patients and HC (n = 36 and n = 53, respectively; baseline characteristics of the study groups are reported in Table 1). Only miR-320a showed a statistically significant lower expression in ACM patients with respect to HC (0.53 ± 0.04 and 1.00 ± 0.11, respectively, p < 0.01; Fig. 2).

### Assessment of athletic status influence on miR-320a expression

**Figure 3:**
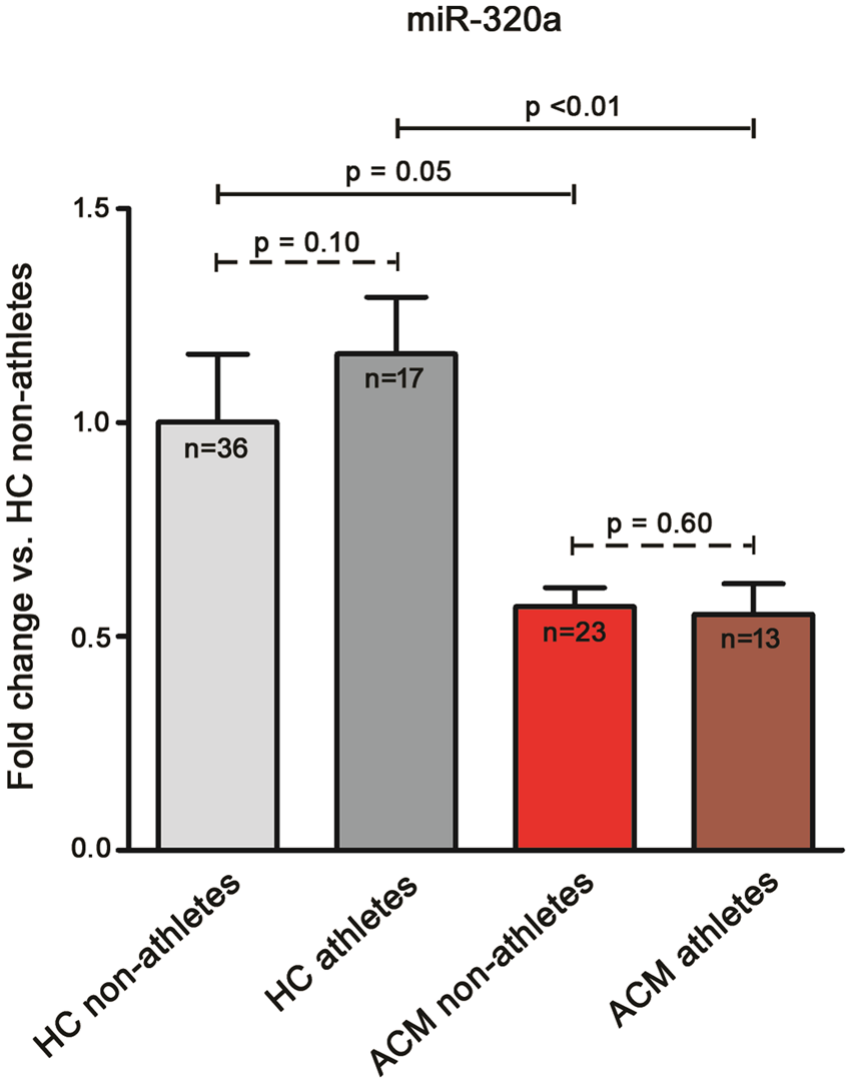
miR-320a plasmatic expression is not influenced by athletic lifestyle. Plasmatic relative expression of miR-320a in non-athlete Healthy Controls (HC; n = 36), athlete HC (n = 17), non-athlete Arrhythmogenic CardioMyopathy patients (ACM; n = 23), and athlete ACM patients (n = 13). Results are shown with respect to the mean expression of miR-320a in non-athlete HC, arbitrarily set to one. Kruskal-Wallis followed by Dunns test was used to determine that no significant difference is observed between non-athlete and athlete HC subgroups (1.00 ± 0.22 vs. 1.16 ± 0.13; p = 0.10) and between non-athlete and athlete ACM subgroups (0.57 ± 0.04 vs. 0.55 ± 0.07; p = 0.60). HC vs. ACM differences are confirmed both between the non-athlete (1.00 ± 0.22 vs. 0.57 ± 0.04; p = 0.05) and athlete (1.16 ± 0.13 vs. 0.55 ± 0.07; p < 0.01) subgroups.

Since 13 out of 36 ACM patients in our cohort were athletes (36.11%), and strenuous physical activity is considered a precipitating factor for the development of the disease^26^, we compared their miR-320a plasmatic expression with the sub-populations of HC athletes (n = 17; 32.07%), and these two subgroups with the non-athletic counterparts (the baseline characteristics of the four subgroups are reported in Tables S1 and S2). As depicted in Fig. 3, no difference was observed between non-athlete and athlete subgroups both in HC (1.00 ± 0.22 vs. 1.16 ± 0.13, respectively; p = 0.10) and ACM patients (0.57 ± 0.04 vs. 0.55 ± 0.07, respectively; p = 0.60). On the contrary, the lower plasmatic expression of miR-320a in ACM vs. HC was confirmed both in non-athlete (p = 0.05) and athlete (p < 0.01) subgroups.

### miR-320a expression in Arrhythmogenic CardioMyopathy and Idiopathic Ventricular Tachycardia patients

**Figure 4:**
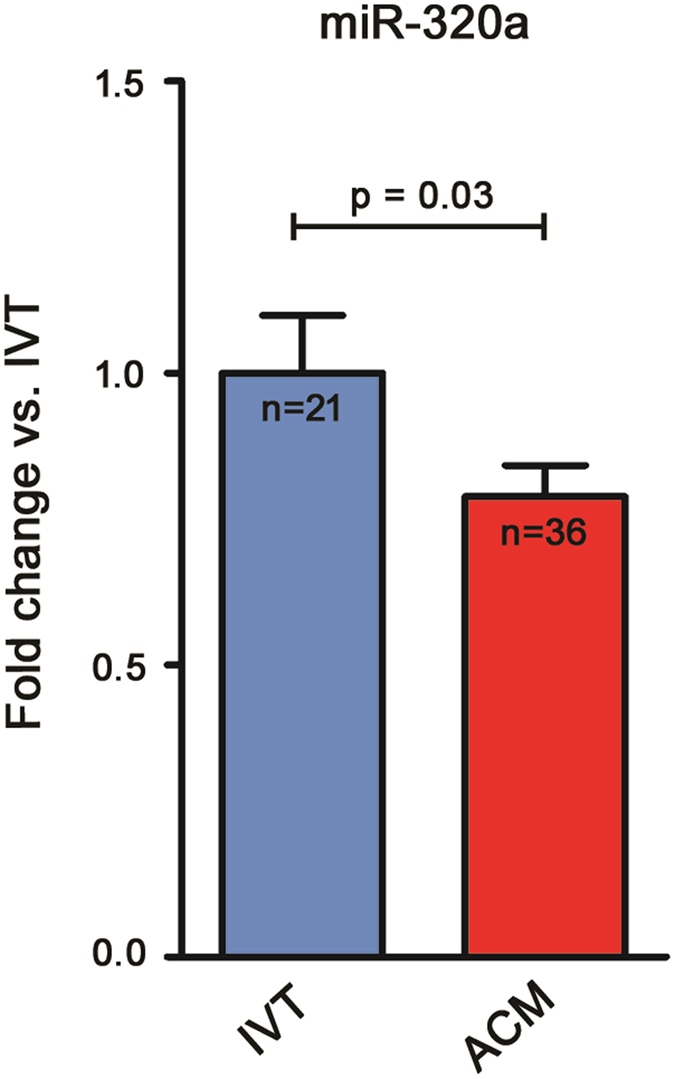
miR-320a shows a lower plasmatic expression in Arrhythmogenic CardioMyopathy (ACM) patients when compared to patients affected by Idiopathic Ventricular Tachycardia (IVT). Plasma relative expression of miR-320a, in ACM patients (n = 36), with respect to mean expression in IVT patients (n = 21), arbitrarily set to one. (IVT 1 ± 0.10; ACM 0.78 ± 0.05; p = 0.03, Mann-Whitney).

To ascertain its potential benefit in differential diagnosis, plasma miR-320a expression was compared in ACM (n = 36) vs. IVT (n = 21) patients. As presented in Fig. 4, ACM patients showed significantly lower plasma levels of miR-320a than IVT patients (0.79 ± 0.05 fold; p = 0.03). Baseline characteristics of patient groups are described in Table 1, clinical and pathological features are reported in Table 2 and Table S3.

### Ranking of non-invasive variables discriminating between Arrhythmogenic CardioMyopathy and Idiopathic Ventricular Tachycardia

**Figure 5:**
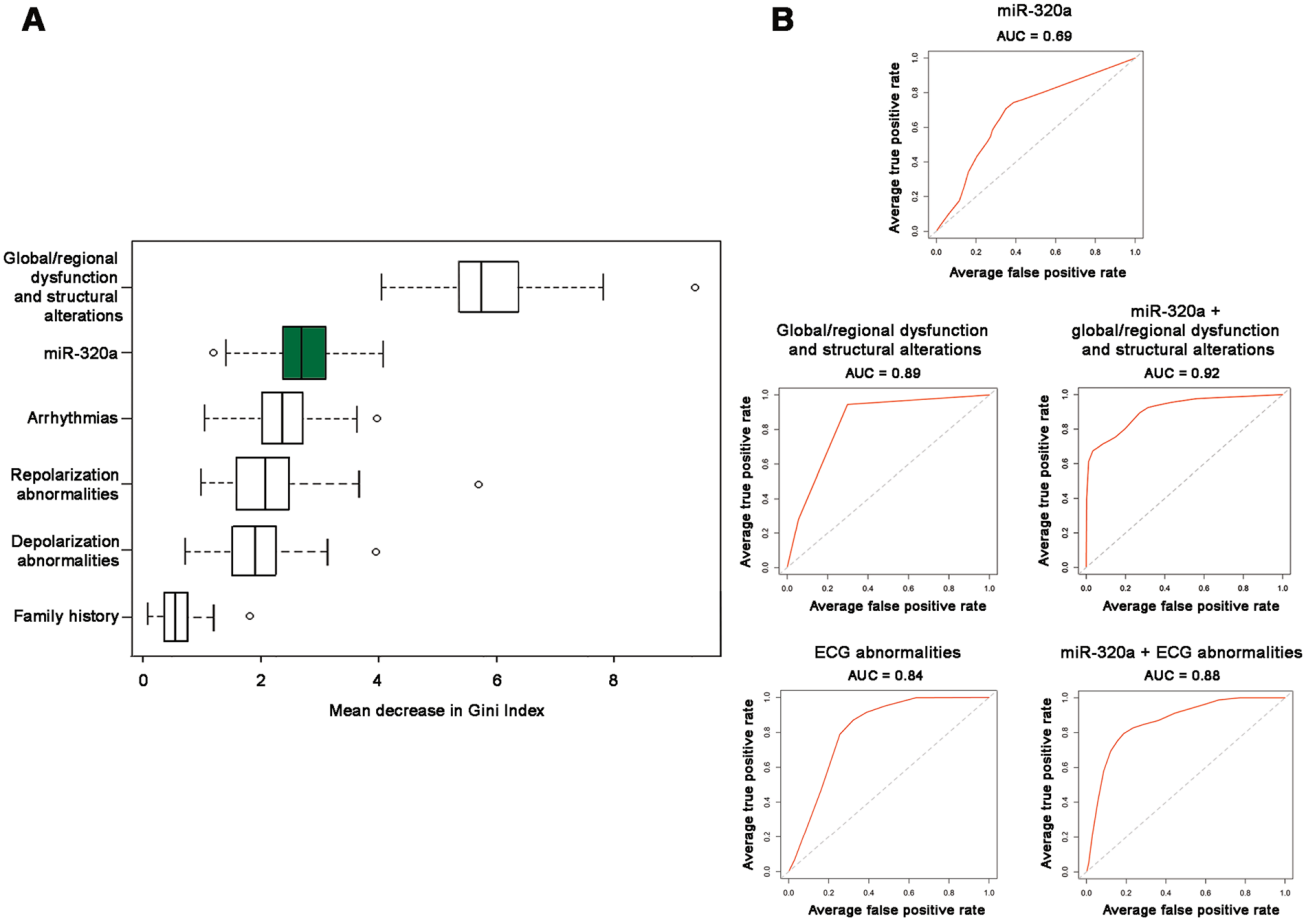
Importance of non-invasive ACM established diagnostic variables and miR-320a in Arrhythmogenic CardioMyopathy (ACM) vs. Idiopathic Ventricular Tachycardia (IVT) classification. (**A**) Boxplots show the distribution of mean decreases in Gini index for non-invasive ACM diagnostic criteria and miR-320a (green boxplot), ranked based on the importance in data partition as calculated by the Random Forests procedure. Data are shown as median[Q1–Q3]: global/regional dysfunction and structural alteration, 5.75[5.40–6.39]; miR-320a, 2.72[2.43–3.12]; arrhythmias, 2.45[2.10–2.79]; repolarization abnormalities, 2.17[1.68–2.59]; depolarization abnormalities, 2.03[1.65–2.38]; family history, 0.66[0.48–0.86]. (**B**) The Area Under the Curves (AUC) of the ROC analysis was used to evaluate diagnostic accuracy of selected variables used to classify ACM vs. IVT patients and the added value by circulating miR-320a expression. ECG abnormalities: depolarization and repolarization abnormality criteria.

To evaluate the performance of miR-320a in partitioning ACM vs. IVT patients, we used Random Forest learning method, considering substantial non-invasive variables that can be used to distinguish the two populations (Table 2). As shown in Fig. 5A, compared to each non-invasive parameter currently used to identify ACM patients (i.e. diagnostic criteria^7^), miR-320a showed a mean decrease in Gini Index value of 2.72[2.43–3.12] (median[Q1–Q3]), ranking second only to global/regional dysfunction and structural alteration 5.76[5.40–6.39], suggesting a higher classification potential than other well-known clinical criteria, in our cohort. ROC curve analyses (Fig. 5B) showed a moderate discriminating capacity of miR-320a alone (AUC = 0.69) and a slight increase in ACM prediction when miR-320a was added to global/regional dysfunction criterion (AUC = 0.89 and 0.92, respectively) or to ECG abnormalities (AUC = 0.84 and 0.88, respectively).

Similarly, as shown in Fig. S1, miR-320a presented a mean decrease in Gini Index of 3.57[3.09–4.01], higher than other non-invasive parameters that may help clinical distinction between the two diseases, namely TAPSE and LVEF^27^, Major Arrhythmic Events (MAE, defined as prior sustained ventricular tachycardia or ventricular fibrillation or aborted sudden cardiac death)^18^, and elevated BNP^28^. Indeed, miR-320a slightly increased the ACM vs. IVT discrimination potentials of the four parameters (Fig. S2).

### Assessment of miR-320a association with Arrhythmogenic CardioMyopathy severity

We then evaluated whether miR-320a expression was associated with ACM severity. No correlation was found with LVEF and TAPSE (Table 3). Similarly, no correlation was found with levels of circulating BNP, a marker of HF previously associated with RV dysfunction in ACM^29^. RV low voltage areas at electroanatomic mapping identifies fibro-fatty substitution in ACM^30^; in particular, unipolar mapping allows detection of epicardial pathological involvement^31^. miR-320a plasmatic expression was not associated with low potentials areas of the RV measured neither by bipolar nor unipolar mapping (Table 3). In addition, no difference in miR-320a plasmatic expression was observed when comparing patients with or without history of MAE, as reported in Table 4. Finally, ACM patients were arbitrarily categorized as affected by either severe or mild disease based on the presence or absence (at imaging) of ventricular dilation and bulging: again, miR-320a did not show any difference in expression between the two groups (Table 4).

**Table 3:** miR-320a expression does not correlate with continuous variables associated to Arrhythmogenic CardioMyopathy (ACM) severity.

Variables	n	ρ	p
LVEF	35	−0.25	0.16
TAPSE	35	−0.32	0.07
BNP	32	−0.20	0.29
Bipolar scar area	17	0.07	0.78
Unipolar scar area	17	−0.05	0.84

LVEF, Left Ventricular Ejection Fraction; TAPSE, Tricuspid Annular Plane Systolic Excursion; BNP, Brain Natriuretic Peptide; Bipolar Scar Area, percentage of right ventricle non-conductive (<1.5 mV) area obtained by bipolar analysis of electroanatomic mapping on right ventricle total area; Unipolar Scar Area, percentage of right ventricle non-conductive (<5.5 mV) area obtained by unipolar analysis of electroanatomic mapping on right ventricle total area. For each variable the Spearman’s rank correlation coefficient (ρ) and its statistical significance (p) are reported.

**Table 4:** miR-320a expression does not vary in subgroups defined by categorical variables associated with Arrhythmogenic CardioMyopathy (ACM) severity.

	MAE^+^	MAE^−^	p
n	24	12	
miR-320a	0.35 ± 0.03	0.42 ± 0.05	0.22
	**Severe ACM (imaging)**	**Mild ACM (imaging)**	**p**
n	11	18	
miR-320a	0.31 ± 0.04	0.37 ± 0.03	0.16

MAE^+^, patients presenting with a history of Major Arrhythmic Events; MAE^−^, patients not presenting history of MAE; miR-320a, relative expression levels showed as mean fold change ± SEM (calculated with respect to HC). Severe/Mild ACM (imaging), patients presenting severe or mild ventricular impairment, respectively (defined as absence of ventricular dilation and/or bulging at cardiac echocardiography or magnetic resonance imaging). For each variable the p-value of the Fisher’s exact test is reported.

## Discussion

The diagnostic flowchart for the assessment of ACM could decisively benefit from the discovery and exploitation of non-invasive biomarkers. Since circulating miRNAs are cheap, safe, and readily accessible, they represent good candidates to be investigated. To date, no study sought an association between circulating miRNAs and ACM. In the present work, we report, for the first time, that a plasma miRNA signature may help in ACM diagnosis. Specifically, we observed that miR-320a plasma levels are significantly lower in ACM patients compared to both HC and IVT patients. Moreover, plasma levels of miR-320a were not influenced by intense physical activity, which is known to influence ACM disease penetrance^2^, as they did not change in athletes, neither in the HC nor in the ACM groups. Thus, we may speculate that miR-320a differential expression is independent from increased mechanical stretch and training-induced adaptive heart remodelling.

The correct early distinction of a life-threatening condition such as ACM from a benign condition such as IVT remains a challenging task. The miR-320a significant differential expression and its high ranking among known ACM diagnostic criteria in distinguishing ACM from IVT suggest that this microRNA has the potential to be clinically exploited for differential diagnosis. Indeed, the classification accuracy of selected established ACM diagnostic criteria is improved by the contribution of miR-320a. In addition, miR-320a performed better in discriminating ACM vs. IVT than additional non-invasive clinical parameters such as TAPSE, MAE, LVEF, and BNP levels.

Considering that clinical ACM diagnosis is challenging particularly in the initial, concealed phase, new tools, independent from disease progression, are currently needed. Since we could not detect any correlation between miR-320a expression and indexes of ACM severity, we expect that miR-320a could be used as a marker of disease presence also at its onset. Indeed it would be interesting to test its diagnostic ability to discriminate patients with early-stage ACM from those with ventricular arrhythmias not bound to develop structural defects.

It will be of interest to address, in future studies, any mechanistic role of miR-320a in ACM pathogenesis. Intriguingly, miR-320a was found increased during adipogenic differentiation of human mesenchymal bone marrow cells^32^. Moreover, miR-320a expression regulates cardiomyocyte survival^33^ and the WNT pathway by directly targeting β-catenin^34^, which are both involved in ACM pathogenesis^4^. In a previous study conducted by screening cardiac autopsy samples, 21 miRNAs were selected from all those putatively dysregulated, and were validated as being differentially expressed between ACM and HC^23^. Although miR-320a expression was not investigated, its regulation cannot be excluded. Thus, we speculate that our findings in plasma could mirror deregulation in cellular miR-320a expression, resulting in cardiac cell apoptosis and adipogenesis.

Some study limitations have to be acknowledged. First, the low samples size used for the screening may have influenced the results in terms of number of miRNAs identified as putatively regulated. Nevertheless, the subsequent validation in a consistent number of patients (given the low prevalence of the disease) confirmed the soundness of the identified dysregulation. In addition, due to the low ACM occurrence in females, we enrolled male patients only. Thus, transferability of our results to the female ACM population should be evaluated in further studies. Moreover, as a genetic characterization was not available for most of the recruited patients, a link between miR-320a expression and a specific genetic variant could not be addressed in this study. Finally, we did not investigate the ACM-specificity of miR-320a expression versus other cardiomyopathies or channelopathies. However, these conditions are generally not in the front line for differential diagnosis.

In conclusion, this is the first study addressing the involvement of circulating miRNAs in ACM. The present results pave the way to future prospective studies aimed at defining the predictive value of miR-320a in larger ACM patients’ cohorts and represent the proof of concept that plasmatic miRNAs could be exploited as potential non-invasive biomarkers of ACM diagnosis.

## Electronic supplementary material

Supplementary data
