# Supplementary material for: MiR-320a as a Potential Novel Circulating Biomarker of Arrhythmogenic CardioMyopathy
Source: Sci Rep. 2017 Jul 6;7:4802. doi: 10.1038/s41598-017-05001-z (PMC5500514; doi:10.1038/s41598-017-05001-z)
Supplement: Supplementary file 1 — Supplementary data [file 41598_2017_5001_MOESM1_ESM.doc]

**SUPPLEMENTARY MATERIAL**

**MiR-320a as a Potential Novel Circulating Biomarker of Arrhythmogenic CardioMyopathy**

Elena Sommariva PhD^*§^, Yuri D'Alessandra PhD^*§^, Floriana Maria Farina MSc, Michela Casella MD, PhD, Fabio Cattaneo MD, Valentina Catto PhD, Mattia Chiesa PhD, Ilaria Stadiotti MSc, Silvia Brambilla MSc, Antonio Dello Russo MD, PhD, Corrado Carbucicchio MD, Giulia Vettor MD, PhD, Daniela Riggio MSc, Maria Teresa Sandri MD, Andrea Barbuti PhD, Gianluca Vernillo PhD, Manuela Muratori MD, Matteo Dal Ferro MD, Gianfranco Sinagra MD, Silvia Moimas PhD, Mauro Giacca MD, PhD, Gualtiero Ivanoe Colombo MD, PhD^#^, Giulio Pompilio MD, PhD^#^, Claudio Tondo MD^#^

^§^ These authors contributed equally to this work. ^#^ These authors contributed equally to this work.

* Corresponding authors: *Elena Sommariva and Yuri D’Alessandra*, tel: +390258002752/2852, [esommariva@ccfm.it](mailto:esommariva@ccfm.it), [ydalessa@ccfm.it](mailto:ydalessa@ccfm.it), via Parea 4, 20138 Milan, Italy.

**SUPPLEMENTARY FIGURES**

**
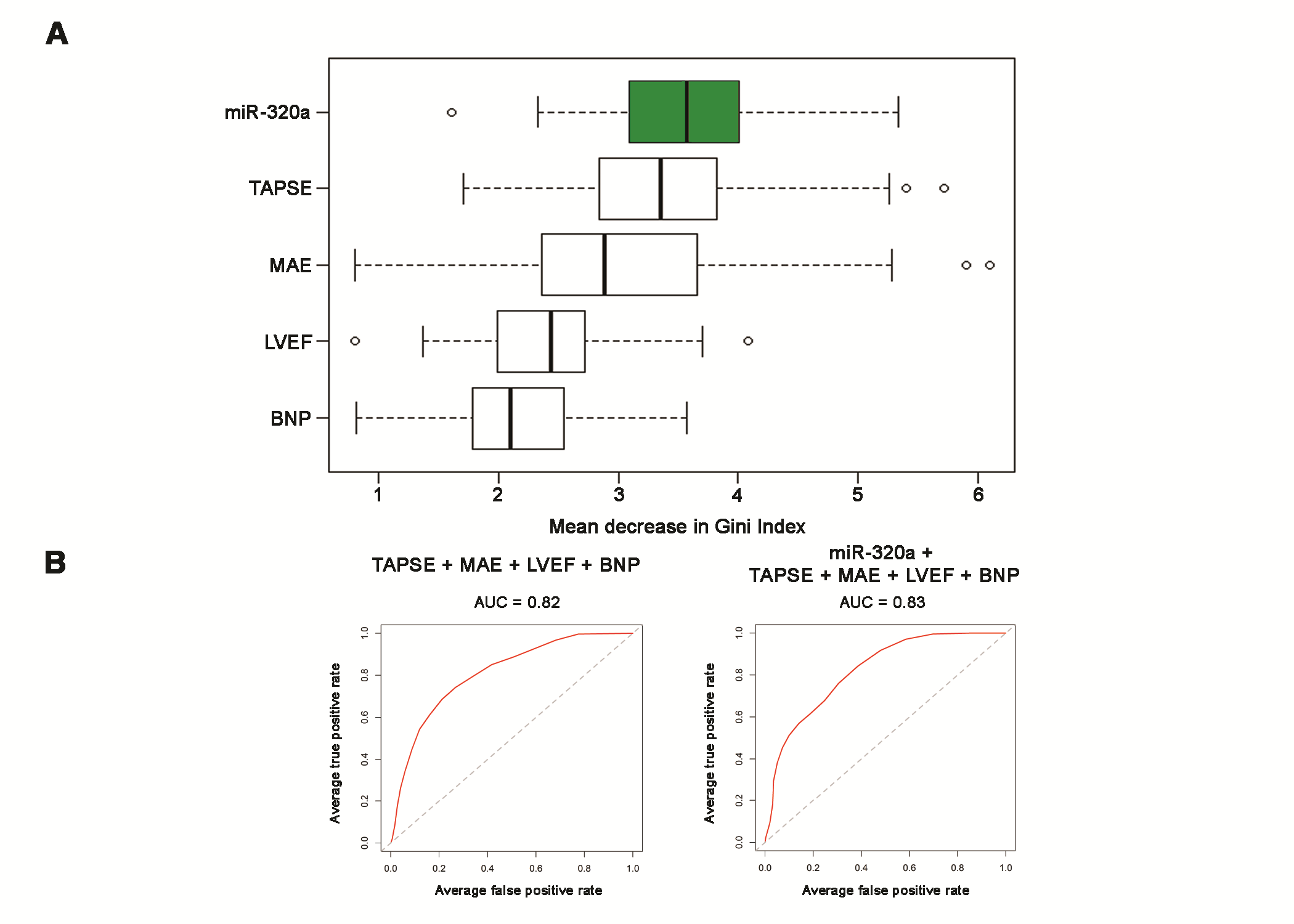
**

**FigureS1. Importance of non-invasive parameters and miR-320a in Arrhythmogenic CardioMyopathy (ACM) vs. Idiopathic Ventricular Tachycardia (IVT) classification.**

**A)** Boxplots show the distribution of mean decreases in Gini index for non-invasive parameters and miR-320a (green boxplot), ranked basing on importance in data partition as calculated by the Random Forests procedure. Data are shown as median[Q1-Q3]: miR-320a, 3.57[3.09-4.01]; Tricuspid Annular Plane Systolic Excursion (TAPSE), 3.35[2.84-3.82]; Major Arrhythmic Events (MAE), 2.88[2.36-3.64]; Left Ventricular Ejection Fraction (LVEF), 2.43[1.99-2.72]; plasmatic Brain Natriuretic Peptide (BNP), 2.10[1.79-2.54].

**B)** The Area Under the Curves (AUC) of the ROC analysis was used to evaluate diagnostic accuracy of selected variables used to classify ACM vs. IVT patients and the added value by circulating miR-320a expression.

**SUPPLEMENTARY TABLES**

**Supplementary TableS1. Characteristics of athletes and non-athletes subjects.** HC, Healthy Control subjects; ACM, patients affected by Arrhythmogenic Cardiomyopathy; BMI, Body Mass Index.

|  | **HC non-athletes** | **HC athletes** | **ACM non- athletes** | **ACM athletes** | **p-values** | | | |
| --- | --- | --- | --- | --- | --- | --- | --- | --- |
|  |  |  |  |  | **HC athletes vs. non-athletes** | **ACM athletes vs. non-athletes** | **HC vs. ACM**  **non-athletes** | **HC vs. ACM athletes** |
| **N** | 36 | 17 | 23 | 13 | - | - | - | - |
| **Age** (years) | 44.94±2.15 | 40.41±2.35 | 49.91±2.72 | 45.69±3.84 | 0.26 | 0.36 | 0.14 | 0.19 |
| **Male gender** n (%) | 36 (100) | 17 (100) | 23 (100) | 13 (100) | - | - | - | - |
| **BMI** | 25.02±0.46 | 23.14±0.38 | 24.75±0.67 | 23.51±0.49 | 0.01 | 0.20 | 0.72 | 0.57 |

**Supplementary TableS2. Medications in Arrhythmogenic CardioMyopathy (ACM) athletes and ACM non-athletes.**

|  | **ACM non-athletes** | **ACM athletes** | **p ACM athl**  **vs ACM non-athl** |
| --- | --- | --- | --- |
| **Βeta-blockers** n (%) | 20 (86.95) | 7 (53.85) | 0.05 |
| **Amiodarone** n (%) | 7 (30.43) | 1 (8.69) | 0.21 |
| **Anti-arrhythmic drugs class Ib**  n (%) | 0 (0) | 1 (8.69) | 0.36 |
| **Anti-arrhythmic drugs class Ic**  n (%) | 2 (8.69) | 1 (8.69) | 1.00 |
| **Mexiletine** n (%) | 1 (4.35) | 1 (8.69) | 1.00 |

**Supplementary TableS3. Medications in Idiopathic Ventricular Tachycardia (IVT) and Arrhythmogenic CardioMyopathy (ACM) patients.**

|  | **IVT** | **ACM** | **p** |
| --- | --- | --- | --- |
| **Βeta-blockers** n (%) | 10 (47.62) | 27 (75) | 0.05 |
| **Amiodarone** n (%) | 1 (4.76) | 8 (22.22) | 0.13 |
| **Anti-arrhythmic drugs class Ib**  n (%) | 0 (0) | 1 (2.78) | 1.00 |
| **Anti-arrhythmic drugs class Ic**  n (%) | 2 (9.52) | 3 (8.33) | 1.00 |
| **Mexiletine** n (%) | 0 (0) | 2 (5.56) | 0.53 |

**Supplementary TableS4. miRNA Screening Data.**

| **Sample name** | **miRNA** | **Cт** | **Fold Change** | **p-Value** | |
| --- | --- | --- | --- | --- | --- |
| ACM 1 | miR-223-4395406 | 17.05 | 6.352 | 0.001 |  |
| ACM 2 | miR-223-4395406 | 18.39 | 6.352 | 0.001 |  |
| ACM 3 | miR-223-4395406 | 18.34 | 6.352 | 0.001 |  |
| CTRL 1 | miR-223-4395406 | 23.34 | 1.000 | 1.000 |  |
| CTRL 2 | miR-223-4395406 | 22.95 | 1.000 | 1.000 |  |
| CTRL 3 | miR-223-4395406 | 21.23 | 1.000 | 1.000 |  |
| ACM 1 | miR-320-4395388 | 24.36 | 0.330 | 0.002 |  |
| ACM 2 | miR-320-4395388 | 24.43 | 0.330 | 0.002 |  |
| ACM 3 | miR-320-4395388 | 24.14 | 0.330 | 0.002 |  |
| CTRL 1 | miR-320-4395388 | 24.31 | 1.000 | 1.000 |  |
| CTRL 2 | miR-320-4395388 | 24.32 | 1.000 | 1.000 |  |
| CTRL 3 | miR-320-4395388 | 23.22 | 1.000 | 1.000 |  |
| ACM 1 | miR-483-5p-4395449 | 28.93 | 0.424 | 0.007 |  |
| ACM 2 | miR-483-5p-4395449 | 29.25 | 0.424 | 0.007 |  |
| ACM 3 | miR-483-5p-4395449 | 28.32 | 0.424 | 0.007 |  |
| CTRL 1 | miR-483-5p-4395449 | 29.13 | 1.000 | 1.000 |  |
| CTRL 2 | miR-483-5p-4395449 | 29.31 | 1.000 | 1.000 |  |
| CTRL 3 | miR-483-5p-4395449 | 28.07 | 1.000 | 1.000 |  |
| ACM 1 | miR-126-4395339 | 23.20 | 2.323 | 0.024 |  |
| ACM 2 | miR-126-4395339 | 22.05 | 2.323 | 0.024 |  |
| ACM 3 | miR-126-4395339 | 22.01 | 2.323 | 0.024 |  |
| CTRL 1 | miR-126-4395339 | 24.83 | 1.000 | 1.000 |  |
| CTRL 2 | miR-126-4395339 | 25.41 | 1.000 | 1.000 |  |
| CTRL 3 | miR-126-4395339 | 24.39 | 1.000 | 1.000 |  |
| ACM 1 | miR-301a-4373064 | 24.12 | 3.230 | 0.035 |  |
| ACM 2 | miR-301a-4373064 | 29.28 | 3.230 | 0.035 |  |
| ACM 3 | miR-301a-4373064 | 28.87 | 3.230 | 0.035 |  |
| CTRL 1 | miR-301a-4373064 | 33.27 | 1.000 | 1.000 |  |
| CTRL 2 | miR-301a-4373064 | 32.22 | 1.000 | 1.000 |  |
| CTRL 3 | miR-301a-4373064 | 31.25 | 1.000 | 1.000 |  |
| ACM 1 | miR-532-3p-4395466 | 28.10 | 1.409 | 0.038 |  |
| ACM 2 | miR-532-3p-4395466 | 27.13 | 1.409 | 0.038 |  |
| ACM 3 | miR-532-3p-4395466 | 27.02 | 1.409 | 0.038 |  |
| CTRL 1 | miR-532-3p-4395466 | 29.23 | 1.000 | 1.000 |  |
| CTRL 2 | miR-532-3p-4395466 | 29.83 | 1.000 | 1.000 |  |
| CTRL 3 | miR-532-3p-4395466 | 28.41 | 1.000 | 1.000 |  |
| ACM 1 | miR-139-5p-4395400 | 29.36 | 2.122 | 0.056 |  |
| ACM 2 | miR-139-5p-4395400 | 26.90 | 2.122 | 0.056 |  |
| ACM 3 | miR-139-5p-4395400 | 27.09 | 2.122 | 0.056 |  |
| CTRL 1 | miR-139-5p-4395400 | 29.45 | 1.000 | 1.000 |  |
| CTRL 2 | miR-139-5p-4395400 | 29.86 | 1.000 | 1.000 |  |
| CTRL 3 | miR-139-5p-4395400 | 29.36 | 1.000 | 1.000 |  |
| ACM 1 | miR-885-5p-4395407 | 27.55 | 3.360 | 0.089 |  |
| ACM 2 | miR-885-5p-4395407 | 28.05 | 3.360 | 0.089 |  |
| ACM 3 | miR-885-5p-4395407 | 26.79 | 3.360 | 0.089 |  |
| CTRL 1 | miR-885-5p-4395407 | 31.18 | 1.000 | 1.000 |  |
| CTRL 2 | miR-885-5p-4395407 | 31.63 | 1.000 | 1.000 |  |
| CTRL 3 | miR-885-5p-4395407 | 28.55 | 1.000 | 1.000 |  |
| ACM 1 | miR-30b-4373290 | 26.39 | 2.809 | 0.119 |  |
| ACM 2 | miR-30b-4373290 | 24.96 | 2.809 | 0.119 |  |
| ACM 3 | miR-30b-4373290 | 24.27 | 2.809 | 0.119 |  |
| CTRL 1 | miR-30b-4373290 | 28.28 | 1.000 | 1.000 |  |
| CTRL 2 | miR-30b-4373290 | 28.64 | 1.000 | 1.000 |  |
| CTRL 3 | miR-30b-4373290 | 26.89 | 1.000 | 1.000 |  |
| ACM 1 | miR-363-4378090 | 29.19 | 2.237 | 0.122 |  |
| ACM 2 | miR-363-4378090 | 29.11 | 2.237 | 0.122 |  |
| ACM 3 | miR-363-4378090 | 28.37 | 2.237 | 0.122 |  |
| CTRL 1 | miR-363-4378090 | 32.40 | 1.000 | 1.000 |  |
| CTRL 2 | miR-363-4378090 | 31.81 | 1.000 | 1.000 |  |
| CTRL 3 | miR-363-4378090 | 29.67 | 1.000 | 1.000 |  |
| ACM 1 | miR-744-4395435 | 30.55 | 3.993 | 0.125 |  |
| ACM 2 | miR-744-4395435 | 29.28 | 3.993 | 0.125 |  |
| ACM 3 | miR-744-4395435 | 28.33 | 3.993 | 0.125 |  |
| CTRL 1 | miR-744-4395435 | 32.11 | 1.000 | 1.000 |  |
| CTRL 2 | miR-744-4395435 | 34.50 | 1.000 | 1.000 |  |
| CTRL 3 | miR-744-4395435 | 31.26 | 1.000 | 1.000 |  |
| ACM 1 | miR-221-4373077 | 30.17 | 4.660 | 0.131 |  |
| ACM 2 | miR-221-4373077 | 27.59 | 4.660 | 0.131 |  |
| ACM 3 | miR-221-4373077 | 27.97 | 4.660 | 0.131 |  |
| CTRL 1 | miR-221-4373077 | 31.89 | 1.000 | 1.000 |  |
| CTRL 2 | miR-221-4373077 | 33.60 | 1.000 | 1.000 |  |
| CTRL 3 | miR-221-4373077 | 30.62 | 1.000 | 1.000 |  |
| ACM 1 | miR-28-3p-4395557 | 29.46 | 1.546 | 0.132 |  |
| ACM 2 | miR-28-3p-4395557 | 28.27 | 1.546 | 0.132 |  |
| ACM 3 | miR-28-3p-4395557 | 27.91 | 1.546 | 0.132 |  |
| CTRL 1 | miR-28-3p-4395557 | 30.31 | 1.000 | 1.000 |  |
| CTRL 2 | miR-28-3p-4395557 | 30.73 | 1.000 | 1.000 |  |
| CTRL 3 | miR-28-3p-4395557 | 30.21 | 1.000 | 1.000 |  |
| ACM 1 | miR-30c-4373060 | 26.44 | 4.184 | 0.143 |  |
| ACM 2 | miR-30c-4373060 | 25.09 | 4.184 | 0.143 |  |
| ACM 3 | miR-30c-4373060 | 24.06 | 4.184 | 0.143 |  |
| CTRL 1 | miR-30c-4373060 | 29.78 | 1.000 | 1.000 |  |
| CTRL 2 | miR-30c-4373060 | 29.16 | 1.000 | 1.000 |  |
| CTRL 3 | miR-30c-4373060 | 26.57 | 1.000 | 1.000 |  |
| ACM 1 | miR-27a-4373287 | 28.39 | 7.603 | 0.144 |  |
| ACM 2 | miR-27a-4373287 | 25.80 | 7.603 | 0.144 |  |
| ACM 3 | miR-27a-4373287 | 26.67 | 7.603 | 0.144 |  |
| CTRL 1 | miR-27a-4373287 | 31.76 | 1.000 | 1.000 |  |
| CTRL 2 | miR-27a-4373287 | 32.77 | 1.000 | 1.000 |  |
| CTRL 3 | miR-27a-4373287 | 28.82 | 1.000 | 1.000 |  |
| ACM 1 | miR-27b-4373068 | 29.14 | 5.401 | 0.146 |  |
| ACM 2 | miR-27b-4373068 | 26.79 | 5.401 | 0.146 |  |
| ACM 3 | miR-27b-4373068 | 27.69 | 5.401 | 0.146 |  |
| CTRL 1 | miR-27b-4373068 | 33.70 | 1.000 | 1.000 |  |
| CTRL 2 | miR-27b-4373068 | 31.11 | 1.000 | 1.000 |  |
| CTRL 3 | miR-27b-4373068 | 29.83 | 1.000 | 1.000 |  |
| ACM 1 | miR-374a-4373028 | 30.33 | 5.502 | 0.147 |  |
| ACM 2 | miR-374a-4373028 | 28.80 | 5.502 | 0.147 |  |
| ACM 3 | miR-374a-4373028 | 27.73 | 5.502 | 0.147 |  |
| CTRL 1 | miR-374a-4373028 | 32.06 | 1.000 | 1.000 |  |
| CTRL 2 | miR-374a-4373028 | 32.50 | 1.000 | 1.000 |  |
| CTRL 3 | miR-374a-4373028 | 33.41 | 1.000 | 1.000 |  |
| ACM 1 | miR-374b-4381045 | 30.15 | 2.588 | 0.147 |  |
| ACM 2 | miR-374b-4381045 | 28.35 | 2.588 | 0.147 |  |
| ACM 3 | miR-374b-4381045 | 27.97 | 2.588 | 0.147 |  |
| CTRL 1 | miR-374b-4381045 | 30.46 | 1.000 | 1.000 |  |
| CTRL 2 | miR-374b-4381045 | 32.74 | 1.000 | 1.000 |  |
| CTRL 3 | miR-374b-4381045 | 31.11 | 1.000 | 1.000 |  |
| ACM 1 | miR-16-4373121 | 21.60 | 1.142 | 0.155 |  |
| ACM 2 | miR-16-4373121 | 21.98 | 1.142 | 0.155 |  |
| ACM 3 | miR-16-4373121 | 20.28 | 1.142 | 0.155 |  |
| CTRL 1 | miR-16-4373121 | 20.73 | 1.000 | 1.000 |  |
| CTRL 2 | miR-16-4373121 | 21.48 | 1.000 | 1.000 |  |
| CTRL 3 | miR-16-4373121 | 21.02 | 1.000 | 1.000 |  |
| ACM 1 | miR-145-4395389 | 26.34 | 4.579 | 0.159 |  |
| ACM 2 | miR-145-4395389 | 23.57 | 4.579 | 0.159 |  |
| ACM 3 | miR-145-4395389 | 24.01 | 4.579 | 0.159 |  |
| CTRL 1 | miR-145-4395389 | 27.86 | 1.000 | 1.000 |  |
| CTRL 2 | miR-145-4395389 | 27.11 | 1.000 | 1.000 |  |
| CTRL 3 | miR-145-4395389 | 29.26 | 1.000 | 1.000 |  |
| ACM 1 | miR-331-3p-4373046 | 24.16 | 2.535 | 0.168 |  |
| ACM 2 | miR-331-3p-4373046 | 24.97 | 2.535 | 0.168 |  |
| ACM 3 | miR-331-3p-4373046 | 24.24 | 2.535 | 0.168 |  |
| CTRL 1 | miR-331-3p-4373046 | 26.50 | 1.000 | 1.000 |  |
| CTRL 2 | miR-331-3p-4373046 | 28.40 | 1.000 | 1.000 |  |
| CTRL 3 | miR-331-3p-4373046 | 26.22 | 1.000 | 1.000 |  |
| ACM 1 | miR-370-4395386 | 30.10 | 5.545 | 0.168 |  |
| ACM 2 | miR-370-4395386 | 27.00 | 5.545 | 0.168 |  |
| ACM 3 | miR-370-4395386 | 27.50 | 5.545 | 0.168 |  |
| CTRL 1 | miR-370-4395386 | 31.95 | 1.000 | 1.000 |  |
| CTRL 2 | miR-370-4395386 | 33.98 | 1.000 | 1.000 |  |
| CTRL 3 | miR-370-4395386 | 29.82 | 1.000 | 1.000 |  |
| ACM 1 | miR-140-5p-4373374 | 27.41 | 2.228 | 0.169 |  |
| ACM 2 | miR-140-5p-4373374 | 26.11 | 2.228 | 0.169 |  |
| ACM 3 | miR-140-5p-4373374 | 25.88 | 2.228 | 0.169 |  |
| CTRL 1 | miR-140-5p-4373374 | 29.97 | 1.000 | 1.000 |  |
| CTRL 2 | miR-140-5p-4373374 | 29.49 | 1.000 | 1.000 |  |
| CTRL 3 | miR-140-5p-4373374 | 27.13 | 1.000 | 1.000 |  |
| ACM 1 | miR-103-4373158 | 28.21 | 1.988 | 0.176 |  |
| ACM 2 | miR-103-4373158 | 26.98 | 1.988 | 0.176 |  |
| ACM 3 | miR-103-4373158 | 28.27 | 1.988 | 0.176 |  |
| CTRL 1 | miR-103-4373158 | 30.01 | 1.000 | 1.000 |  |
| CTRL 2 | miR-103-4373158 | 30.69 | 1.000 | 1.000 |  |
| CTRL 3 | miR-103-4373158 | 28.17 | 1.000 | 1.000 |  |
| ACM 1 | let-7c-4373167 | 29.71 | 1.898 | 0.180 |  |
| ACM 2 | let-7c-4373167 | 29.50 | 1.898 | 0.180 |  |
| ACM 3 | let-7c-4373167 | 28.26 | 1.898 | 0.180 |  |
| CTRL 1 | let-7c-4373167 | 31.41 | 1.000 | 1.000 |  |
| CTRL 2 | let-7c-4373167 | 31.12 | 1.000 | 1.000 |  |
| CTRL 3 | let-7c-4373167 | 31.44 | 1.000 | 1.000 |  |
| ACM 1 | miR-150-4373127 | 22.00 | 2.180 | 0.186 |  |
| ACM 2 | miR-150-4373127 | 21.83 | 2.180 | 0.186 |  |
| ACM 3 | miR-150-4373127 | 20.34 | 2.180 | 0.186 |  |
| CTRL 1 | miR-150-4373127 | 19.11 | 1.000 | 1.000 |  |
| CTRL 2 | miR-150-4373127 | 23.83 | 1.000 | 1.000 |  |
| CTRL 3 | miR-150-4373127 | 23.52 | 1.000 | 1.000 |  |
| ACM 1 | miR-28-5p-4373067 | 31.14 | 4.808 | 0.195 |  |
| ACM 2 | miR-28-5p-4373067 | 29.56 | 4.808 | 0.195 |  |
| ACM 3 | miR-28-5p-4373067 | 28.23 | 4.808 | 0.195 |  |
| CTRL 1 | miR-28-5p-4373067 | 33.47 | 1.000 | 1.000 |  |
| CTRL 2 | miR-28-5p-4373067 | 34.43 | 1.000 | 1.000 |  |
| CTRL 3 | miR-28-5p-4373067 | 31.56 | 1.000 | 1.000 |  |
| ACM 1 | miR-486-5p-4378096 | 22.48 | 0.517 | 0.224 |  |
| ACM 2 | miR-486-5p-4378096 | 22.74 | 0.517 | 0.224 |  |
| ACM 3 | miR-486-5p-4378096 | 21.98 | 0.517 | 0.224 |  |
| CTRL 1 | miR-486-5p-4378096 | 23.42 | 1.000 | 1.000 |  |
| CTRL 2 | miR-486-5p-4378096 | 23.33 | 1.000 | 1.000 |  |
| CTRL 3 | miR-486-5p-4378096 | 21.32 | 1.000 | 1.000 |  |
| ACM 1 | miR-143-4395360 | 29.32 | 3.824 | 0.226 |  |
| ACM 2 | miR-143-4395360 | 27.05 | 3.824 | 0.226 |  |
| ACM 3 | miR-143-4395360 | 28.77 | 3.824 | 0.226 |  |
| CTRL 1 | miR-143-4395360 | 32.00 | 1.000 | 1.000 |  |
| CTRL 2 | miR-143-4395360 | 32.85 | 1.000 | 1.000 |  |
| CTRL 3 | miR-143-4395360 | 29.82 | 1.000 | 1.000 |  |
| ACM 1 | miR-200a-4378069 | 31.21 | 1.451 | 0.232 |  |
| ACM 2 | miR-200a-4378069 | 29.52 | 1.451 | 0.232 |  |
| ACM 3 | miR-200a-4378069 | 29.45 | 1.451 | 0.232 |  |
| CTRL 1 | miR-200a-4378069 | 31.97 | 1.000 | 1.000 |  |
| CTRL 2 | miR-200a-4378069 | 32.29 | 1.000 | 1.000 |  |
| CTRL 3 | miR-200a-4378069 | 32.18 | 1.000 | 1.000 |  |
| ACM 1 | miR-10a-4373153 | 31.95 | 0.451 | 0.239 |  |
| ACM 2 | miR-10a-4373153 | 30.57 | 0.451 | 0.239 |  |
| ACM 3 | miR-10a-4373153 | 30.98 | 0.451 | 0.239 |  |
| CTRL 1 | miR-10a-4373153 | 31.91 | 1.000 | 1.000 |  |
| CTRL 2 | miR-10a-4373153 | 30.75 | 1.000 | 1.000 |  |
| CTRL 3 | miR-10a-4373153 | 31.12 | 1.000 | 1.000 |  |
| ACM 1 | miR-125b-4373148 | 28.26 | 7.174 | 0.244 |  |
| ACM 2 | miR-125b-4373148 | 26.49 | 7.174 | 0.244 |  |
| ACM 3 | miR-125b-4373148 | 21.91 | 7.174 | 0.244 |  |
| CTRL 1 | miR-125b-4373148 | 32.74 | 1.000 | 1.000 |  |
| CTRL 2 | miR-125b-4373148 | 22.41 | 1.000 | 1.000 |  |
| CTRL 3 | miR-125b-4373148 | 29.46 | 1.000 | 1.000 |  |
| ACM 1 | miR-194-4373106 | 29.03 | 2.797 | 0.248 |  |
| ACM 2 | miR-194-4373106 | 27.92 | 2.797 | 0.248 |  |
| ACM 3 | miR-194-4373106 | 26.36 | 2.797 | 0.248 |  |
| CTRL 1 | miR-194-4373106 | 30.17 | 1.000 | 1.000 |  |
| CTRL 2 | miR-194-4373106 | 30.77 | 1.000 | 1.000 |  |
| CTRL 3 | miR-194-4373106 | 30.54 | 1.000 | 1.000 |  |
| ACM 1 | miR-486-3p-4395204 | 27.35 | 0.531 | 0.250 |  |
| ACM 2 | miR-486-3p-4395204 | 28.08 | 0.531 | 0.250 |  |
| ACM 3 | miR-486-3p-4395204 | 26.81 | 0.531 | 0.250 |  |
| CTRL 1 | miR-486-3p-4395204 | 27.98 | 1.000 | 1.000 |  |
| CTRL 2 | miR-486-3p-4395204 | 28.86 | 1.000 | 1.000 |  |
| CTRL 3 | miR-486-3p-4395204 | 26.38 | 1.000 | 1.000 |  |
| ACM 1 | miR-324-3p-4395272 | 26.87 | 0.561 | 0.252 |  |
| ACM 2 | miR-324-3p-4395272 | 27.80 | 0.561 | 0.252 |  |
| ACM 3 | miR-324-3p-4395272 | 26.22 | 0.561 | 0.252 |  |
| CTRL 1 | miR-324-3p-4395272 | 27.71 | 1.000 | 1.000 |  |
| CTRL 2 | miR-324-3p-4395272 | 28.27 | 1.000 | 1.000 |  |
| CTRL 3 | miR-324-3p-4395272 | 26.13 | 1.000 | 1.000 |  |
| ACM 1 | miR-142-3p-4373136 | 28.30 | 6.714 | 0.257 |  |
| ACM 2 | miR-142-3p-4373136 | 26.64 | 6.714 | 0.257 |  |
| ACM 3 | miR-142-3p-4373136 | 24.82 | 6.714 | 0.257 |  |
| CTRL 1 | miR-142-3p-4373136 | 32.56 | 1.000 | 1.000 |  |
| CTRL 2 | miR-142-3p-4373136 | 30.56 | 1.000 | 1.000 |  |
| CTRL 3 | miR-142-3p-4373136 | 28.61 | 1.000 | 1.000 |  |
| ACM 1 | miR-214-4395417 | 30.06 | 1.617 | 0.258 |  |
| ACM 2 | miR-214-4395417 | 28.24 | 1.617 | 0.258 |  |
| ACM 3 | miR-214-4395417 | 29.09 | 1.617 | 0.258 |  |
| CTRL 1 | miR-214-4395417 | 30.97 | 1.000 | 1.000 |  |
| CTRL 2 | miR-214-4395417 | 31.80 | 1.000 | 1.000 |  |
| CTRL 3 | miR-214-4395417 | 30.43 | 1.000 | 1.000 |  |
| ACM 1 | miR-26a-4395166 | 33.16 | 2.754 | 0.272 |  |
| ACM 2 | miR-26a-4395166 | 28.92 | 2.754 | 0.272 |  |
| ACM 3 | miR-26a-4395166 | 28.00 | 2.754 | 0.272 |  |
| CTRL 1 | miR-26a-4395166 | 34.11 | 1.000 | 1.000 |  |
| CTRL 2 | miR-26a-4395166 | 32.52 | 1.000 | 1.000 |  |
| CTRL 3 | miR-26a-4395166 | 31.57 | 1.000 | 1.000 |  |
| ACM 1 | miR-101-4395364 | 31.58 | 3.192 | 0.283 |  |
| ACM 2 | miR-101-4395364 | 30.46 | 3.192 | 0.283 |  |
| ACM 3 | miR-101-4395364 | 29.03 | 3.192 | 0.283 |  |
| CTRL 1 | miR-101-4395364 | 34.44 | 1.000 | 1.000 |  |
| CTRL 2 | miR-101-4395364 | 34.45 | 1.000 | 1.000 |  |
| CTRL 3 | miR-101-4395364 | 30.93 | 1.000 | 1.000 |  |
| ACM 1 | miR-642-4380995 | 30.40 | 1.440 | 0.303 |  |
| ACM 2 | miR-642-4380995 | 28.95 | 1.440 | 0.303 |  |
| ACM 3 | miR-642-4380995 | 29.16 | 1.440 | 0.303 |  |
| CTRL 1 | miR-642-4380995 | 30.84 | 1.000 | 1.000 |  |
| CTRL 2 | miR-642-4380995 | 31.57 | 1.000 | 1.000 |  |
| CTRL 3 | miR-642-4380995 | 31.41 | 1.000 | 1.000 |  |
| ACM 1 | miR-139-3p-4395424 | 28.58 | 1.561 | 0.304 |  |
| ACM 2 | miR-139-3p-4395424 | 27.77 | 1.561 | 0.304 |  |
| ACM 3 | miR-139-3p-4395424 | 29.05 | 1.561 | 0.304 |  |
| CTRL 1 | miR-139-3p-4395424 | 31.38 | 1.000 | 1.000 |  |
| CTRL 2 | miR-139-3p-4395424 | 30.34 | 1.000 | 1.000 |  |
| CTRL 3 | miR-139-3p-4395424 | 29.33 | 1.000 | 1.000 |  |
| ACM 1 | miR-339-3p-4395295 | 30.09 | 1.573 | 0.318 |  |
| ACM 2 | miR-339-3p-4395295 | 29.05 | 1.573 | 0.318 |  |
| ACM 3 | miR-339-3p-4395295 | 28.84 | 1.573 | 0.318 |  |
| CTRL 1 | miR-339-3p-4395295 | 31.68 | 1.000 | 1.000 |  |
| CTRL 2 | miR-339-3p-4395295 | 32.23 | 1.000 | 1.000 |  |
| CTRL 3 | miR-339-3p-4395295 | 29.77 | 1.000 | 1.000 |  |
| ACM 1 | miR-133a-4395357 | 30.20 | 6.660 | 0.319 |  |
| ACM 2 | miR-133a-4395357 | 25.59 | 6.660 | 0.319 |  |
| ACM 3 | miR-133a-4395357 | 27.77 | 6.660 | 0.319 |  |
| CTRL 1 | miR-133a-4395357 | 31.48 | 1.000 | 1.000 |  |
| CTRL 2 | miR-133a-4395357 | 33.35 | 1.000 | 1.000 |  |
| CTRL 3 | miR-133a-4395357 | 30.66 | 1.000 | 1.000 |  |
| ACM 1 | miR-342-3p-4395371 | 22.44 | 0.555 | 0.321 |  |
| ACM 2 | miR-342-3p-4395371 | 21.49 | 0.555 | 0.321 |  |
| ACM 3 | miR-342-3p-4395371 | 21.58 | 0.555 | 0.321 |  |
| CTRL 1 | miR-342-3p-4395371 | 21.28 | 1.000 | 1.000 |  |
| CTRL 2 | miR-342-3p-4395371 | 22.66 | 1.000 | 1.000 |  |
| CTRL 3 | miR-342-3p-4395371 | 22.74 | 1.000 | 1.000 |  |
| ACM 1 | miR-132-4373143 | 29.34 | 1.845 | 0.322 |  |
| ACM 2 | miR-132-4373143 | 28.40 | 1.845 | 0.322 |  |
| ACM 3 | miR-132-4373143 | 27.69 | 1.845 | 0.322 |  |
| CTRL 1 | miR-132-4373143 | 30.53 | 1.000 | 1.000 |  |
| CTRL 2 | miR-132-4373143 | 32.31 | 1.000 | 1.000 |  |
| CTRL 3 | miR-132-4373143 | 28.95 | 1.000 | 1.000 |  |
| ACM 1 | miR-195-4373105 | 27.20 | 4.072 | 0.328 |  |
| ACM 2 | miR-195-4373105 | 34.97 | 4.072 | 0.328 |  |
| ACM 3 | miR-195-4373105 | 25.14 | 4.072 | 0.328 |  |
| CTRL 1 | miR-195-4373105 | 30.83 | 1.000 | 1.000 |  |
| CTRL 2 | miR-195-4373105 | 28.45 | 1.000 | 1.000 |  |
| CTRL 3 | miR-195-4373105 | 34.45 | 1.000 | 1.000 |  |
| ACM 1 | miR-328-4373049 | 28.90 | 4.934 | 0.336 |  |
| ACM 2 | miR-328-4373049 | 26.65 | 4.934 | 0.336 |  |
| ACM 3 | miR-328-4373049 | 28.85 | 4.934 | 0.336 |  |
| CTRL 1 | miR-328-4373049 | 33.08 | 1.000 | 1.000 |  |
| CTRL 2 | miR-328-4373049 | 33.43 | 1.000 | 1.000 |  |
| CTRL 3 | miR-328-4373049 | 28.52 | 1.000 | 1.000 |  |
| ACM 1 | miR-365-4373194 | 28.45 | 3.103 | 0.349 |  |
| ACM 2 | miR-365-4373194 | 25.35 | 3.103 | 0.349 |  |
| ACM 3 | miR-365-4373194 | 27.14 | 3.103 | 0.349 |  |
| CTRL 1 | miR-365-4373194 | 31.09 | 1.000 | 1.000 |  |
| CTRL 2 | miR-365-4373194 | 30.85 | 1.000 | 1.000 |  |
| CTRL 3 | miR-365-4373194 | 27.63 | 1.000 | 1.000 |  |
| ACM 1 | miR-15b-4373122 | 26.49 | 2.568 | 0.351 |  |
| ACM 2 | miR-15b-4373122 | 24.63 | 2.568 | 0.351 |  |
| ACM 3 | miR-15b-4373122 | 24.39 | 2.568 | 0.351 |  |
| CTRL 1 | miR-15b-4373122 | 28.31 | 1.000 | 1.000 |  |
| CTRL 2 | miR-15b-4373122 | 28.31 | 1.000 | 1.000 |  |
| CTRL 3 | miR-15b-4373122 | 26.77 | 1.000 | 1.000 |  |
| ACM 1 | miR-152-4395170 | 30.18 | 1.961 | 0.362 |  |
| ACM 2 | miR-152-4395170 | 29.64 | 1.961 | 0.362 |  |
| ACM 3 | miR-152-4395170 | 29.51 | 1.961 | 0.362 |  |
| CTRL 1 | miR-152-4395170 | 33.87 | 1.000 | 1.000 |  |
| CTRL 2 | miR-152-4395170 | 31.78 | 1.000 | 1.000 |  |
| CTRL 3 | miR-152-4395170 | 30.31 | 1.000 | 1.000 |  |
| ACM 1 | miR-376a-4373026 | 32.46 | 2.620 | 0.362 |  |
| ACM 2 | miR-376a-4373026 | 27.69 | 2.620 | 0.362 |  |
| ACM 3 | miR-376a-4373026 | 29.39 | 2.620 | 0.362 |  |
| CTRL 1 | miR-376a-4373026 | 34.37 | 1.000 | 1.000 |  |
| CTRL 2 | miR-376a-4373026 | 33.79 | 1.000 | 1.000 |  |
| CTRL 3 | miR-376a-4373026 | 31.02 | 1.000 | 1.000 |  |
| ACM 1 | miR-345-4395297 | 28.67 | 2.050 | 0.366 |  |
| ACM 2 | miR-345-4395297 | 26.57 | 2.050 | 0.366 |  |
| ACM 3 | miR-345-4395297 | 27.45 | 2.050 | 0.366 |  |
| CTRL 1 | miR-345-4395297 | 30.64 | 1.000 | 1.000 |  |
| CTRL 2 | miR-345-4395297 | 31.00 | 1.000 | 1.000 |  |
| CTRL 3 | miR-345-4395297 | 27.89 | 1.000 | 1.000 |  |
| ACM 1 | miR-21-4373090 | 21.47 | 2.404 | 0.370 |  |
| ACM 2 | miR-21-4373090 | 23.54 | 2.404 | 0.370 |  |
| ACM 3 | miR-21-4373090 | 22.85 | 2.404 | 0.370 |  |
| CTRL 1 | miR-21-4373090 | 27.26 | 1.000 | 1.000 |  |
| CTRL 2 | miR-21-4373090 | 26.83 | 1.000 | 1.000 |  |
| CTRL 3 | miR-21-4373090 | 23.72 | 1.000 | 1.000 |  |
| ACM 1 | miR-146b-5p-4373178 | 27.34 | 1.775 | 0.373 |  |
| ACM 2 | miR-146b-5p-4373178 | 26.25 | 1.775 | 0.373 |  |
| ACM 3 | miR-146b-5p-4373178 | 24.69 | 1.775 | 0.373 |  |
| CTRL 1 | miR-146b-5p-4373178 | 27.56 | 1.000 | 1.000 |  |
| CTRL 2 | miR-146b-5p-4373178 | 29.21 | 1.000 | 1.000 |  |
| CTRL 3 | miR-146b-5p-4373178 | 27.71 | 1.000 | 1.000 |  |
| ACM 1 | miR-629-4395547 | 31.27 | 1.291 | 0.395 |  |
| ACM 2 | miR-629-4395547 | 34.60 | 1.291 | 0.395 |  |
| ACM 3 | miR-629-4395547 | 29.95 | 1.291 | 0.395 |  |
| CTRL 1 | miR-629-4395547 | 32.74 | 1.000 | 1.000 |  |
| CTRL 2 | miR-629-4395547 | 34.33 | 1.000 | 1.000 |  |
| CTRL 3 | miR-629-4395547 | 33.58 | 1.000 | 1.000 |  |
| ACM 1 | miR-26b-4395167 | 29.49 | 2.720 | 0.397 |  |
| ACM 2 | miR-26b-4395167 | 29.24 | 2.720 | 0.397 |  |
| ACM 3 | miR-26b-4395167 | 29.76 | 2.720 | 0.397 |  |
| CTRL 1 | miR-26b-4395167 | 28.25 | 1.000 | 1.000 |  |
| CTRL 2 | miR-26b-4395167 | 33.95 | 1.000 | 1.000 |  |
| CTRL 3 | miR-26b-4395167 | 30.26 | 1.000 | 1.000 |  |
| ACM 1 | miR-141-4373137 | 32.01 | 1.798 | 0.408 |  |
| ACM 2 | miR-141-4373137 | 32.74 | 1.798 | 0.408 |  |
| ACM 3 | miR-141-4373137 | 29.31 | 1.798 | 0.408 |  |
| CTRL 1 | miR-141-4373137 | 22.37 | 1.000 | 1.000 |  |
| CTRL 2 | miR-141-4373137 | 33.92 | 1.000 | 1.000 |  |
| CTRL 3 | miR-141-4373137 | 32.80 | 1.000 | 1.000 |  |
| ACM 1 | miR-34a-4395168 | 32.19 | 1.595 | 0.415 |  |
| ACM 2 | miR-34a-4395168 | 30.04 | 1.595 | 0.415 |  |
| ACM 3 | miR-34a-4395168 | 29.76 | 1.595 | 0.415 |  |
| CTRL 1 | miR-34a-4395168 | 33.40 | 1.000 | 1.000 |  |
| CTRL 2 | miR-34a-4395168 | 20.56 | 1.000 | 1.000 |  |
| CTRL 3 | miR-34a-4395168 | 31.31 | 1.000 | 1.000 |  |
| ACM 1 | has-miR-155-4395459 | 28.40 | 0.858 | 0.456 |  |
| ACM 2 | has-miR-155-4395459 | 28.05 | 0.858 | 0.456 |  |
| ACM 3 | has-miR-155-4395459 | 27.66 | 0.858 | 0.456 |  |
| CTRL 1 | has-miR-155-4395459 | 28.52 | 1.000 | 1.000 |  |
| CTRL 2 | has-miR-155-4395459 | 28.01 | 1.000 | 1.000 |  |
| CTRL 3 | has-miR-155-4395459 | 30.65 | 1.000 | 1.000 |  |
| ACM 1 | miR-210-4373089 | 26.97 | 1.044 | 0.456 |  |
| ACM 2 | miR-210-4373089 | 25.72 | 1.044 | 0.456 |  |
| ACM 3 | miR-210-4373089 | 24.86 | 1.044 | 0.456 |  |
| CTRL 1 | miR-210-4373089 | 29.28 | 1.000 | 1.000 |  |
| CTRL 2 | miR-210-4373089 | 28.51 | 1.000 | 1.000 |  |
| CTRL 3 | miR-210-4373089 | 26.05 | 1.000 | 1.000 |  |
| ACM 1 | miR-125a-5p-4395309 | 31.54 | 1.142 | 0.483 |  |
| ACM 2 | miR-125a-5p-4395309 | 30.88 | 1.142 | 0.483 |  |
| ACM 3 | miR-125a-5p-4395309 | 30.58 | 1.142 | 0.483 |  |
| CTRL 1 | miR-125a-5p-4395309 | 32.30 | 1.000 | 1.000 |  |
| CTRL 2 | miR-125a-5p-4395309 | 33.28 | 1.000 | 1.000 |  |
| CTRL 3 | miR-125a-5p-4395309 | 31.72 | 1.000 | 1.000 |  |
| ACM 1 | miR-19b-4373098 | 19.38 | 0.739 | 0.493 |  |
| ACM 2 | miR-19b-4373098 | 19.15 | 0.739 | 0.493 |  |
| ACM 3 | miR-19b-4373098 | 16.87 | 0.739 | 0.493 |  |
| CTRL 1 | miR-19b-4373098 | 20.69 | 1.000 | 1.000 |  |
| CTRL 2 | miR-19b-4373098 | 20.96 | 1.000 | 1.000 |  |
| CTRL 3 | miR-19b-4373098 | 18.12 | 1.000 | 1.000 |  |
| ACM 1 | miR-501-5p-4373226 | 27.88 | 0.650 | 0.494 |  |
| ACM 2 | miR-501-5p-4373226 | 28.95 | 0.650 | 0.494 |  |
| ACM 3 | miR-501-5p-4373226 | 28.54 | 0.650 | 0.494 |  |
| CTRL 1 | miR-501-5p-4373226 | 29.68 | 1.000 | 1.000 |  |
| CTRL 2 | miR-501-5p-4373226 | 28.70 | 1.000 | 1.000 |  |
| CTRL 3 | miR-501-5p-4373226 | 28.85 | 1.000 | 1.000 |  |
| ACM 1 | miR-106b-4373155 | 24.80 | 1.885 | 0.500 |  |
| ACM 2 | miR-106b-4373155 | 24.19 | 1.885 | 0.500 |  |
| ACM 3 | miR-106b-4373155 | 23.90 | 1.885 | 0.500 |  |
| CTRL 1 | miR-106b-4373155 | 27.80 | 1.000 | 1.000 |  |
| CTRL 2 | miR-106b-4373155 | 27.11 | 1.000 | 1.000 |  |
| CTRL 3 | miR-106b-4373155 | 24.44 | 1.000 | 1.000 |  |
| ACM 1 | miR-204-4373094 | 30.79 | 1.375 | 0.506 |  |
| ACM 2 | miR-204-4373094 | 27.16 | 1.375 | 0.506 |  |
| ACM 3 | miR-204-4373094 | 30.24 | 1.375 | 0.506 |  |
| CTRL 1 | miR-204-4373094 | 34.74 | 1.000 | 1.000 |  |
| CTRL 2 | miR-204-4373094 | 31.68 | 1.000 | 1.000 |  |
| CTRL 3 | miR-204-4373094 | 30.36 | 1.000 | 1.000 |  |
| ACM 1 | let-7g-4395393 | 29.50 | 0.724 | 0.507 |  |
| ACM 2 | let-7g-4395393 | 29.58 | 0.724 | 0.507 |  |
| ACM 3 | let-7g-4395393 | 27.26 | 0.724 | 0.507 |  |
| CTRL 1 | let-7g-4395393 | 27.62 | 1.000 | 1.000 |  |
| CTRL 2 | let-7g-4395393 | 29.71 | 1.000 | 1.000 |  |
| CTRL 3 | let-7g-4395393 | 31.34 | 1.000 | 1.000 |  |
| ACM 1 | miR-296-5p-4373066 | 31.20 | 0.396 | 0.526 |  |
| ACM 2 | miR-296-5p-4373066 | 31.68 | 0.396 | 0.526 |  |
| ACM 3 | miR-296-5p-4373066 | 31.06 | 0.396 | 0.526 |  |
| CTRL 1 | miR-296-5p-4373066 | 31.98 | 1.000 | 1.000 |  |
| CTRL 2 | miR-296-5p-4373066 | 33.33 | 1.000 | 1.000 |  |
| CTRL 3 | miR-296-5p-4373066 | 28.92 | 1.000 | 1.000 |  |
| ACM 1 | miR-134-4373299 | 29.34 | 0.988 | 0.533 |  |
| ACM 2 | miR-134-4373299 | 28.77 | 0.988 | 0.533 |  |
| ACM 3 | miR-134-4373299 | 28.38 | 0.988 | 0.533 |  |
| CTRL 1 | miR-134-4373299 | 28.75 | 1.000 | 1.000 |  |
| CTRL 2 | miR-134-4373299 | 29.76 | 1.000 | 1.000 |  |
| CTRL 3 | miR-134-4373299 | 31.64 | 1.000 | 1.000 |  |
| ACM 1 | miR-181a-4373117 | 31.58 | 1.837 | 0.536 |  |
| ACM 2 | miR-181a-4373117 | 30.29 | 1.837 | 0.536 |  |
| ACM 3 | miR-181a-4373117 | 28.05 | 1.837 | 0.536 |  |
| CTRL 1 | miR-181a-4373117 | 31.11 | 1.000 | 1.000 |  |
| CTRL 2 | miR-181a-4373117 | 32.14 | 1.000 | 1.000 |  |
| CTRL 3 | miR-181a-4373117 | 31.32 | 1.000 | 1.000 |  |
| ACM 1 | miR-886-5p-4395304 | 31.31 | 0.566 | 0.547 |  |
| ACM 2 | miR-886-5p-4395304 | 33.15 | 0.566 | 0.547 |  |
| ACM 3 | miR-886-5p-4395304 | 30.01 | 0.566 | 0.547 |  |
| CTRL 1 | miR-886-5p-4395304 | 32.76 | 1.000 | 1.000 |  |
| CTRL 2 | miR-886-5p-4395304 | 31.14 | 1.000 | 1.000 |  |
| CTRL 3 | miR-886-5p-4395304 | 31.83 | 1.000 | 1.000 |  |
| ACM 1 | miR-18a-4395533 | 26.98 | 1.270 | 0.551 |  |
| ACM 2 | miR-18a-4395533 | 28.33 | 1.270 | 0.551 |  |
| ACM 3 | miR-18a-4395533 | 25.45 | 1.270 | 0.551 |  |
| CTRL 1 | miR-18a-4395533 | 29.53 | 1.000 | 1.000 |  |
| CTRL 2 | miR-18a-4395533 | 28.07 | 1.000 | 1.000 |  |
| CTRL 3 | miR-18a-4395533 | 27.92 | 1.000 | 1.000 |  |
| ACM 1 | miR-218-4373081 | 30.28 | 1.172 | 0.552 |  |
| ACM 2 | miR-218-4373081 | 28.19 | 1.172 | 0.552 |  |
| ACM 3 | miR-218-4373081 | 28.85 | 1.172 | 0.552 |  |
| CTRL 1 | miR-218-4373081 | 32.93 | 1.000 | 1.000 |  |
| CTRL 2 | miR-218-4373081 | 32.14 | 1.000 | 1.000 |  |
| CTRL 3 | miR-218-4373081 | 26.67 | 1.000 | 1.000 |  |
| ACM 1 | miR-20b-4373263 | 25.98 | 1.541 | 0.554 |  |
| ACM 2 | miR-20b-4373263 | 26.69 | 1.541 | 0.554 |  |
| ACM 3 | miR-20b-4373263 | 23.92 | 1.541 | 0.554 |  |
| CTRL 1 | miR-20b-4373263 | 28.30 | 1.000 | 1.000 |  |
| CTRL 2 | miR-20b-4373263 | 28.40 | 1.000 | 1.000 |  |
| CTRL 3 | miR-20b-4373263 | 25.48 | 1.000 | 1.000 |  |
| ACM 1 | miR-500-4395539 | 34.96 | 1.044 | 0.556 |  |
| ACM 2 | miR-500-4395539 | 31.29 | 1.044 | 0.556 |  |
| ACM 3 | miR-500-4395539 | 31.76 | 1.044 | 0.556 |  |
| CTRL 1 | miR-500-4395539 | 34.02 | 1.000 | 1.000 |  |
| CTRL 2 | miR-500-4395539 | 34.51 | 1.000 | 1.000 |  |
| CTRL 3 | miR-500-4395539 | 31.74 | 1.000 | 1.000 |  |
| ACM 1 | miR-193a-5p-4395392 | 28.93 | 1.760 | 0.564 |  |
| ACM 2 | miR-193a-5p-4395392 | 27.98 | 1.760 | 0.564 |  |
| ACM 3 | miR-193a-5p-4395392 | 28.22 | 1.760 | 0.564 |  |
| CTRL 1 | miR-193a-5p-4395392 | 30.96 | 1.000 | 1.000 |  |
| CTRL 2 | miR-193a-5p-4395392 | 31.89 | 1.000 | 1.000 |  |
| CTRL 3 | miR-193a-5p-4395392 | 28.44 | 1.000 | 1.000 |  |
| ACM 1 | miR-106a-4395280 | 23.17 | 0.921 | 0.566 |  |
| ACM 2 | miR-106a-4395280 | 23.13 | 0.921 | 0.566 |  |
| ACM 3 | miR-106a-4395280 | 22.38 | 0.921 | 0.566 |  |
| CTRL 1 | miR-106a-4395280 | 25.95 | 1.000 | 1.000 |  |
| CTRL 2 | miR-106a-4395280 | 24.14 | 1.000 | 1.000 |  |
| CTRL 3 | miR-106a-4395280 | 21.96 | 1.000 | 1.000 |  |
| ACM 1 | miR-886-3p-4395305 | 31.27 | 1.642 | 0.585 |  |
| ACM 2 | miR-886-3p-4395305 | 34.85 | 1.642 | 0.585 |  |
| ACM 3 | miR-886-3p-4395305 | 33.19 | 1.642 | 0.585 |  |
| CTRL 1 | miR-886-3p-4395305 | 31.16 | 1.000 | 1.000 |  |
| CTRL 2 | miR-886-3p-4395305 | 33.99 | 1.000 | 1.000 |  |
| CTRL 3 | miR-886-3p-4395305 | 34.04 | 1.000 | 1.000 |  |
| ACM 1 | miR-375-4373027 | 24.14 | 1.034 | 0.586 |  |
| ACM 2 | miR-375-4373027 | 25.75 | 1.034 | 0.586 |  |
| ACM 3 | miR-375-4373027 | 23.24 | 1.034 | 0.586 |  |
| CTRL 1 | miR-375-4373027 | 27.40 | 1.000 | 1.000 |  |
| CTRL 2 | miR-375-4373027 | 27.83 | 1.000 | 1.000 |  |
| CTRL 3 | miR-375-4373027 | 24.09 | 1.000 | 1.000 |  |
| ACM 1 | miR-222-4395387 | 24.70 | 1.711 | 0.609 |  |
| ACM 2 | miR-222-4395387 | 23.56 | 1.711 | 0.609 |  |
| ACM 3 | miR-222-4395387 | 23.95 | 1.711 | 0.609 |  |
| CTRL 1 | miR-222-4395387 | 27.60 | 1.000 | 1.000 |  |
| CTRL 2 | miR-222-4395387 | 26.55 | 1.000 | 1.000 |  |
| CTRL 3 | miR-222-4395387 | 24.12 | 1.000 | 1.000 |  |
| ACM 1 | miR-186-4395396 | 27.34 | 0.888 | 0.619 |  |
| ACM 2 | miR-186-4395396 | 26.65 | 0.888 | 0.619 |  |
| ACM 3 | miR-186-4395396 | 26.47 | 0.888 | 0.619 |  |
| CTRL 1 | miR-186-4395396 | 28.41 | 1.000 | 1.000 |  |
| CTRL 2 | miR-186-4395396 | 28.95 | 1.000 | 1.000 |  |
| CTRL 3 | miR-186-4395396 | 26.31 | 1.000 | 1.000 |  |
| ACM 1 | miR-576-3p-4395462 | 34.03 | 0.727 | 0.625 |  |
| ACM 2 | miR-576-3p-4395462 | 30.98 | 0.727 | 0.625 |  |
| ACM 3 | miR-576-3p-4395462 | 32.50 | 0.727 | 0.625 |  |
| CTRL 1 | miR-576-3p-4395462 | 33.90 | 1.000 | 1.000 |  |
| CTRL 2 | miR-576-3p-4395462 | 34.99 | 1.000 | 1.000 |  |
| CTRL 3 | miR-576-3p-4395462 | 30.96 | 1.000 | 1.000 |  |
| ACM 1 | miR-29a-4395223 | 27.38 | 1.801 | 0.639 |  |
| ACM 2 | miR-29a-4395223 | 26.11 | 1.801 | 0.639 |  |
| ACM 3 | miR-29a-4395223 | 25.75 | 1.801 | 0.639 |  |
| CTRL 1 | miR-29a-4395223 | 29.22 | 1.000 | 1.000 |  |
| CTRL 2 | miR-29a-4395223 | 29.97 | 1.000 | 1.000 |  |
| CTRL 3 | miR-29a-4395223 | 26.32 | 1.000 | 1.000 |  |
| ACM 1 | miR-122-4395356 | 25.29 | 1.090 | 0.644 |  |
| ACM 2 | miR-122-4395356 | 26.64 | 1.090 | 0.644 |  |
| ACM 3 | miR-122-4395356 | 24.81 | 1.090 | 0.644 |  |
| CTRL 1 | miR-122-4395356 | 27.60 | 1.000 | 1.000 |  |
| CTRL 2 | miR-122-4395356 | 27.36 | 1.000 | 1.000 |  |
| CTRL 3 | miR-122-4395356 | 25.88 | 1.000 | 1.000 |  |
| ACM 1 | miR-17-4395419 | 23.15 | 0.978 | 0.653 |  |
| ACM 2 | miR-17-4395419 | 23.19 | 0.978 | 0.653 |  |
| ACM 3 | miR-17-4395419 | 22.00 | 0.978 | 0.653 |  |
| CTRL 1 | miR-17-4395419 | 25.77 | 1.000 | 1.000 |  |
| CTRL 2 | miR-17-4395419 | 24.34 | 1.000 | 1.000 |  |
| CTRL 3 | miR-17-4395419 | 21.85 | 1.000 | 1.000 |  |
| ACM 1 | miR-130b-4373144 | 29.19 | 0.814 | 0.655 |  |
| ACM 2 | miR-130b-4373144 | 29.15 | 0.814 | 0.655 |  |
| ACM 3 | miR-130b-4373144 | 26.65 | 0.814 | 0.655 |  |
| CTRL 1 | miR-130b-4373144 | 31.54 | 1.000 | 1.000 |  |
| CTRL 2 | miR-130b-4373144 | 26.87 | 1.000 | 1.000 |  |
| CTRL 3 | miR-130b-4373144 | 27.97 | 1.000 | 1.000 |  |
| ACM 1 | miR-652-4395463 | 30.91 | 0.794 | 0.667 |  |
| ACM 2 | miR-652-4395463 | 30.66 | 0.794 | 0.667 |  |
| ACM 3 | miR-652-4395463 | 29.05 | 0.794 | 0.667 |  |
| CTRL 1 | miR-652-4395463 | 30.80 | 1.000 | 1.000 |  |
| CTRL 2 | miR-652-4395463 | 31.42 | 1.000 | 1.000 |  |
| CTRL 3 | miR-652-4395463 | 31.13 | 1.000 | 1.000 |  |
| ACM 1 | miR-590-5p-4395176 | 32.16 | 1.604 | 0.669 |  |
| ACM 2 | miR-590-5p-4395176 | 30.97 | 1.604 | 0.669 |  |
| ACM 3 | miR-590-5p-4395176 | 29.91 | 1.604 | 0.669 |  |
| CTRL 1 | miR-590-5p-4395176 | 34.41 | 1.000 | 1.000 |  |
| CTRL 2 | miR-590-5p-4395176 | 33.49 | 1.000 | 1.000 |  |
| CTRL 3 | miR-590-5p-4395176 | 30.90 | 1.000 | 1.000 |  |
| ACM 1 | miR-193b-4395478 | 27.32 | 1.437 | 0.674 |  |
| ACM 2 | miR-193b-4395478 | 27.18 | 1.437 | 0.674 |  |
| ACM 3 | miR-193b-4395478 | 27.62 | 1.437 | 0.674 |  |
| CTRL 1 | miR-193b-4395478 | 28.15 | 1.000 | 1.000 |  |
| CTRL 2 | miR-193b-4395478 | 30.75 | 1.000 | 1.000 |  |
| CTRL 3 | miR-193b-4395478 | 28.51 | 1.000 | 1.000 |  |
| ACM 1 | miR-24-4373072 | 22.92 | 1.205 | 0.679 |  |
| ACM 2 | miR-24-4373072 | 20.76 | 1.205 | 0.679 |  |
| ACM 3 | miR-24-4373072 | 19.01 | 1.205 | 0.679 |  |
| CTRL 1 | miR-24-4373072 | 23.10 | 1.000 | 1.000 |  |
| CTRL 2 | miR-24-4373072 | 23.88 | 1.000 | 1.000 |  |
| CTRL 3 | miR-24-4373072 | 22.66 | 1.000 | 1.000 |  |
| ACM 1 | miR-191-4395410 | 24.89 | 0.885 | 0.686 |  |
| ACM 2 | miR-191-4395410 | 24.10 | 0.885 | 0.686 |  |
| ACM 3 | miR-191-4395410 | 23.03 | 0.885 | 0.686 |  |
| CTRL 1 | miR-191-4395410 | 24.23 | 1.000 | 1.000 |  |
| CTRL 2 | miR-191-4395410 | 25.51 | 1.000 | 1.000 |  |
| CTRL 3 | miR-191-4395410 | 25.48 | 1.000 | 1.000 |  |
| ACM 1 | miR-184-4373113 | 33.53 | 0.553 | 0.696 |  |
| ACM 2 | miR-184-4373113 | 33.45 | 0.553 | 0.696 |  |
| ACM 3 | miR-184-4373113 | 28.26 | 0.553 | 0.696 |  |
| CTRL 1 | miR-184-4373113 | 32.84 | 1.000 | 1.000 |  |
| CTRL 2 | miR-184-4373113 | 32.01 | 1.000 | 1.000 |  |
| CTRL 3 | miR-184-4373113 | 29.37 | 1.000 | 1.000 |  |
| ACM 1 | miR-128-4395327 | 32.54 | 0.844 | 0.707 |  |
| ACM 2 | miR-128-4395327 | 31.02 | 0.844 | 0.707 |  |
| ACM 3 | miR-128-4395327 | 30.82 | 0.844 | 0.707 |  |
| CTRL 1 | miR-128-4395327 | 31.56 | 1.000 | 1.000 |  |
| CTRL 2 | miR-128-4395327 | 33.92 | 1.000 | 1.000 |  |
| CTRL 3 | miR-128-4395327 | 30.84 | 1.000 | 1.000 |  |
| ACM 1 | miR-192-4373108 | 26.24 | 3.756 | 0.710 |  |
| ACM 2 | miR-192-4373108 | 27.06 | 3.756 | 0.710 |  |
| ACM 3 | miR-192-4373108 | 26.07 | 3.756 | 0.710 |  |
| CTRL 1 | miR-192-4373108 | 34.11 | 1.000 | 1.000 |  |
| CTRL 2 | miR-192-4373108 | 28.37 | 1.000 | 1.000 |  |
| CTRL 3 | miR-192-4373108 | 26.34 | 1.000 | 1.000 |  |
| ACM 1 | miR-636-4395199 | 31.82 | 0.862 | 0.745 |  |
| ACM 2 | miR-636-4395199 | 31.93 | 0.862 | 0.745 |  |
| ACM 3 | miR-636-4395199 | 32.10 | 0.862 | 0.745 |  |
| CTRL 1 | miR-636-4395199 | 33.67 | 1.000 | 1.000 |  |
| CTRL 2 | miR-636-4395199 | 34.21 | 1.000 | 1.000 |  |
| CTRL 3 | miR-636-4395199 | 31.85 | 1.000 | 1.000 |  |
| ACM 1 | miR-205-4373093 | 31.98 | 0.883 | 0.764 |  |
| ACM 2 | miR-205-4373093 | 30.56 | 0.883 | 0.764 |  |
| ACM 3 | miR-205-4373093 | 30.06 | 0.883 | 0.764 |  |
| CTRL 1 | miR-205-4373093 | 34.13 | 1.000 | 1.000 |  |
| CTRL 2 | miR-205-4373093 | 33.19 | 1.000 | 1.000 |  |
| CTRL 3 | miR-205-4373093 | 30.51 | 1.000 | 1.000 |  |
| ACM 1 | miR-340-4395369 | 31.19 | 1.519 | 0.780 |  |
| ACM 2 | miR-340-4395369 | 30.66 | 1.519 | 0.780 |  |
| ACM 3 | miR-340-4395369 | 30.31 | 1.519 | 0.780 |  |
| CTRL 1 | miR-340-4395369 | 28.12 | 1.000 | 1.000 |  |
| CTRL 2 | miR-340-4395369 | 32.00 | 1.000 | 1.000 |  |
| CTRL 3 | miR-340-4395369 | 32.98 | 1.000 | 1.000 |  |
| ACM 1 | miR-142-5p-4395359 | 29.47 | 3.018 | 0.787 |  |
| ACM 2 | miR-142-5p-4395359 | 28.02 | 3.018 | 0.787 |  |
| ACM 3 | miR-142-5p-4395359 | 29.33 | 3.018 | 0.787 |  |
| CTRL 1 | miR-142-5p-4395359 | 34.63 | 1.000 | 1.000 |  |
| CTRL 2 | miR-142-5p-4395359 | 34.45 | 1.000 | 1.000 |  |
| CTRL 3 | miR-142-5p-4395359 | 28.95 | 1.000 | 1.000 |  |
| ACM 1 | miR-99b-4373007 | 27.14 | 1.027 | 0.797 |  |
| ACM 2 | miR-99b-4373007 | 26.34 | 1.027 | 0.797 |  |
| ACM 3 | miR-99b-4373007 | 24.72 | 1.027 | 0.797 |  |
| CTRL 1 | miR-99b-4373007 | 28.85 | 1.000 | 1.000 |  |
| CTRL 2 | miR-99b-4373007 | 27.26 | 1.000 | 1.000 |  |
| CTRL 3 | miR-99b-4373007 | 27.52 | 1.000 | 1.000 |  |
| ACM 1 | miR-574-3p-4395460 | 28.78 | 1.256 | 0.803 |  |
| ACM 2 | miR-574-3p-4395460 | 27.05 | 1.256 | 0.803 |  |
| ACM 3 | miR-574-3p-4395460 | 28.21 | 1.256 | 0.803 |  |
| CTRL 1 | miR-574-3p-4395460 | 28.60 | 1.000 | 1.000 |  |
| CTRL 2 | miR-574-3p-4395460 | 30.78 | 1.000 | 1.000 |  |
| CTRL 3 | miR-574-3p-4395460 | 29.37 | 1.000 | 1.000 |  |
| ACM 1 | miR-660-4380925 | 27.36 | 1.244 | 0.812 |  |
| ACM 2 | miR-660-4380925 | 27.74 | 1.244 | 0.812 |  |
| ACM 3 | miR-660-4380925 | 26.72 | 1.244 | 0.812 |  |
| CTRL 1 | miR-660-4380925 | 30.02 | 1.000 | 1.000 |  |
| CTRL 2 | miR-660-4380925 | 30.14 | 1.000 | 1.000 |  |
| CTRL 3 | miR-660-4380925 | 26.32 | 1.000 | 1.000 |  |
| ACM 1 | let-7d-4395394 | 31.18 | 1.021 | 0.813 |  |
| ACM 2 | let-7d-4395394 | 29.01 | 1.021 | 0.813 |  |
| ACM 3 | let-7d-4395394 | 25.75 | 1.021 | 0.813 |  |
| CTRL 1 | let-7d-4395394 | 30.66 | 1.000 | 1.000 |  |
| CTRL 2 | let-7d-4395394 | 30.52 | 1.000 | 1.000 |  |
| CTRL 3 | let-7d-4395394 | 32.49 | 1.000 | 1.000 |  |
| ACM 1 | miR-324-5p-4373052 | 28.22 | 2.602 | 0.815 |  |
| ACM 2 | miR-324-5p-4373052 | 26.11 | 2.602 | 0.815 |  |
| ACM 3 | miR-324-5p-4373052 | 25.33 | 2.602 | 0.815 |  |
| CTRL 1 | miR-324-5p-4373052 | 31.37 | 1.000 | 1.000 |  |
| CTRL 2 | miR-324-5p-4373052 | 30.29 | 1.000 | 1.000 |  |
| CTRL 3 | miR-324-5p-4373052 | 25.87 | 1.000 | 1.000 |  |
| ACM 1 | miR-146a-4373132 | 24.07 | 1.082 | 0.830 |  |
| ACM 2 | miR-146a-4373132 | 22.93 | 1.082 | 0.830 |  |
| ACM 3 | miR-146a-4373132 | 22.42 | 1.082 | 0.830 |  |
| CTRL 1 | miR-146a-4373132 | 24.06 | 1.000 | 1.000 |  |
| CTRL 2 | miR-146a-4373132 | 25.34 | 1.000 | 1.000 |  |
| CTRL 3 | miR-146a-4373132 | 24.09 | 1.000 | 1.000 |  |
| ACM 1 | miR-874-4395379 | 30.11 | 0.570 | 0.866 |  |
| ACM 2 | miR-874-4395379 | 25.76 | 0.570 | 0.866 |  |
| ACM 3 | miR-874-4395379 | 28.71 | 0.570 | 0.866 |  |
| CTRL 1 | miR-874-4395379 | 29.93 | 1.000 | 1.000 |  |
| CTRL 2 | miR-874-4395379 | 29.19 | 1.000 | 1.000 |  |
| CTRL 3 | miR-874-4395379 | 26.76 | 1.000 | 1.000 |  |
| ACM 1 | miR-185-4395382 | 27.00 | 1.096 | 0.887 |  |
| ACM 2 | miR-185-4395382 | 27.35 | 1.096 | 0.887 |  |
| ACM 3 | miR-185-4395382 | 26.52 | 1.096 | 0.887 |  |
| CTRL 1 | miR-185-4395382 | 29.10 | 1.000 | 1.000 |  |
| CTRL 2 | miR-185-4395382 | 29.43 | 1.000 | 1.000 |  |
| CTRL 3 | miR-185-4395382 | 26.46 | 1.000 | 1.000 |  |
| ACM 1 | miR-22-4373079 | 28.18 | 0.875 | 0.895 |  |
| ACM 2 | miR-22-4373079 | 26.90 | 0.875 | 0.895 |  |
| ACM 3 | miR-22-4373079 | 24.71 | 0.875 | 0.895 |  |
| CTRL 1 | miR-22-4373079 | 28.46 | 1.000 | 1.000 |  |
| CTRL 2 | miR-22-4373079 | 28.00 | 1.000 | 1.000 |  |
| CTRL 3 | miR-22-4373079 | 26.40 | 1.000 | 1.000 |  |
| ACM 1 | miR-532-5p-4380928 | 26.91 | 1.333 | 0.904 |  |
| ACM 2 | miR-532-5p-4380928 | 26.67 | 1.333 | 0.904 |  |
| ACM 3 | miR-532-5p-4380928 | 25.96 | 1.333 | 0.904 |  |
| CTRL 1 | miR-532-5p-4380928 | 29.15 | 1.000 | 1.000 |  |
| CTRL 2 | miR-532-5p-4380928 | 29.52 | 1.000 | 1.000 |  |
| CTRL 3 | miR-532-5p-4380928 | 25.84 | 1.000 | 1.000 |  |
| ACM 1 | miR-93-4373302 | 25.13 | 1.484 | 0.908 |  |
| ACM 2 | miR-93-4373302 | 25.07 | 1.484 | 0.908 |  |
| ACM 3 | miR-93-4373302 | 24.19 | 1.484 | 0.908 |  |
| CTRL 1 | miR-93-4373302 | 28.37 | 1.000 | 1.000 |  |
| CTRL 2 | miR-93-4373302 | 27.39 | 1.000 | 1.000 |  |
| CTRL 3 | miR-93-4373302 | 24.06 | 1.000 | 1.000 |  |
| ACM 1 | miR-19a-4373099 | 25.97 | 1.008 | 0.909 |  |
| ACM 2 | miR-19a-4373099 | 27.50 | 1.008 | 0.909 |  |
| ACM 3 | miR-19a-4373099 | 25.92 | 1.008 | 0.909 |  |
| CTRL 1 | miR-19a-4373099 | 29.35 | 1.000 | 1.000 |  |
| CTRL 2 | miR-19a-4373099 | 27.87 | 1.000 | 1.000 |  |
| CTRL 3 | miR-19a-4373099 | 25.93 | 1.000 | 1.000 |  |
| ACM 1 | miR-502-3p-4395194 | 31.43 | 1.034 | 0.921 |  |
| ACM 2 | miR-502-3p-4395194 | 32.66 | 1.034 | 0.921 |  |
| ACM 3 | miR-502-3p-4395194 | 29.90 | 1.034 | 0.921 |  |
| CTRL 1 | miR-502-3p-4395194 | 33.18 | 1.000 | 1.000 |  |
| CTRL 2 | miR-502-3p-4395194 | 33.93 | 1.000 | 1.000 |  |
| CTRL 3 | miR-502-3p-4395194 | 30.76 | 1.000 | 1.000 |  |
| ACM 1 | miR-25-4373071 | 24.45 | 1.576 | 0.935 |  |
| ACM 2 | miR-25-4373071 | 24.25 | 1.576 | 0.935 |  |
| ACM 3 | miR-25-4373071 | 23.16 | 1.576 | 0.935 |  |
| CTRL 1 | miR-25-4373071 | 27.71 | 1.000 | 1.000 |  |
| CTRL 2 | miR-25-4373071 | 26.63 | 1.000 | 1.000 |  |
| CTRL 3 | miR-25-4373071 | 23.20 | 1.000 | 1.000 |  |
| ACM 1 | miR-140-3p-4395345 | 28.40 | 1.030 | 0.942 |  |
| ACM 2 | miR-140-3p-4395345 | 29.55 | 1.030 | 0.942 |  |
| ACM 3 | miR-140-3p-4395345 | 29.04 | 1.030 | 0.942 |  |
| CTRL 1 | miR-140-3p-4395345 | 29.92 | 1.000 | 1.000 |  |
| CTRL 2 | miR-140-3p-4395345 | 32.20 | 1.000 | 1.000 |  |
| CTRL 3 | miR-140-3p-4395345 | 28.71 | 1.000 | 1.000 |  |
| ACM 1 | miR-20a-4373286 | 24.05 | 1.180 | 0.955 |  |
| ACM 2 | miR-20a-4373286 | 24.32 | 1.180 | 0.955 |  |
| ACM 3 | miR-20a-4373286 | 20.38 | 1.180 | 0.955 |  |
| CTRL 1 | miR-20a-4373286 | 26.60 | 1.000 | 1.000 |  |
| CTRL 2 | miR-20a-4373286 | 26.19 | 1.000 | 1.000 |  |
| CTRL 3 | miR-20a-4373286 | 23.78 | 1.000 | 1.000 |  |
| ACM 1 | miR-92a-4395169 | 20.65 | 1.127 | 0.962 |  |
| ACM 2 | miR-92a-4395169 | 20.95 | 1.127 | 0.962 |  |
| ACM 3 | miR-92a-4395169 | 19.73 | 1.127 | 0.962 |  |
| CTRL 1 | miR-92a-4395169 | 22.80 | 1.000 | 1.000 |  |
| CTRL 2 | miR-92a-4395169 | 22.73 | 1.000 | 1.000 |  |
| CTRL 3 | miR-92a-4395169 | 20.04 | 1.000 | 1.000 |  |
| ACM 1 | miR-335-4373045 | 33.08 | 0.944 | 0.967 |  |
| ACM 2 | miR-335-4373045 | 30.40 | 0.944 | 0.967 |  |
| ACM 3 | miR-335-4373045 | 29.49 | 0.944 | 0.967 |  |
| CTRL 1 | miR-335-4373045 | 29.45 | 1.000 | 1.000 |  |
| CTRL 2 | miR-335-4373045 | 33.67 | 1.000 | 1.000 |  |
| CTRL 3 | miR-335-4373045 | 30.47 | 1.000 | 1.000 |  |
| ACM 1 | miR-451-4373360 | 19.50 | 2.002 | 0.977 |  |
| ACM 2 | miR-451-4373360 | 19.90 | 2.002 | 0.977 |  |
| ACM 3 | miR-451-4373360 | 18.31 | 2.002 | 0.977 |  |
| CTRL 1 | miR-451-4373360 | 23.35 | 1.000 | 1.000 |  |
| CTRL 2 | miR-451-4373360 | 22.64 | 1.000 | 1.000 |  |
| CTRL 3 | miR-451-4373360 | 18.44 | 1.000 | 1.000 |  |
| ACM 1 | miR-95-4373011 | 32.14 | 0.441 | 0.986 |  |
| ACM 2 | miR-95-4373011 | 34.64 | 0.441 | 0.986 |  |
| ACM 3 | miR-95-4373011 | 30.23 | 0.441 | 0.986 |  |
| CTRL 1 | miR-95-4373011 | 33.10 | 1.000 | 1.000 |  |
| CTRL 2 | miR-95-4373011 | 32.95 | 1.000 | 1.000 |  |
| CTRL 3 | miR-95-4373011 | 33.56 | 1.000 | 1.000 |  |
| ACM 1 | let-7a-4373169 | 30.87 | 1.458 | 0.600 |  |
| ACM 2 | let-7a-4373169 | 27.32 | 1.458 | 0.600 |  |
| ACM 3 | let-7a-4373169 | 28.10 | 1.458 | 0.600 |  |
| CTRL 1 | let-7a-4373169 | 28.60 | 1.000 | 1.000 |  |
| CTRL 2 | let-7a-4373169 | 32.66 | 1.000 | 1.000 |  |
| CTRL 3 | let-7a-4373169 | 25.91 | 1.000 | 1.000 |  |
| ACM 1 | let-7b-4395446 | 23.67 | 3.156 | 0.956 |  |
| ACM 2 | let-7b-4395446 | 23.58 | 3.156 | 0.956 |  |
| ACM 3 | let-7b-4395446 | 21.05 | 3.156 | 0.956 |  |
| CTRL 1 | let-7b-4395446 | 22.08 | 1.000 | 1.000 |  |
| CTRL 2 | let-7b-4395446 | 21.35 | 1.000 | 1.000 |  |
| CTRL 3 | let-7b-4395446 | 23.53 | 1.000 | 1.000 |  |
| ACM 1 | miR-502-5p-4373227 | 25.19 | 0.575 | 0.798 |  |
| ACM 2 | miR-502-5p-4373227 | 32.75 | 0.575 | 0.798 |  |
| ACM 3 | miR-502-5p-4373227 | 29.77 | 0.575 | 0.798 |  |
| CTRL 1 | miR-502-5p-4373227 | 14.75 | 1.000 | 1.000 |  |
| CTRL 2 | miR-502-5p-4373227 | 33.65 | 1.000 | 1.000 |  |
| CTRL 3 | miR-502-5p-4373227 | 32.95 | 1.000 | 1.000 |  |
| ACM 1 | miR-100-4373160 | 29.41 | 4.318 | 0.469 |  |
| ACM 2 | miR-100-4373160 | 29.74 | 4.318 | 0.469 |  |
| ACM 3 | miR-100-4373160 | 33.84 | 4.318 | 0.469 |  |
| CTRL 1 | miR-100-4373160 | 34.79 | 1.000 | 1.000 |  |
| CTRL 2 | miR-100-4373160 | 33.84 | 1.000 | 1.000 |  |
| CTRL 3 | miR-100-4373160 | 32.13 | 1.000 | 1.000 |  |
| ACM 1 | miR-182-4395445 | Undetermined |  |  |  |
| ACM 2 | miR-182-4395445 | Undetermined |  |  |  |
| ACM 3 | miR-182-4395445 | Undetermined |  |  |  |
| CTRL 1 | miR-182-4395445 | Undetermined |  |  |  |
| CTRL 2 | miR-182-4395445 | Undetermined |  |  |  |
| CTRL 3 | miR-182-4395445 | Undetermined |  |  |  |
| ACM 1 | miR-202-4395474 | Undetermined |  |  |  |
| ACM 2 | miR-202-4395474 | Undetermined |  |  |  |
| ACM 3 | miR-202-4395474 | Undetermined |  |  |  |
| CTRL 1 | miR-202-4395474 | Undetermined |  |  |  |
| CTRL 2 | miR-202-4395474 | Undetermined |  |  |  |
| CTRL 3 | miR-202-4395474 | Undetermined |  |  |  |
| ACM 1 | let-7e-4395517 | Undetermined |  |  |  |
| ACM 2 | let-7e-4395517 | Undetermined |  |  |  |
| ACM 3 | let-7e-4395517 | Undetermined |  |  |  |
| CTRL 1 | let-7e-4395517 | Undetermined |  |  |  |
| CTRL 2 | let-7e-4395517 | Undetermined |  |  |  |
| CTRL 3 | let-7e-4395517 | Undetermined |  |  |  |
| ACM 1 | miR-10b-4395329 | Undetermined |  |  |  |
| ACM 2 | miR-10b-4395329 | Undetermined |  |  |  |
| ACM 3 | miR-10b-4395329 | Undetermined |  |  |  |
| CTRL 1 | miR-10b-4395329 | Undetermined |  |  |  |
| CTRL 2 | miR-10b-4395329 | Undetermined |  |  |  |
| CTRL 3 | miR-10b-4395329 | Undetermined |  |  |  |
| ACM 1 | ath-miR159a-4373390 | Undetermined |  |  |  |
| ACM 2 | ath-miR159a-4373390 | Undetermined |  |  |  |
| ACM 3 | ath-miR159a-4373390 | Undetermined |  |  |  |
| CTRL 1 | ath-miR159a-4373390 | Undetermined |  |  |  |
| CTRL 2 | ath-miR159a-4373390 | Undetermined |  |  |  |
| CTRL 3 | ath-miR159a-4373390 | Undetermined |  |  |  |
| ACM 1 | miR-105-4395278 | Undetermined |  |  |  |
| ACM 2 | miR-105-4395278 | Undetermined |  |  |  |
| ACM 3 | miR-105-4395278 | Undetermined |  |  |  |
| CTRL 1 | miR-105-4395278 | Undetermined |  |  |  |
| CTRL 2 | miR-105-4395278 | Undetermined |  |  |  |
| CTRL 3 | miR-105-4395278 | Undetermined |  |  |  |
| ACM 1 | miR-107-4373154 | Undetermined |  |  |  |
| ACM 2 | miR-107-4373154 | Undetermined |  |  |  |
| ACM 3 | miR-107-4373154 | Undetermined |  |  |  |
| CTRL 1 | miR-107-4373154 | Undetermined |  |  |  |
| CTRL 2 | miR-107-4373154 | Undetermined |  |  |  |
| CTRL 3 | miR-107-4373154 | Undetermined |  |  |  |
| ACM 1 | let-7f-4373164 | Undetermined |  |  |  |
| ACM 2 | let-7f-4373164 | Undetermined |  |  |  |
| ACM 3 | let-7f-4373164 | Undetermined |  |  |  |
| CTRL 1 | let-7f-4373164 | Undetermined |  |  |  |
| CTRL 2 | let-7f-4373164 | Undetermined |  |  |  |
| CTRL 3 | let-7f-4373164 | Undetermined |  |  |  |
| ACM 1 | miR-124-4373295 | Undetermined |  |  |  |
| ACM 2 | miR-124-4373295 | Undetermined |  |  |  |
| ACM 3 | miR-124-4373295 | Undetermined |  |  |  |
| CTRL 1 | miR-124-4373295 | Undetermined |  |  |  |
| CTRL 2 | miR-124-4373295 | Undetermined |  |  |  |
| CTRL 3 | miR-124-4373295 | Undetermined |  |  |  |
| ACM 1 | miR-125a-3p-4395310 | Undetermined |  |  |  |
| ACM 2 | miR-125a-3p-4395310 | Undetermined |  |  |  |
| ACM 3 | miR-125a-3p-4395310 | Undetermined |  |  |  |
| CTRL 1 | miR-125a-3p-4395310 | Undetermined |  |  |  |
| CTRL 2 | miR-125a-3p-4395310 | Undetermined |  |  |  |
| CTRL 3 | miR-125a-3p-4395310 | Undetermined |  |  |  |
| ACM 1 | miR-127-3p-4373147 | Undetermined |  |  |  |
| ACM 2 | miR-127-3p-4373147 | Undetermined |  |  |  |
| ACM 3 | miR-127-3p-4373147 | Undetermined |  |  |  |
| CTRL 1 | miR-127-3p-4373147 | Undetermined |  |  |  |
| CTRL 2 | miR-127-3p-4373147 | Undetermined |  |  |  |
| CTRL 3 | miR-127-3p-4373147 | Undetermined |  |  |  |
| ACM 1 | miR-127-5p-4395340 | Undetermined |  |  |  |
| ACM 2 | miR-127-5p-4395340 | Undetermined |  |  |  |
| ACM 3 | miR-127-5p-4395340 | Undetermined |  |  |  |
| CTRL 1 | miR-127-5p-4395340 | Undetermined |  |  |  |
| CTRL 2 | miR-127-5p-4395340 | Undetermined |  |  |  |
| CTRL 3 | miR-127-5p-4395340 | Undetermined |  |  |  |
| ACM 1 | miR-129-3p-4373297 | Undetermined |  |  |  |
| ACM 2 | miR-129-3p-4373297 | Undetermined |  |  |  |
| ACM 3 | miR-129-3p-4373297 | Undetermined |  |  |  |
| CTRL 1 | miR-129-3p-4373297 | Undetermined |  |  |  |
| CTRL 2 | miR-129-3p-4373297 | Undetermined |  |  |  |
| CTRL 3 | miR-129-3p-4373297 | Undetermined |  |  |  |
| ACM 1 | miR-129-5p-4373171 | Undetermined |  |  |  |
| ACM 2 | miR-129-5p-4373171 | Undetermined |  |  |  |
| ACM 3 | miR-129-5p-4373171 | Undetermined |  |  |  |
| CTRL 1 | miR-129-5p-4373171 | Undetermined |  |  |  |
| CTRL 2 | miR-129-5p-4373171 | Undetermined |  |  |  |
| CTRL 3 | miR-129-5p-4373171 | Undetermined |  |  |  |
| ACM 1 | miR-130a-4373145 | Undetermined |  |  |  |
| ACM 2 | miR-130a-4373145 | Undetermined |  |  |  |
| ACM 3 | miR-130a-4373145 | Undetermined |  |  |  |
| CTRL 1 | miR-130a-4373145 | Undetermined |  |  |  |
| CTRL 2 | miR-130a-4373145 | Undetermined |  |  |  |
| CTRL 3 | miR-130a-4373145 | Undetermined |  |  |  |
| ACM 1 | miR-133b-4395358 | Undetermined |  |  |  |
| ACM 2 | miR-133b-4395358 | Undetermined |  |  |  |
| ACM 3 | miR-133b-4395358 | Undetermined |  |  |  |
| CTRL 1 | miR-133b-4395358 | Undetermined |  |  |  |
| CTRL 2 | miR-133b-4395358 | Undetermined |  |  |  |
| CTRL 3 | miR-133b-4395358 | Undetermined |  |  |  |
| ACM 1 | miR-135a-4373140 | Undetermined |  |  |  |
| ACM 2 | miR-135a-4373140 | Undetermined |  |  |  |
| ACM 3 | miR-135a-4373140 | Undetermined |  |  |  |
| CTRL 1 | miR-135a-4373140 | Undetermined |  |  |  |
| CTRL 2 | miR-135a-4373140 | Undetermined |  |  |  |
| CTRL 3 | miR-135a-4373140 | Undetermined |  |  |  |
| ACM 1 | miR-135b-4395372 | Undetermined |  |  |  |
| ACM 2 | miR-135b-4395372 | Undetermined |  |  |  |
| ACM 3 | miR-135b-4395372 | Undetermined |  |  |  |
| CTRL 1 | miR-135b-4395372 | Undetermined |  |  |  |
| CTRL 2 | miR-135b-4395372 | Undetermined |  |  |  |
| CTRL 3 | miR-135b-4395372 | Undetermined |  |  |  |
| ACM 1 | miR-136-4373173 | Undetermined |  |  |  |
| ACM 2 | miR-136-4373173 | Undetermined |  |  |  |
| ACM 3 | miR-136-4373173 | Undetermined |  |  |  |
| CTRL 1 | miR-136-4373173 | Undetermined |  |  |  |
| CTRL 2 | miR-136-4373173 | Undetermined |  |  |  |
| CTRL 3 | miR-136-4373173 | Undetermined |  |  |  |
| ACM 1 | miR-137-4373301 | Undetermined |  |  |  |
| ACM 2 | miR-137-4373301 | Undetermined |  |  |  |
| ACM 3 | miR-137-4373301 | Undetermined |  |  |  |
| CTRL 1 | miR-137-4373301 | Undetermined |  |  |  |
| CTRL 2 | miR-137-4373301 | Undetermined |  |  |  |
| CTRL 3 | miR-137-4373301 | Undetermined |  |  |  |
| ACM 1 | miR-138-4395395 | Undetermined |  |  |  |
| ACM 2 | miR-138-4395395 | Undetermined |  |  |  |
| ACM 3 | miR-138-4395395 | Undetermined |  |  |  |
| CTRL 1 | miR-138-4395395 | Undetermined |  |  |  |
| CTRL 2 | miR-138-4395395 | Undetermined |  |  |  |
| CTRL 3 | miR-138-4395395 | Undetermined |  |  |  |
| ACM 1 | miR-1-4395333 | Undetermined |  |  |  |
| ACM 2 | miR-1-4395333 | Undetermined |  |  |  |
| ACM 3 | miR-1-4395333 | Undetermined |  |  |  |
| CTRL 1 | miR-1-4395333 | Undetermined |  |  |  |
| CTRL 2 | miR-1-4395333 | Undetermined |  |  |  |
| CTRL 3 | miR-1-4395333 | Undetermined |  |  |  |
| ACM 1 | miR-146b-3p-4395472 | Undetermined |  |  |  |
| ACM 2 | miR-146b-3p-4395472 | Undetermined |  |  |  |
| ACM 3 | miR-146b-3p-4395472 | Undetermined |  |  |  |
| CTRL 1 | miR-146b-3p-4395472 | Undetermined |  |  |  |
| CTRL 2 | miR-146b-3p-4395472 | Undetermined |  |  |  |
| CTRL 3 | miR-146b-3p-4395472 | Undetermined |  |  |  |
| ACM 1 | miR-147-4373131 | Undetermined |  |  |  |
| ACM 2 | miR-147-4373131 | Undetermined |  |  |  |
| ACM 3 | miR-147-4373131 | Undetermined |  |  |  |
| CTRL 1 | miR-147-4373131 | Undetermined |  |  |  |
| CTRL 2 | miR-147-4373131 | Undetermined |  |  |  |
| CTRL 3 | miR-147-4373131 | Undetermined |  |  |  |
| ACM 1 | miR-147b-4395373 | Undetermined |  |  |  |
| ACM 2 | miR-147b-4395373 | Undetermined |  |  |  |
| ACM 3 | miR-147b-4395373 | Undetermined |  |  |  |
| CTRL 1 | miR-147b-4395373 | Undetermined |  |  |  |
| CTRL 2 | miR-147b-4395373 | Undetermined |  |  |  |
| CTRL 3 | miR-147b-4395373 | Undetermined |  |  |  |
| ACM 1 | miR-148a-4373130 | Undetermined |  |  |  |
| ACM 2 | miR-148a-4373130 | Undetermined |  |  |  |
| ACM 3 | miR-148a-4373130 | Undetermined |  |  |  |
| CTRL 1 | miR-148a-4373130 | Undetermined |  |  |  |
| CTRL 2 | miR-148a-4373130 | Undetermined |  |  |  |
| CTRL 3 | miR-148a-4373130 | Undetermined |  |  |  |
| ACM 1 | miR-148b-4373129 | Undetermined |  |  |  |
| ACM 2 | miR-148b-4373129 | Undetermined |  |  |  |
| ACM 3 | miR-148b-4373129 | Undetermined |  |  |  |
| CTRL 1 | miR-148b-4373129 | Undetermined |  |  |  |
| CTRL 2 | miR-148b-4373129 | Undetermined |  |  |  |
| CTRL 3 | miR-148b-4373129 | Undetermined |  |  |  |
| ACM 1 | miR-149-4395366 | Undetermined |  |  |  |
| ACM 2 | miR-149-4395366 | Undetermined |  |  |  |
| ACM 3 | miR-149-4395366 | Undetermined |  |  |  |
| CTRL 1 | miR-149-4395366 | Undetermined |  |  |  |
| CTRL 2 | miR-149-4395366 | Undetermined |  |  |  |
| CTRL 3 | miR-149-4395366 | Undetermined |  |  |  |
| ACM 1 | miR-153-4373305 | Undetermined |  |  |  |
| ACM 2 | miR-153-4373305 | Undetermined |  |  |  |
| ACM 3 | miR-153-4373305 | Undetermined |  |  |  |
| CTRL 1 | miR-153-4373305 | Undetermined |  |  |  |
| CTRL 2 | miR-153-4373305 | Undetermined |  |  |  |
| CTRL 3 | miR-153-4373305 | Undetermined |  |  |  |
| ACM 1 | miR-154-4373270 | Undetermined |  |  |  |
| ACM 2 | miR-154-4373270 | Undetermined |  |  |  |
| ACM 3 | miR-154-4373270 | Undetermined |  |  |  |
| CTRL 1 | miR-154-4373270 | Undetermined |  |  |  |
| CTRL 2 | miR-154-4373270 | Undetermined |  |  |  |
| CTRL 3 | miR-154-4373270 | Undetermined |  |  |  |
| ACM 1 | miR-15a-4373123 | Undetermined |  |  |  |
| ACM 2 | miR-15a-4373123 | Undetermined |  |  |  |
| ACM 3 | miR-15a-4373123 | Undetermined |  |  |  |
| CTRL 1 | miR-15a-4373123 | Undetermined |  |  |  |
| CTRL 2 | miR-15a-4373123 | Undetermined |  |  |  |
| CTRL 3 | miR-15a-4373123 | Undetermined |  |  |  |
| ACM 1 | miR-181c-4373115 | Undetermined |  |  |  |
| ACM 2 | miR-181c-4373115 | Undetermined |  |  |  |
| ACM 3 | miR-181c-4373115 | Undetermined |  |  |  |
| CTRL 1 | miR-181c-4373115 | Undetermined |  |  |  |
| CTRL 2 | miR-181c-4373115 | Undetermined |  |  |  |
| CTRL 3 | miR-181c-4373115 | Undetermined |  |  |  |
| ACM 1 | miR-183-4395380 | Undetermined |  |  |  |
| ACM 2 | miR-183-4395380 | Undetermined |  |  |  |
| ACM 3 | miR-183-4395380 | Undetermined |  |  |  |
| CTRL 1 | miR-183-4395380 | Undetermined |  |  |  |
| CTRL 2 | miR-183-4395380 | Undetermined |  |  |  |
| CTRL 3 | miR-183-4395380 | Undetermined |  |  |  |
| ACM 1 | miR-187-4373307 | Undetermined |  |  |  |
| ACM 2 | miR-187-4373307 | Undetermined |  |  |  |
| ACM 3 | miR-187-4373307 | Undetermined |  |  |  |
| CTRL 1 | miR-187-4373307 | Undetermined |  |  |  |
| CTRL 2 | miR-187-4373307 | Undetermined |  |  |  |
| CTRL 3 | miR-187-4373307 | Undetermined |  |  |  |
| ACM 1 | miR-188-3p-4395217 | Undetermined |  |  |  |
| ACM 2 | miR-188-3p-4395217 | Undetermined |  |  |  |
| ACM 3 | miR-188-3p-4395217 | Undetermined |  |  |  |
| CTRL 1 | miR-188-3p-4395217 | Undetermined |  |  |  |
| CTRL 2 | miR-188-3p-4395217 | Undetermined |  |  |  |
| CTRL 3 | miR-188-3p-4395217 | Undetermined |  |  |  |
| ACM 1 | miR-18b-4395328 | Undetermined |  |  |  |
| ACM 2 | miR-18b-4395328 | Undetermined |  |  |  |
| ACM 3 | miR-18b-4395328 | Undetermined |  |  |  |
| CTRL 1 | miR-18b-4395328 | Undetermined |  |  |  |
| CTRL 2 | miR-18b-4395328 | Undetermined |  |  |  |
| CTRL 3 | miR-18b-4395328 | Undetermined |  |  |  |
| ACM 1 | miR-190-4373110 | Undetermined |  |  |  |
| ACM 2 | miR-190-4373110 | Undetermined |  |  |  |
| ACM 3 | miR-190-4373110 | Undetermined |  |  |  |
| CTRL 1 | miR-190-4373110 | Undetermined |  |  |  |
| CTRL 2 | miR-190-4373110 | Undetermined |  |  |  |
| CTRL 3 | miR-190-4373110 | Undetermined |  |  |  |
| ACM 1 | miR-193a-3p-4395361 | Undetermined |  |  |  |
| ACM 2 | miR-193a-3p-4395361 | Undetermined |  |  |  |
| ACM 3 | miR-193a-3p-4395361 | Undetermined |  |  |  |
| CTRL 1 | miR-193a-3p-4395361 | Undetermined |  |  |  |
| CTRL 2 | miR-193a-3p-4395361 | Undetermined |  |  |  |
| CTRL 3 | miR-193a-3p-4395361 | Undetermined |  |  |  |
| ACM 1 | miR-196b-4395326 | Undetermined |  |  |  |
| ACM 2 | miR-196b-4395326 | Undetermined |  |  |  |
| ACM 3 | miR-196b-4395326 | Undetermined |  |  |  |
| CTRL 1 | miR-196b-4395326 | Undetermined |  |  |  |
| CTRL 2 | miR-196b-4395326 | Undetermined |  |  |  |
| CTRL 3 | miR-196b-4395326 | Undetermined |  |  |  |
| ACM 1 | miR-197-4373102 | Undetermined |  |  |  |
| ACM 2 | miR-197-4373102 | Undetermined |  |  |  |
| ACM 3 | miR-197-4373102 | Undetermined |  |  |  |
| CTRL 1 | miR-197-4373102 | Undetermined |  |  |  |
| CTRL 2 | miR-197-4373102 | Undetermined |  |  |  |
| CTRL 3 | miR-197-4373102 | Undetermined |  |  |  |
| ACM 1 | miR-198-4395384 | Undetermined |  |  |  |
| ACM 2 | miR-198-4395384 | Undetermined |  |  |  |
| ACM 3 | miR-198-4395384 | Undetermined |  |  |  |
| CTRL 1 | miR-198-4395384 | Undetermined |  |  |  |
| CTRL 2 | miR-198-4395384 | Undetermined |  |  |  |
| CTRL 3 | miR-198-4395384 | Undetermined |  |  |  |
| ACM 1 | miR-199a-3p-4395415 | Undetermined |  |  |  |
| ACM 2 | miR-199a-3p-4395415 | Undetermined |  |  |  |
| ACM 3 | miR-199a-3p-4395415 | Undetermined |  |  |  |
| CTRL 1 | miR-199a-3p-4395415 | Undetermined |  |  |  |
| CTRL 2 | miR-199a-3p-4395415 | Undetermined |  |  |  |
| CTRL 3 | miR-199a-3p-4395415 | Undetermined |  |  |  |
| ACM 1 | miR-199a-5p-4373272 | Undetermined |  |  |  |
| ACM 2 | miR-199a-5p-4373272 | Undetermined |  |  |  |
| ACM 3 | miR-199a-5p-4373272 | Undetermined |  |  |  |
| CTRL 1 | miR-199a-5p-4373272 | Undetermined |  |  |  |
| CTRL 2 | miR-199a-5p-4373272 | Undetermined |  |  |  |
| CTRL 3 | miR-199a-5p-4373272 | Undetermined |  |  |  |
| ACM 1 | miR-199b-5p-4373100 | Undetermined |  |  |  |
| ACM 2 | miR-199b-5p-4373100 | Undetermined |  |  |  |
| ACM 3 | miR-199b-5p-4373100 | Undetermined |  |  |  |
| CTRL 1 | miR-199b-5p-4373100 | Undetermined |  |  |  |
| CTRL 2 | miR-199b-5p-4373100 | Undetermined |  |  |  |
| CTRL 3 | miR-199b-5p-4373100 | Undetermined |  |  |  |
| ACM 1 | miR-200b-4395362 | Undetermined |  |  |  |
| ACM 2 | miR-200b-4395362 | Undetermined |  |  |  |
| ACM 3 | miR-200b-4395362 | Undetermined |  |  |  |
| CTRL 1 | miR-200b-4395362 | Undetermined |  |  |  |
| CTRL 2 | miR-200b-4395362 | Undetermined |  |  |  |
| CTRL 3 | miR-200b-4395362 | Undetermined |  |  |  |
| ACM 1 | miR-200c-4395411 | Undetermined |  |  |  |
| ACM 2 | miR-200c-4395411 | Undetermined |  |  |  |
| ACM 3 | miR-200c-4395411 | Undetermined |  |  |  |
| CTRL 1 | miR-200c-4395411 | Undetermined |  |  |  |
| CTRL 2 | miR-200c-4395411 | Undetermined |  |  |  |
| CTRL 3 | miR-200c-4395411 | Undetermined |  |  |  |
| ACM 1 | miR-203-4373095 | Undetermined |  |  |  |
| ACM 2 | miR-203-4373095 | Undetermined |  |  |  |
| ACM 3 | miR-203-4373095 | Undetermined |  |  |  |
| CTRL 1 | miR-203-4373095 | Undetermined |  |  |  |
| CTRL 2 | miR-203-4373095 | Undetermined |  |  |  |
| CTRL 3 | miR-203-4373095 | Undetermined |  |  |  |
| ACM 1 | miR-208-4373091 | Undetermined |  |  |  |
| ACM 2 | miR-208-4373091 | Undetermined |  |  |  |
| ACM 3 | miR-208-4373091 | Undetermined |  |  |  |
| CTRL 1 | miR-208-4373091 | Undetermined |  |  |  |
| CTRL 2 | miR-208-4373091 | Undetermined |  |  |  |
| CTRL 3 | miR-208-4373091 | Undetermined |  |  |  |
| ACM 1 | miR-208b-4395401 | Undetermined |  |  |  |
| ACM 2 | miR-208b-4395401 | Undetermined |  |  |  |
| ACM 3 | miR-208b-4395401 | Undetermined |  |  |  |
| CTRL 1 | miR-208b-4395401 | Undetermined |  |  |  |
| CTRL 2 | miR-208b-4395401 | Undetermined |  |  |  |
| CTRL 3 | miR-208b-4395401 | Undetermined |  |  |  |
| ACM 1 | miR-211-4373088 | Undetermined |  |  |  |
| ACM 2 | miR-211-4373088 | Undetermined |  |  |  |
| ACM 3 | miR-211-4373088 | Undetermined |  |  |  |
| CTRL 1 | miR-211-4373088 | Undetermined |  |  |  |
| CTRL 2 | miR-211-4373088 | Undetermined |  |  |  |
| CTRL 3 | miR-211-4373088 | Undetermined |  |  |  |
| ACM 1 | miR-212-4373087 | Undetermined |  |  |  |
| ACM 2 | miR-212-4373087 | Undetermined |  |  |  |
| ACM 3 | miR-212-4373087 | Undetermined |  |  |  |
| CTRL 1 | miR-212-4373087 | Undetermined |  |  |  |
| CTRL 2 | miR-212-4373087 | Undetermined |  |  |  |
| CTRL 3 | miR-212-4373087 | Undetermined |  |  |  |
| ACM 1 | miR-215-4373084 | Undetermined |  |  |  |
| ACM 2 | miR-215-4373084 | Undetermined |  |  |  |
| ACM 3 | miR-215-4373084 | Undetermined |  |  |  |
| CTRL 1 | miR-215-4373084 | Undetermined |  |  |  |
| CTRL 2 | miR-215-4373084 | Undetermined |  |  |  |
| CTRL 3 | miR-215-4373084 | Undetermined |  |  |  |
| ACM 1 | miR-216a-4395331 | Undetermined |  |  |  |
| ACM 2 | miR-216a-4395331 | Undetermined |  |  |  |
| ACM 3 | miR-216a-4395331 | Undetermined |  |  |  |
| CTRL 1 | miR-216a-4395331 | Undetermined |  |  |  |
| CTRL 2 | miR-216a-4395331 | Undetermined |  |  |  |
| CTRL 3 | miR-216a-4395331 | Undetermined |  |  |  |
| ACM 1 | miR-216b-4395437 | Undetermined |  |  |  |
| ACM 2 | miR-216b-4395437 | Undetermined |  |  |  |
| ACM 3 | miR-216b-4395437 | Undetermined |  |  |  |
| CTRL 1 | miR-216b-4395437 | Undetermined |  |  |  |
| CTRL 2 | miR-216b-4395437 | Undetermined |  |  |  |
| CTRL 3 | miR-216b-4395437 | Undetermined |  |  |  |
| ACM 1 | miR-217-4395448 | Undetermined |  |  |  |
| ACM 2 | miR-217-4395448 | Undetermined |  |  |  |
| ACM 3 | miR-217-4395448 | Undetermined |  |  |  |
| CTRL 1 | miR-217-4395448 | Undetermined |  |  |  |
| CTRL 2 | miR-217-4395448 | Undetermined |  |  |  |
| CTRL 3 | miR-217-4395448 | Undetermined |  |  |  |
| ACM 1 | miR-219-1-3p-4395206 | Undetermined |  |  |  |
| ACM 2 | miR-219-1-3p-4395206 | Undetermined |  |  |  |
| ACM 3 | miR-219-1-3p-4395206 | Undetermined |  |  |  |
| CTRL 1 | miR-219-1-3p-4395206 | Undetermined |  |  |  |
| CTRL 2 | miR-219-1-3p-4395206 | Undetermined |  |  |  |
| CTRL 3 | miR-219-1-3p-4395206 | Undetermined |  |  |  |
| ACM 1 | miR-219-2-3p-4395501 | Undetermined |  |  |  |
| ACM 2 | miR-219-2-3p-4395501 | Undetermined |  |  |  |
| ACM 3 | miR-219-2-3p-4395501 | Undetermined |  |  |  |
| CTRL 1 | miR-219-2-3p-4395501 | Undetermined |  |  |  |
| CTRL 2 | miR-219-2-3p-4395501 | Undetermined |  |  |  |
| CTRL 3 | miR-219-2-3p-4395501 | Undetermined |  |  |  |
| ACM 1 | miR-219-5p-4373080 | Undetermined |  |  |  |
| ACM 2 | miR-219-5p-4373080 | Undetermined |  |  |  |
| ACM 3 | miR-219-5p-4373080 | Undetermined |  |  |  |
| CTRL 1 | miR-219-5p-4373080 | Undetermined |  |  |  |
| CTRL 2 | miR-219-5p-4373080 | Undetermined |  |  |  |
| CTRL 3 | miR-219-5p-4373080 | Undetermined |  |  |  |
| ACM 1 | miR-220-4373078 | Undetermined |  |  |  |
| ACM 2 | miR-220-4373078 | Undetermined |  |  |  |
| ACM 3 | miR-220-4373078 | Undetermined |  |  |  |
| CTRL 1 | miR-220-4373078 | Undetermined |  |  |  |
| CTRL 2 | miR-220-4373078 | Undetermined |  |  |  |
| CTRL 3 | miR-220-4373078 | Undetermined |  |  |  |
| ACM 1 | miR-220b-4395317 | Undetermined |  |  |  |
| ACM 2 | miR-220b-4395317 | Undetermined |  |  |  |
| ACM 3 | miR-220b-4395317 | Undetermined |  |  |  |
| CTRL 1 | miR-220b-4395317 | Undetermined |  |  |  |
| CTRL 2 | miR-220b-4395317 | Undetermined |  |  |  |
| CTRL 3 | miR-220b-4395317 | Undetermined |  |  |  |
| ACM 1 | miR-220c-4395322 | Undetermined |  |  |  |
| ACM 2 | miR-220c-4395322 | Undetermined |  |  |  |
| ACM 3 | miR-220c-4395322 | Undetermined |  |  |  |
| CTRL 1 | miR-220c-4395322 | Undetermined |  |  |  |
| CTRL 2 | miR-220c-4395322 | Undetermined |  |  |  |
| CTRL 3 | miR-220c-4395322 | Undetermined |  |  |  |
| ACM 1 | miR-224-4395210 | Undetermined |  |  |  |
| ACM 2 | miR-224-4395210 | Undetermined |  |  |  |
| ACM 3 | miR-224-4395210 | Undetermined |  |  |  |
| CTRL 1 | miR-224-4395210 | Undetermined |  |  |  |
| CTRL 2 | miR-224-4395210 | Undetermined |  |  |  |
| CTRL 3 | miR-224-4395210 | Undetermined |  |  |  |
| ACM 1 | miR-23a-4373074 | Undetermined |  |  |  |
| ACM 2 | miR-23a-4373074 | Undetermined |  |  |  |
| ACM 3 | miR-23a-4373074 | Undetermined |  |  |  |
| CTRL 1 | miR-23a-4373074 | Undetermined |  |  |  |
| CTRL 2 | miR-23a-4373074 | Undetermined |  |  |  |
| CTRL 3 | miR-23a-4373074 | Undetermined |  |  |  |
| ACM 1 | miR-23b-4373073 | Undetermined |  |  |  |
| ACM 2 | miR-23b-4373073 | Undetermined |  |  |  |
| ACM 3 | miR-23b-4373073 | Undetermined |  |  |  |
| CTRL 1 | miR-23b-4373073 | Undetermined |  |  |  |
| CTRL 2 | miR-23b-4373073 | Undetermined |  |  |  |
| CTRL 3 | miR-23b-4373073 | Undetermined |  |  |  |
| ACM 1 | miR-296-3p-4395212 | Undetermined |  |  |  |
| ACM 2 | miR-296-3p-4395212 | Undetermined |  |  |  |
| ACM 3 | miR-296-3p-4395212 | Undetermined |  |  |  |
| CTRL 1 | miR-296-3p-4395212 | Undetermined |  |  |  |
| CTRL 2 | miR-296-3p-4395212 | Undetermined |  |  |  |
| CTRL 3 | miR-296-3p-4395212 | Undetermined |  |  |  |
| ACM 1 | miR-298-4395301 | Undetermined |  |  |  |
| ACM 2 | miR-298-4395301 | Undetermined |  |  |  |
| ACM 3 | miR-298-4395301 | Undetermined |  |  |  |
| CTRL 1 | miR-298-4395301 | Undetermined |  |  |  |
| CTRL 2 | miR-298-4395301 | Undetermined |  |  |  |
| CTRL 3 | miR-298-4395301 | Undetermined |  |  |  |
| ACM 1 | miR-299-3p-4373189 | Undetermined |  |  |  |
| ACM 2 | miR-299-3p-4373189 | Undetermined |  |  |  |
| ACM 3 | miR-299-3p-4373189 | Undetermined |  |  |  |
| CTRL 1 | miR-299-3p-4373189 | Undetermined |  |  |  |
| CTRL 2 | miR-299-3p-4373189 | Undetermined |  |  |  |
| CTRL 3 | miR-299-3p-4373189 | Undetermined |  |  |  |
| ACM 1 | miR-299-5p-4373188 | Undetermined |  |  |  |
| ACM 2 | miR-299-5p-4373188 | Undetermined |  |  |  |
| ACM 3 | miR-299-5p-4373188 | Undetermined |  |  |  |
| CTRL 1 | miR-299-5p-4373188 | Undetermined |  |  |  |
| CTRL 2 | miR-299-5p-4373188 | Undetermined |  |  |  |
| CTRL 3 | miR-299-5p-4373188 | Undetermined |  |  |  |
| ACM 1 | miR-29b-4373288 | Undetermined |  |  |  |
| ACM 2 | miR-29b-4373288 | Undetermined |  |  |  |
| ACM 3 | miR-29b-4373288 | Undetermined |  |  |  |
| CTRL 1 | miR-29b-4373288 | Undetermined |  |  |  |
| CTRL 2 | miR-29b-4373288 | Undetermined |  |  |  |
| CTRL 3 | miR-29b-4373288 | Undetermined |  |  |  |
| ACM 1 | miR-29c-4395171 | Undetermined |  |  |  |
| ACM 2 | miR-29c-4395171 | Undetermined |  |  |  |
| ACM 3 | miR-29c-4395171 | Undetermined |  |  |  |
| CTRL 1 | miR-29c-4395171 | Undetermined |  |  |  |
| CTRL 2 | miR-29c-4395171 | Undetermined |  |  |  |
| CTRL 3 | miR-29c-4395171 | Undetermined |  |  |  |
| ACM 1 | miR-301b-4395503 | Undetermined |  |  |  |
| ACM 2 | miR-301b-4395503 | Undetermined |  |  |  |
| ACM 3 | miR-301b-4395503 | Undetermined |  |  |  |
| CTRL 1 | miR-301b-4395503 | Undetermined |  |  |  |
| CTRL 2 | miR-301b-4395503 | Undetermined |  |  |  |
| CTRL 3 | miR-301b-4395503 | Undetermined |  |  |  |
| ACM 1 | miR-302a-4378070 | Undetermined |  |  |  |
| ACM 2 | miR-302a-4378070 | Undetermined |  |  |  |
| ACM 3 | miR-302a-4378070 | Undetermined |  |  |  |
| CTRL 1 | miR-302a-4378070 | Undetermined |  |  |  |
| CTRL 2 | miR-302a-4378070 | Undetermined |  |  |  |
| CTRL 3 | miR-302a-4378070 | Undetermined |  |  |  |
| ACM 1 | miR-302b-4378071 | Undetermined |  |  |  |
| ACM 2 | miR-302b-4378071 | Undetermined |  |  |  |
| ACM 3 | miR-302b-4378071 | Undetermined |  |  |  |
| CTRL 1 | miR-302b-4378071 | Undetermined |  |  |  |
| CTRL 2 | miR-302b-4378071 | Undetermined |  |  |  |
| CTRL 3 | miR-302b-4378071 | Undetermined |  |  |  |
| ACM 1 | miR-302c-4378072 | Undetermined |  |  |  |
| ACM 2 | miR-302c-4378072 | Undetermined |  |  |  |
| ACM 3 | miR-302c-4378072 | Undetermined |  |  |  |
| CTRL 1 | miR-302c-4378072 | Undetermined |  |  |  |
| CTRL 2 | miR-302c-4378072 | Undetermined |  |  |  |
| CTRL 3 | miR-302c-4378072 | Undetermined |  |  |  |
| ACM 1 | miR-31-4395390 | Undetermined |  |  |  |
| ACM 2 | miR-31-4395390 | Undetermined |  |  |  |
| ACM 3 | miR-31-4395390 | Undetermined |  |  |  |
| CTRL 1 | miR-31-4395390 | Undetermined |  |  |  |
| CTRL 2 | miR-31-4395390 | Undetermined |  |  |  |
| CTRL 3 | miR-31-4395390 | Undetermined |  |  |  |
| ACM 1 | miR-323-3p-4395338 | Undetermined |  |  |  |
| ACM 2 | miR-323-3p-4395338 | Undetermined |  |  |  |
| ACM 3 | miR-323-3p-4395338 | Undetermined |  |  |  |
| CTRL 1 | miR-323-3p-4395338 | Undetermined |  |  |  |
| CTRL 2 | miR-323-3p-4395338 | Undetermined |  |  |  |
| CTRL 3 | miR-323-3p-4395338 | Undetermined |  |  |  |
| ACM 1 | miR-32-4395220 | Undetermined |  |  |  |
| ACM 2 | miR-32-4395220 | Undetermined |  |  |  |
| ACM 3 | miR-32-4395220 | Undetermined |  |  |  |
| CTRL 1 | miR-32-4395220 | Undetermined |  |  |  |
| CTRL 2 | miR-32-4395220 | Undetermined |  |  |  |
| CTRL 3 | miR-32-4395220 | Undetermined |  |  |  |
| ACM 1 | miR-325-4373051 | Undetermined |  |  |  |
| ACM 2 | miR-325-4373051 | Undetermined |  |  |  |
| ACM 3 | miR-325-4373051 | Undetermined |  |  |  |
| CTRL 1 | miR-325-4373051 | Undetermined |  |  |  |
| CTRL 2 | miR-325-4373051 | Undetermined |  |  |  |
| CTRL 3 | miR-325-4373051 | Undetermined |  |  |  |
| ACM 1 | miR-326-4373050 | Undetermined |  |  |  |
| ACM 2 | miR-326-4373050 | Undetermined |  |  |  |
| ACM 3 | miR-326-4373050 | Undetermined |  |  |  |
| CTRL 1 | miR-326-4373050 | Undetermined |  |  |  |
| CTRL 2 | miR-326-4373050 | Undetermined |  |  |  |
| CTRL 3 | miR-326-4373050 | Undetermined |  |  |  |
| ACM 1 | miR-329-4373191 | Undetermined |  |  |  |
| ACM 2 | miR-329-4373191 | Undetermined |  |  |  |
| ACM 3 | miR-329-4373191 | Undetermined |  |  |  |
| CTRL 1 | miR-329-4373191 | Undetermined |  |  |  |
| CTRL 2 | miR-329-4373191 | Undetermined |  |  |  |
| CTRL 3 | miR-329-4373191 | Undetermined |  |  |  |
| ACM 1 | miR-330-3p-4373047 | Undetermined |  |  |  |
| ACM 2 | miR-330-3p-4373047 | Undetermined |  |  |  |
| ACM 3 | miR-330-3p-4373047 | Undetermined |  |  |  |
| CTRL 1 | miR-330-3p-4373047 | Undetermined |  |  |  |
| CTRL 2 | miR-330-3p-4373047 | Undetermined |  |  |  |
| CTRL 3 | miR-330-3p-4373047 | Undetermined |  |  |  |
| ACM 1 | miR-330-5p-4395341 | Undetermined |  |  |  |
| ACM 2 | miR-330-5p-4395341 | Undetermined |  |  |  |
| ACM 3 | miR-330-5p-4395341 | Undetermined |  |  |  |
| CTRL 1 | miR-330-5p-4395341 | Undetermined |  |  |  |
| CTRL 2 | miR-330-5p-4395341 | Undetermined |  |  |  |
| CTRL 3 | miR-330-5p-4395341 | Undetermined |  |  |  |
| ACM 1 | miR-331-5p-4395344 | Undetermined |  |  |  |
| ACM 2 | miR-331-5p-4395344 | Undetermined |  |  |  |
| ACM 3 | miR-331-5p-4395344 | Undetermined |  |  |  |
| CTRL 1 | miR-331-5p-4395344 | Undetermined |  |  |  |
| CTRL 2 | miR-331-5p-4395344 | Undetermined |  |  |  |
| CTRL 3 | miR-331-5p-4395344 | Undetermined |  |  |  |
| ACM 1 | miR-337-5p-4395267 | Undetermined |  |  |  |
| ACM 2 | miR-337-5p-4395267 | Undetermined |  |  |  |
| ACM 3 | miR-337-5p-4395267 | Undetermined |  |  |  |
| CTRL 1 | miR-337-5p-4395267 | Undetermined |  |  |  |
| CTRL 2 | miR-337-5p-4395267 | Undetermined |  |  |  |
| CTRL 3 | miR-337-5p-4395267 | Undetermined |  |  |  |
| ACM 1 | miR-338-3p-4395363 | Undetermined |  |  |  |
| ACM 2 | miR-338-3p-4395363 | Undetermined |  |  |  |
| ACM 3 | miR-338-3p-4395363 | Undetermined |  |  |  |
| CTRL 1 | miR-338-3p-4395363 | Undetermined |  |  |  |
| CTRL 2 | miR-338-3p-4395363 | Undetermined |  |  |  |
| CTRL 3 | miR-338-3p-4395363 | Undetermined |  |  |  |
| ACM 1 | miR-339-5p-4395368 | Undetermined |  |  |  |
| ACM 2 | miR-339-5p-4395368 | Undetermined |  |  |  |
| ACM 3 | miR-339-5p-4395368 | Undetermined |  |  |  |
| CTRL 1 | miR-339-5p-4395368 | Undetermined |  |  |  |
| CTRL 2 | miR-339-5p-4395368 | Undetermined |  |  |  |
| CTRL 3 | miR-339-5p-4395368 | Undetermined |  |  |  |
| ACM 1 | miR-33b-4395196 | Undetermined |  |  |  |
| ACM 2 | miR-33b-4395196 | Undetermined |  |  |  |
| ACM 3 | miR-33b-4395196 | Undetermined |  |  |  |
| CTRL 1 | miR-33b-4395196 | Undetermined |  |  |  |
| CTRL 2 | miR-33b-4395196 | Undetermined |  |  |  |
| CTRL 3 | miR-33b-4395196 | Undetermined |  |  |  |
| ACM 1 | miR-342-5p-4395258 | Undetermined |  |  |  |
| ACM 2 | miR-342-5p-4395258 | Undetermined |  |  |  |
| ACM 3 | miR-342-5p-4395258 | Undetermined |  |  |  |
| CTRL 1 | miR-342-5p-4395258 | Undetermined |  |  |  |
| CTRL 2 | miR-342-5p-4395258 | Undetermined |  |  |  |
| CTRL 3 | miR-342-5p-4395258 | Undetermined |  |  |  |
| ACM 1 | miR-346-4373038 | Undetermined |  |  |  |
| ACM 2 | miR-346-4373038 | Undetermined |  |  |  |
| ACM 3 | miR-346-4373038 | Undetermined |  |  |  |
| CTRL 1 | miR-346-4373038 | Undetermined |  |  |  |
| CTRL 2 | miR-346-4373038 | Undetermined |  |  |  |
| CTRL 3 | miR-346-4373038 | Undetermined |  |  |  |
| ACM 1 | miR-34c-5p-4373036 | Undetermined |  |  |  |
| ACM 2 | miR-34c-5p-4373036 | Undetermined |  |  |  |
| ACM 3 | miR-34c-5p-4373036 | Undetermined |  |  |  |
| CTRL 1 | miR-34c-5p-4373036 | Undetermined |  |  |  |
| CTRL 2 | miR-34c-5p-4373036 | Undetermined |  |  |  |
| CTRL 3 | miR-34c-5p-4373036 | Undetermined |  |  |  |
| ACM 1 | miR-361-5p-4373035 | Undetermined |  |  |  |
| ACM 2 | miR-361-5p-4373035 | Undetermined |  |  |  |
| ACM 3 | miR-361-5p-4373035 | Undetermined |  |  |  |
| CTRL 1 | miR-361-5p-4373035 | Undetermined |  |  |  |
| CTRL 2 | miR-361-5p-4373035 | Undetermined |  |  |  |
| CTRL 3 | miR-361-5p-4373035 | Undetermined |  |  |  |
| ACM 1 | miR-362-3p-4395228 | Undetermined |  |  |  |
| ACM 2 | miR-362-3p-4395228 | Undetermined |  |  |  |
| ACM 3 | miR-362-3p-4395228 | Undetermined |  |  |  |
| CTRL 1 | miR-362-3p-4395228 | Undetermined |  |  |  |
| CTRL 2 | miR-362-3p-4395228 | Undetermined |  |  |  |
| CTRL 3 | miR-362-3p-4395228 | Undetermined |  |  |  |
| ACM 1 | miR-362-5p-4378092 | Undetermined |  |  |  |
| ACM 2 | miR-362-5p-4378092 | Undetermined |  |  |  |
| ACM 3 | miR-362-5p-4378092 | Undetermined |  |  |  |
| CTRL 1 | miR-362-5p-4378092 | Undetermined |  |  |  |
| CTRL 2 | miR-362-5p-4378092 | Undetermined |  |  |  |
| CTRL 3 | miR-362-5p-4378092 | Undetermined |  |  |  |
| ACM 1 | miR-367-4373034 | Undetermined |  |  |  |
| ACM 2 | miR-367-4373034 | Undetermined |  |  |  |
| ACM 3 | miR-367-4373034 | Undetermined |  |  |  |
| CTRL 1 | miR-367-4373034 | Undetermined |  |  |  |
| CTRL 2 | miR-367-4373034 | Undetermined |  |  |  |
| CTRL 3 | miR-367-4373034 | Undetermined |  |  |  |
| ACM 1 | miR-369-3p-4373032 | Undetermined |  |  |  |
| ACM 2 | miR-369-3p-4373032 | Undetermined |  |  |  |
| ACM 3 | miR-369-3p-4373032 | Undetermined |  |  |  |
| CTRL 1 | miR-369-3p-4373032 | Undetermined |  |  |  |
| CTRL 2 | miR-369-3p-4373032 | Undetermined |  |  |  |
| CTRL 3 | miR-369-3p-4373032 | Undetermined |  |  |  |
| ACM 1 | miR-369-5p-4373195 | Undetermined |  |  |  |
| ACM 2 | miR-369-5p-4373195 | Undetermined |  |  |  |
| ACM 3 | miR-369-5p-4373195 | Undetermined |  |  |  |
| CTRL 1 | miR-369-5p-4373195 | Undetermined |  |  |  |
| CTRL 2 | miR-369-5p-4373195 | Undetermined |  |  |  |
| CTRL 3 | miR-369-5p-4373195 | Undetermined |  |  |  |
| ACM 1 | miR-371-3p-4395235 | Undetermined |  |  |  |
| ACM 2 | miR-371-3p-4395235 | Undetermined |  |  |  |
| ACM 3 | miR-371-3p-4395235 | Undetermined |  |  |  |
| CTRL 1 | miR-371-3p-4395235 | Undetermined |  |  |  |
| CTRL 2 | miR-371-3p-4395235 | Undetermined |  |  |  |
| CTRL 3 | miR-371-3p-4395235 | Undetermined |  |  |  |
| ACM 1 | miR-372-4373029 | Undetermined |  |  |  |
| ACM 2 | miR-372-4373029 | Undetermined |  |  |  |
| ACM 3 | miR-372-4373029 | Undetermined |  |  |  |
| CTRL 1 | miR-372-4373029 | Undetermined |  |  |  |
| CTRL 2 | miR-372-4373029 | Undetermined |  |  |  |
| CTRL 3 | miR-372-4373029 | Undetermined |  |  |  |
| ACM 1 | miR-373-4378073 | Undetermined |  |  |  |
| ACM 2 | miR-373-4378073 | Undetermined |  |  |  |
| ACM 3 | miR-373-4378073 | Undetermined |  |  |  |
| CTRL 1 | miR-373-4378073 | Undetermined |  |  |  |
| CTRL 2 | miR-373-4378073 | Undetermined |  |  |  |
| CTRL 3 | miR-373-4378073 | Undetermined |  |  |  |
| ACM 1 | miR-376b-4373196 | Undetermined |  |  |  |
| ACM 2 | miR-376b-4373196 | Undetermined |  |  |  |
| ACM 3 | miR-376b-4373196 | Undetermined |  |  |  |
| CTRL 1 | miR-376b-4373196 | Undetermined |  |  |  |
| CTRL 2 | miR-376b-4373196 | Undetermined |  |  |  |
| CTRL 3 | miR-376b-4373196 | Undetermined |  |  |  |
| ACM 1 | miR-376c-4395233 | Undetermined |  |  |  |
| ACM 2 | miR-376c-4395233 | Undetermined |  |  |  |
| ACM 3 | miR-376c-4395233 | Undetermined |  |  |  |
| CTRL 1 | miR-376c-4395233 | Undetermined |  |  |  |
| CTRL 2 | miR-376c-4395233 | Undetermined |  |  |  |
| CTRL 3 | miR-376c-4395233 | Undetermined |  |  |  |
| ACM 1 | miR-377-4373025 | Undetermined |  |  |  |
| ACM 2 | miR-377-4373025 | Undetermined |  |  |  |
| ACM 3 | miR-377-4373025 | Undetermined |  |  |  |
| CTRL 1 | miR-377-4373025 | Undetermined |  |  |  |
| CTRL 2 | miR-377-4373025 | Undetermined |  |  |  |
| CTRL 3 | miR-377-4373025 | Undetermined |  |  |  |
| ACM 1 | miR-379-4373349 | Undetermined |  |  |  |
| ACM 2 | miR-379-4373349 | Undetermined |  |  |  |
| ACM 3 | miR-379-4373349 | Undetermined |  |  |  |
| CTRL 1 | miR-379-4373349 | Undetermined |  |  |  |
| CTRL 2 | miR-379-4373349 | Undetermined |  |  |  |
| CTRL 3 | miR-379-4373349 | Undetermined |  |  |  |
| ACM 1 | miR-380-4373022 | Undetermined |  |  |  |
| ACM 2 | miR-380-4373022 | Undetermined |  |  |  |
| ACM 3 | miR-380-4373022 | Undetermined |  |  |  |
| CTRL 1 | miR-380-4373022 | Undetermined |  |  |  |
| CTRL 2 | miR-380-4373022 | Undetermined |  |  |  |
| CTRL 3 | miR-380-4373022 | Undetermined |  |  |  |
| ACM 1 | miR-381-4373020 | Undetermined |  |  |  |
| ACM 2 | miR-381-4373020 | Undetermined |  |  |  |
| ACM 3 | miR-381-4373020 | Undetermined |  |  |  |
| CTRL 1 | miR-381-4373020 | Undetermined |  |  |  |
| CTRL 2 | miR-381-4373020 | Undetermined |  |  |  |
| CTRL 3 | miR-381-4373020 | Undetermined |  |  |  |
| ACM 1 | miR-382-4373019 | Undetermined |  |  |  |
| ACM 2 | miR-382-4373019 | Undetermined |  |  |  |
| ACM 3 | miR-382-4373019 | Undetermined |  |  |  |
| CTRL 1 | miR-382-4373019 | Undetermined |  |  |  |
| CTRL 2 | miR-382-4373019 | Undetermined |  |  |  |
| CTRL 3 | miR-382-4373019 | Undetermined |  |  |  |
| ACM 1 | miR-383-4373018 | Undetermined |  |  |  |
| ACM 2 | miR-383-4373018 | Undetermined |  |  |  |
| ACM 3 | miR-383-4373018 | Undetermined |  |  |  |
| CTRL 1 | miR-383-4373018 | Undetermined |  |  |  |
| CTRL 2 | miR-383-4373018 | Undetermined |  |  |  |
| CTRL 3 | miR-383-4373018 | Undetermined |  |  |  |
| ACM 1 | miR-384-4373017 | Undetermined |  |  |  |
| ACM 2 | miR-384-4373017 | Undetermined |  |  |  |
| ACM 3 | miR-384-4373017 | Undetermined |  |  |  |
| CTRL 1 | miR-384-4373017 | Undetermined |  |  |  |
| CTRL 2 | miR-384-4373017 | Undetermined |  |  |  |
| CTRL 3 | miR-384-4373017 | Undetermined |  |  |  |
| ACM 1 | miR-409-5p-4395442 | Undetermined |  |  |  |
| ACM 2 | miR-409-5p-4395442 | Undetermined |  |  |  |
| ACM 3 | miR-409-5p-4395442 | Undetermined |  |  |  |
| CTRL 1 | miR-409-5p-4395442 | Undetermined |  |  |  |
| CTRL 2 | miR-409-5p-4395442 | Undetermined |  |  |  |
| CTRL 3 | miR-409-5p-4395442 | Undetermined |  |  |  |
| ACM 1 | miR-410-4378093 | Undetermined |  |  |  |
| ACM 2 | miR-410-4378093 | Undetermined |  |  |  |
| ACM 3 | miR-410-4378093 | Undetermined |  |  |  |
| CTRL 1 | miR-410-4378093 | Undetermined |  |  |  |
| CTRL 2 | miR-410-4378093 | Undetermined |  |  |  |
| CTRL 3 | miR-410-4378093 | Undetermined |  |  |  |
| ACM 1 | miR-411-4381013 | Undetermined |  |  |  |
| ACM 2 | miR-411-4381013 | Undetermined |  |  |  |
| ACM 3 | miR-411-4381013 | Undetermined |  |  |  |
| CTRL 1 | miR-411-4381013 | Undetermined |  |  |  |
| CTRL 2 | miR-411-4381013 | Undetermined |  |  |  |
| CTRL 3 | miR-411-4381013 | Undetermined |  |  |  |
| ACM 1 | miR-412-4373199 | Undetermined |  |  |  |
| ACM 2 | miR-412-4373199 | Undetermined |  |  |  |
| ACM 3 | miR-412-4373199 | Undetermined |  |  |  |
| CTRL 1 | miR-412-4373199 | Undetermined |  |  |  |
| CTRL 2 | miR-412-4373199 | Undetermined |  |  |  |
| CTRL 3 | miR-412-4373199 | Undetermined |  |  |  |
| ACM 1 | miR-422a-4395408 | Undetermined |  |  |  |
| ACM 2 | miR-422a-4395408 | Undetermined |  |  |  |
| ACM 3 | miR-422a-4395408 | Undetermined |  |  |  |
| CTRL 1 | miR-422a-4395408 | Undetermined |  |  |  |
| CTRL 2 | miR-422a-4395408 | Undetermined |  |  |  |
| CTRL 3 | miR-422a-4395408 | Undetermined |  |  |  |
| ACM 1 | miR-423-5p-4395451 | Undetermined |  |  |  |
| ACM 2 | miR-423-5p-4395451 | Undetermined |  |  |  |
| ACM 3 | miR-423-5p-4395451 | Undetermined |  |  |  |
| CTRL 1 | miR-423-5p-4395451 | Undetermined |  |  |  |
| CTRL 2 | miR-423-5p-4395451 | Undetermined |  |  |  |
| CTRL 3 | miR-423-5p-4395451 | Undetermined |  |  |  |
| ACM 1 | miR-424-4373201 | Undetermined |  |  |  |
| ACM 2 | miR-424-4373201 | Undetermined |  |  |  |
| ACM 3 | miR-424-4373201 | Undetermined |  |  |  |
| CTRL 1 | miR-424-4373201 | Undetermined |  |  |  |
| CTRL 2 | miR-424-4373201 | Undetermined |  |  |  |
| CTRL 3 | miR-424-4373201 | Undetermined |  |  |  |
| ACM 1 | miR-425-4380926 | Undetermined |  |  |  |
| ACM 2 | miR-425-4380926 | Undetermined |  |  |  |
| ACM 3 | miR-425-4380926 | Undetermined |  |  |  |
| CTRL 1 | miR-425-4380926 | Undetermined |  |  |  |
| CTRL 2 | miR-425-4380926 | Undetermined |  |  |  |
| CTRL 3 | miR-425-4380926 | Undetermined |  |  |  |
| ACM 1 | miR-429-4373203 | Undetermined |  |  |  |
| ACM 2 | miR-429-4373203 | Undetermined |  |  |  |
| ACM 3 | miR-429-4373203 | Undetermined |  |  |  |
| CTRL 1 | miR-429-4373203 | Undetermined |  |  |  |
| CTRL 2 | miR-429-4373203 | Undetermined |  |  |  |
| CTRL 3 | miR-429-4373203 | Undetermined |  |  |  |
| ACM 1 | miR-431-4395173 | Undetermined |  |  |  |
| ACM 2 | miR-431-4395173 | Undetermined |  |  |  |
| ACM 3 | miR-431-4395173 | Undetermined |  |  |  |
| CTRL 1 | miR-431-4395173 | Undetermined |  |  |  |
| CTRL 2 | miR-431-4395173 | Undetermined |  |  |  |
| CTRL 3 | miR-431-4395173 | Undetermined |  |  |  |
| ACM 1 | miR-433-4373205 | Undetermined |  |  |  |
| ACM 2 | miR-433-4373205 | Undetermined |  |  |  |
| ACM 3 | miR-433-4373205 | Undetermined |  |  |  |
| CTRL 1 | miR-433-4373205 | Undetermined |  |  |  |
| CTRL 2 | miR-433-4373205 | Undetermined |  |  |  |
| CTRL 3 | miR-433-4373205 | Undetermined |  |  |  |
| ACM 1 | miR-448-4373206 | Undetermined |  |  |  |
| ACM 2 | miR-448-4373206 | Undetermined |  |  |  |
| ACM 3 | miR-448-4373206 | Undetermined |  |  |  |
| CTRL 1 | miR-448-4373206 | Undetermined |  |  |  |
| CTRL 2 | miR-448-4373206 | Undetermined |  |  |  |
| CTRL 3 | miR-448-4373206 | Undetermined |  |  |  |
| ACM 1 | miR-449a-4373207 | Undetermined |  |  |  |
| ACM 2 | miR-449a-4373207 | Undetermined |  |  |  |
| ACM 3 | miR-449a-4373207 | Undetermined |  |  |  |
| CTRL 1 | miR-449a-4373207 | Undetermined |  |  |  |
| CTRL 2 | miR-449a-4373207 | Undetermined |  |  |  |
| CTRL 3 | miR-449a-4373207 | Undetermined |  |  |  |
| ACM 1 | miR-449b-4381011 | Undetermined |  |  |  |
| ACM 2 | miR-449b-4381011 | Undetermined |  |  |  |
| ACM 3 | miR-449b-4381011 | Undetermined |  |  |  |
| CTRL 1 | miR-449b-4381011 | Undetermined |  |  |  |
| CTRL 2 | miR-449b-4381011 | Undetermined |  |  |  |
| CTRL 3 | miR-449b-4381011 | Undetermined |  |  |  |
| ACM 1 | miR-450a-4395414 | Undetermined |  |  |  |
| ACM 2 | miR-450a-4395414 | Undetermined |  |  |  |
| ACM 3 | miR-450a-4395414 | Undetermined |  |  |  |
| CTRL 1 | miR-450a-4395414 | Undetermined |  |  |  |
| CTRL 2 | miR-450a-4395414 | Undetermined |  |  |  |
| CTRL 3 | miR-450a-4395414 | Undetermined |  |  |  |
| ACM 1 | miR-450b-3p-4395319 | Undetermined |  |  |  |
| ACM 2 | miR-450b-3p-4395319 | Undetermined |  |  |  |
| ACM 3 | miR-450b-3p-4395319 | Undetermined |  |  |  |
| CTRL 1 | miR-450b-3p-4395319 | Undetermined |  |  |  |
| CTRL 2 | miR-450b-3p-4395319 | Undetermined |  |  |  |
| CTRL 3 | miR-450b-3p-4395319 | Undetermined |  |  |  |
| ACM 1 | miR-450b-5p-4395318 | Undetermined |  |  |  |
| ACM 2 | miR-450b-5p-4395318 | Undetermined |  |  |  |
| ACM 3 | miR-450b-5p-4395318 | Undetermined |  |  |  |
| CTRL 1 | miR-450b-5p-4395318 | Undetermined |  |  |  |
| CTRL 2 | miR-450b-5p-4395318 | Undetermined |  |  |  |
| CTRL 3 | miR-450b-5p-4395318 | Undetermined |  |  |  |
| ACM 1 | miR-452-4395440 | Undetermined |  |  |  |
| ACM 2 | miR-452-4395440 | Undetermined |  |  |  |
| ACM 3 | miR-452-4395440 | Undetermined |  |  |  |
| CTRL 1 | miR-452-4395440 | Undetermined |  |  |  |
| CTRL 2 | miR-452-4395440 | Undetermined |  |  |  |
| CTRL 3 | miR-452-4395440 | Undetermined |  |  |  |
| ACM 1 | miR-453-4395429 | Undetermined |  |  |  |
| ACM 2 | miR-453-4395429 | Undetermined |  |  |  |
| ACM 3 | miR-453-4395429 | Undetermined |  |  |  |
| CTRL 1 | miR-453-4395429 | Undetermined |  |  |  |
| CTRL 2 | miR-453-4395429 | Undetermined |  |  |  |
| CTRL 3 | miR-453-4395429 | Undetermined |  |  |  |
| ACM 1 | miR-454-4395434 | Undetermined |  |  |  |
| ACM 2 | miR-454-4395434 | Undetermined |  |  |  |
| ACM 3 | miR-454-4395434 | Undetermined |  |  |  |
| CTRL 1 | miR-454-4395434 | Undetermined |  |  |  |
| CTRL 2 | miR-454-4395434 | Undetermined |  |  |  |
| CTRL 3 | miR-454-4395434 | Undetermined |  |  |  |
| ACM 1 | miR-455-3p-4395355 | Undetermined |  |  |  |
| ACM 2 | miR-455-3p-4395355 | Undetermined |  |  |  |
| ACM 3 | miR-455-3p-4395355 | Undetermined |  |  |  |
| CTRL 1 | miR-455-3p-4395355 | Undetermined |  |  |  |
| CTRL 2 | miR-455-3p-4395355 | Undetermined |  |  |  |
| CTRL 3 | miR-455-3p-4395355 | Undetermined |  |  |  |
| ACM 1 | miR-455-5p-4378098 | Undetermined |  |  |  |
| ACM 2 | miR-455-5p-4378098 | Undetermined |  |  |  |
| ACM 3 | miR-455-5p-4378098 | Undetermined |  |  |  |
| CTRL 1 | miR-455-5p-4378098 | Undetermined |  |  |  |
| CTRL 2 | miR-455-5p-4378098 | Undetermined |  |  |  |
| CTRL 3 | miR-455-5p-4378098 | Undetermined |  |  |  |
| ACM 1 | miR-484-4381032 | Undetermined |  |  |  |
| ACM 2 | miR-484-4381032 | Undetermined |  |  |  |
| ACM 3 | miR-484-4381032 | Undetermined |  |  |  |
| CTRL 1 | miR-484-4381032 | Undetermined |  |  |  |
| CTRL 2 | miR-484-4381032 | Undetermined |  |  |  |
| CTRL 3 | miR-484-4381032 | Undetermined |  |  |  |
| ACM 1 | miR-485-3p-4378095 | Undetermined |  |  |  |
| ACM 2 | miR-485-3p-4378095 | Undetermined |  |  |  |
| ACM 3 | miR-485-3p-4378095 | Undetermined |  |  |  |
| CTRL 1 | miR-485-3p-4378095 | Undetermined |  |  |  |
| CTRL 2 | miR-485-3p-4378095 | Undetermined |  |  |  |
| CTRL 3 | miR-485-3p-4378095 | Undetermined |  |  |  |
| ACM 1 | miR-485-5p-4373212 | Undetermined |  |  |  |
| ACM 2 | miR-485-5p-4373212 | Undetermined |  |  |  |
| ACM 3 | miR-485-5p-4373212 | Undetermined |  |  |  |
| CTRL 1 | miR-485-5p-4373212 | Undetermined |  |  |  |
| CTRL 2 | miR-485-5p-4373212 | Undetermined |  |  |  |
| CTRL 3 | miR-485-5p-4373212 | Undetermined |  |  |  |
| ACM 1 | miR-487a-4378097 | Undetermined |  |  |  |
| ACM 2 | miR-487a-4378097 | Undetermined |  |  |  |
| ACM 3 | miR-487a-4378097 | Undetermined |  |  |  |
| CTRL 1 | miR-487a-4378097 | Undetermined |  |  |  |
| CTRL 2 | miR-487a-4378097 | Undetermined |  |  |  |
| CTRL 3 | miR-487a-4378097 | Undetermined |  |  |  |
| ACM 1 | miR-487b-4378102 | Undetermined |  |  |  |
| ACM 2 | miR-487b-4378102 | Undetermined |  |  |  |
| ACM 3 | miR-487b-4378102 | Undetermined |  |  |  |
| CTRL 1 | miR-487b-4378102 | Undetermined |  |  |  |
| CTRL 2 | miR-487b-4378102 | Undetermined |  |  |  |
| CTRL 3 | miR-487b-4378102 | Undetermined |  |  |  |
| ACM 1 | miR-488-4395468 | Undetermined |  |  |  |
| ACM 2 | miR-488-4395468 | Undetermined |  |  |  |
| ACM 3 | miR-488-4395468 | Undetermined |  |  |  |
| CTRL 1 | miR-488-4395468 | Undetermined |  |  |  |
| CTRL 2 | miR-488-4395468 | Undetermined |  |  |  |
| CTRL 3 | miR-488-4395468 | Undetermined |  |  |  |
| ACM 1 | miR-489-4395469 | Undetermined |  |  |  |
| ACM 2 | miR-489-4395469 | Undetermined |  |  |  |
| ACM 3 | miR-489-4395469 | Undetermined |  |  |  |
| CTRL 1 | miR-489-4395469 | Undetermined |  |  |  |
| CTRL 2 | miR-489-4395469 | Undetermined |  |  |  |
| CTRL 3 | miR-489-4395469 | Undetermined |  |  |  |
| ACM 1 | miR-490-3p-4373215 | Undetermined |  |  |  |
| ACM 2 | miR-490-3p-4373215 | Undetermined |  |  |  |
| ACM 3 | miR-490-3p-4373215 | Undetermined |  |  |  |
| CTRL 1 | miR-490-3p-4373215 | Undetermined |  |  |  |
| CTRL 2 | miR-490-3p-4373215 | Undetermined |  |  |  |
| CTRL 3 | miR-490-3p-4373215 | Undetermined |  |  |  |
| ACM 1 | miR-491-3p-4395471 | Undetermined |  |  |  |
| ACM 2 | miR-491-3p-4395471 | Undetermined |  |  |  |
| ACM 3 | miR-491-3p-4395471 | Undetermined |  |  |  |
| CTRL 1 | miR-491-3p-4395471 | Undetermined |  |  |  |
| CTRL 2 | miR-491-3p-4395471 | Undetermined |  |  |  |
| CTRL 3 | miR-491-3p-4395471 | Undetermined |  |  |  |
| ACM 1 | miR-491-5p-4381053 | Undetermined |  |  |  |
| ACM 2 | miR-491-5p-4381053 | Undetermined |  |  |  |
| ACM 3 | miR-491-5p-4381053 | Undetermined |  |  |  |
| CTRL 1 | miR-491-5p-4381053 | Undetermined |  |  |  |
| CTRL 2 | miR-491-5p-4381053 | Undetermined |  |  |  |
| CTRL 3 | miR-491-5p-4381053 | Undetermined |  |  |  |
| ACM 1 | miR-492-4373217 | Undetermined |  |  |  |
| ACM 2 | miR-492-4373217 | Undetermined |  |  |  |
| ACM 3 | miR-492-4373217 | Undetermined |  |  |  |
| CTRL 1 | miR-492-4373217 | Undetermined |  |  |  |
| CTRL 2 | miR-492-4373217 | Undetermined |  |  |  |
| CTRL 3 | miR-492-4373217 | Undetermined |  |  |  |
| ACM 1 | miR-493-4395475 | Undetermined |  |  |  |
| ACM 2 | miR-493-4395475 | Undetermined |  |  |  |
| ACM 3 | miR-493-4395475 | Undetermined |  |  |  |
| CTRL 1 | miR-493-4395475 | Undetermined |  |  |  |
| CTRL 2 | miR-493-4395475 | Undetermined |  |  |  |
| CTRL 3 | miR-493-4395475 | Undetermined |  |  |  |
| ACM 1 | miR-494-4395476 | Undetermined |  |  |  |
| ACM 2 | miR-494-4395476 | Undetermined |  |  |  |
| ACM 3 | miR-494-4395476 | Undetermined |  |  |  |
| CTRL 1 | miR-494-4395476 | Undetermined |  |  |  |
| CTRL 2 | miR-494-4395476 | Undetermined |  |  |  |
| CTRL 3 | miR-494-4395476 | Undetermined |  |  |  |
| ACM 1 | miR-495-4381078 | Undetermined |  |  |  |
| ACM 2 | miR-495-4381078 | Undetermined |  |  |  |
| ACM 3 | miR-495-4381078 | Undetermined |  |  |  |
| CTRL 1 | miR-495-4381078 | Undetermined |  |  |  |
| CTRL 2 | miR-495-4381078 | Undetermined |  |  |  |
| CTRL 3 | miR-495-4381078 | Undetermined |  |  |  |
| ACM 1 | miR-496-4386771 | Undetermined |  |  |  |
| ACM 2 | miR-496-4386771 | Undetermined |  |  |  |
| ACM 3 | miR-496-4386771 | Undetermined |  |  |  |
| CTRL 1 | miR-496-4386771 | Undetermined |  |  |  |
| CTRL 2 | miR-496-4386771 | Undetermined |  |  |  |
| CTRL 3 | miR-496-4386771 | Undetermined |  |  |  |
| ACM 1 | miR-499-3p-4395538 | Undetermined |  |  |  |
| ACM 2 | miR-499-3p-4395538 | Undetermined |  |  |  |
| ACM 3 | miR-499-3p-4395538 | Undetermined |  |  |  |
| CTRL 1 | miR-499-3p-4395538 | Undetermined |  |  |  |
| CTRL 2 | miR-499-3p-4395538 | Undetermined |  |  |  |
| CTRL 3 | miR-499-3p-4395538 | Undetermined |  |  |  |
| ACM 1 | miR-499-5p-4381047 | Undetermined |  |  |  |
| ACM 2 | miR-499-5p-4381047 | Undetermined |  |  |  |
| ACM 3 | miR-499-5p-4381047 | Undetermined |  |  |  |
| CTRL 1 | miR-499-5p-4381047 | Undetermined |  |  |  |
| CTRL 2 | miR-499-5p-4381047 | Undetermined |  |  |  |
| CTRL 3 | miR-499-5p-4381047 | Undetermined |  |  |  |
| ACM 1 | miR-501-3p-4395546 | Undetermined |  |  |  |
| ACM 2 | miR-501-3p-4395546 | Undetermined |  |  |  |
| ACM 3 | miR-501-3p-4395546 | Undetermined |  |  |  |
| CTRL 1 | miR-501-3p-4395546 | Undetermined |  |  |  |
| CTRL 2 | miR-501-3p-4395546 | Undetermined |  |  |  |
| CTRL 3 | miR-501-3p-4395546 | Undetermined |  |  |  |
| ACM 1 | miR-503-4373228 | Undetermined |  |  |  |
| ACM 2 | miR-503-4373228 | Undetermined |  |  |  |
| ACM 3 | miR-503-4373228 | Undetermined |  |  |  |
| CTRL 1 | miR-503-4373228 | Undetermined |  |  |  |
| CTRL 2 | miR-503-4373228 | Undetermined |  |  |  |
| CTRL 3 | miR-503-4373228 | Undetermined |  |  |  |
| ACM 1 | miR-504-4395195 | Undetermined |  |  |  |
| ACM 2 | miR-504-4395195 | Undetermined |  |  |  |
| ACM 3 | miR-504-4395195 | Undetermined |  |  |  |
| CTRL 1 | miR-504-4395195 | Undetermined |  |  |  |
| CTRL 2 | miR-504-4395195 | Undetermined |  |  |  |
| CTRL 3 | miR-504-4395195 | Undetermined |  |  |  |
| ACM 1 | miR-505-4395200 | Undetermined |  |  |  |
| ACM 2 | miR-505-4395200 | Undetermined |  |  |  |
| ACM 3 | miR-505-4395200 | Undetermined |  |  |  |
| CTRL 1 | miR-505-4395200 | Undetermined |  |  |  |
| CTRL 2 | miR-505-4395200 | Undetermined |  |  |  |
| CTRL 3 | miR-505-4395200 | Undetermined |  |  |  |
| ACM 1 | miR-506-4373231 | Undetermined |  |  |  |
| ACM 2 | miR-506-4373231 | Undetermined |  |  |  |
| ACM 3 | miR-506-4373231 | Undetermined |  |  |  |
| CTRL 1 | miR-506-4373231 | Undetermined |  |  |  |
| CTRL 2 | miR-506-4373231 | Undetermined |  |  |  |
| CTRL 3 | miR-506-4373231 | Undetermined |  |  |  |
| ACM 1 | miR-507-4373232 | Undetermined |  |  |  |
| ACM 2 | miR-507-4373232 | Undetermined |  |  |  |
| ACM 3 | miR-507-4373232 | Undetermined |  |  |  |
| CTRL 1 | miR-507-4373232 | Undetermined |  |  |  |
| CTRL 2 | miR-507-4373232 | Undetermined |  |  |  |
| CTRL 3 | miR-507-4373232 | Undetermined |  |  |  |
| ACM 1 | miR-508-3p-4373233 | Undetermined |  |  |  |
| ACM 2 | miR-508-3p-4373233 | Undetermined |  |  |  |
| ACM 3 | miR-508-3p-4373233 | Undetermined |  |  |  |
| CTRL 1 | miR-508-3p-4373233 | Undetermined |  |  |  |
| CTRL 2 | miR-508-3p-4373233 | Undetermined |  |  |  |
| CTRL 3 | miR-508-3p-4373233 | Undetermined |  |  |  |
| ACM 1 | miR-508-5p-4395203 | Undetermined |  |  |  |
| ACM 2 | miR-508-5p-4395203 | Undetermined |  |  |  |
| ACM 3 | miR-508-5p-4395203 | Undetermined |  |  |  |
| CTRL 1 | miR-508-5p-4395203 | Undetermined |  |  |  |
| CTRL 2 | miR-508-5p-4395203 | Undetermined |  |  |  |
| CTRL 3 | miR-508-5p-4395203 | Undetermined |  |  |  |
| ACM 1 | miR-509-3-5p-4395266 | Undetermined |  |  |  |
| ACM 2 | miR-509-3-5p-4395266 | Undetermined |  |  |  |
| ACM 3 | miR-509-3-5p-4395266 | Undetermined |  |  |  |
| CTRL 1 | miR-509-3-5p-4395266 | Undetermined |  |  |  |
| CTRL 2 | miR-509-3-5p-4395266 | Undetermined |  |  |  |
| CTRL 3 | miR-509-3-5p-4395266 | Undetermined |  |  |  |
| ACM 1 | miR-509-5p-4395346 | Undetermined |  |  |  |
| ACM 2 | miR-509-5p-4395346 | Undetermined |  |  |  |
| ACM 3 | miR-509-5p-4395346 | Undetermined |  |  |  |
| CTRL 1 | miR-509-5p-4395346 | Undetermined |  |  |  |
| CTRL 2 | miR-509-5p-4395346 | Undetermined |  |  |  |
| CTRL 3 | miR-509-5p-4395346 | Undetermined |  |  |  |
| ACM 1 | miR-510-4395352 | Undetermined |  |  |  |
| ACM 2 | miR-510-4395352 | Undetermined |  |  |  |
| ACM 3 | miR-510-4395352 | Undetermined |  |  |  |
| CTRL 1 | miR-510-4395352 | Undetermined |  |  |  |
| CTRL 2 | miR-510-4395352 | Undetermined |  |  |  |
| CTRL 3 | miR-510-4395352 | Undetermined |  |  |  |
| ACM 1 | miR-511-4373236 | Undetermined |  |  |  |
| ACM 2 | miR-511-4373236 | Undetermined |  |  |  |
| ACM 3 | miR-511-4373236 | Undetermined |  |  |  |
| CTRL 1 | miR-511-4373236 | Undetermined |  |  |  |
| CTRL 2 | miR-511-4373236 | Undetermined |  |  |  |
| CTRL 3 | miR-511-4373236 | Undetermined |  |  |  |
| ACM 1 | miR-512-3p-4381034 | Undetermined |  |  |  |
| ACM 2 | miR-512-3p-4381034 | Undetermined |  |  |  |
| ACM 3 | miR-512-3p-4381034 | Undetermined |  |  |  |
| CTRL 1 | miR-512-3p-4381034 | Undetermined |  |  |  |
| CTRL 2 | miR-512-3p-4381034 | Undetermined |  |  |  |
| CTRL 3 | miR-512-3p-4381034 | Undetermined |  |  |  |
| ACM 1 | miR-512-5p-4373238 | Undetermined |  |  |  |
| ACM 2 | miR-512-5p-4373238 | Undetermined |  |  |  |
| ACM 3 | miR-512-5p-4373238 | Undetermined |  |  |  |
| CTRL 1 | miR-512-5p-4373238 | Undetermined |  |  |  |
| CTRL 2 | miR-512-5p-4373238 | Undetermined |  |  |  |
| CTRL 3 | miR-512-5p-4373238 | Undetermined |  |  |  |
| ACM 1 | miR-513-5p-4395201 | Undetermined |  |  |  |
| ACM 2 | miR-513-5p-4395201 | Undetermined |  |  |  |
| ACM 3 | miR-513-5p-4395201 | Undetermined |  |  |  |
| CTRL 1 | miR-513-5p-4395201 | Undetermined |  |  |  |
| CTRL 2 | miR-513-5p-4395201 | Undetermined |  |  |  |
| CTRL 3 | miR-513-5p-4395201 | Undetermined |  |  |  |
| ACM 1 | miR-515-3p-4395480 | Undetermined |  |  |  |
| ACM 2 | miR-515-3p-4395480 | Undetermined |  |  |  |
| ACM 3 | miR-515-3p-4395480 | Undetermined |  |  |  |
| CTRL 1 | miR-515-3p-4395480 | Undetermined |  |  |  |
| CTRL 2 | miR-515-3p-4395480 | Undetermined |  |  |  |
| CTRL 3 | miR-515-3p-4395480 | Undetermined |  |  |  |
| ACM 1 | miR-515-5p-4373242 | Undetermined |  |  |  |
| ACM 2 | miR-515-5p-4373242 | Undetermined |  |  |  |
| ACM 3 | miR-515-5p-4373242 | Undetermined |  |  |  |
| CTRL 1 | miR-515-5p-4373242 | Undetermined |  |  |  |
| CTRL 2 | miR-515-5p-4373242 | Undetermined |  |  |  |
| CTRL 3 | miR-515-5p-4373242 | Undetermined |  |  |  |
| ACM 1 | miR-516a-5p-4395527 | Undetermined |  |  |  |
| ACM 2 | miR-516a-5p-4395527 | Undetermined |  |  |  |
| ACM 3 | miR-516a-5p-4395527 | Undetermined |  |  |  |
| CTRL 1 | miR-516a-5p-4395527 | Undetermined |  |  |  |
| CTRL 2 | miR-516a-5p-4395527 | Undetermined |  |  |  |
| CTRL 3 | miR-516a-5p-4395527 | Undetermined |  |  |  |
| ACM 1 | miR-516b-4395172 | Undetermined |  |  |  |
| ACM 2 | miR-516b-4395172 | Undetermined |  |  |  |
| ACM 3 | miR-516b-4395172 | Undetermined |  |  |  |
| CTRL 1 | miR-516b-4395172 | Undetermined |  |  |  |
| CTRL 2 | miR-516b-4395172 | Undetermined |  |  |  |
| CTRL 3 | miR-516b-4395172 | Undetermined |  |  |  |
| ACM 1 | miR-517a-4395513 | Undetermined |  |  |  |
| ACM 2 | miR-517a-4395513 | Undetermined |  |  |  |
| ACM 3 | miR-517a-4395513 | Undetermined |  |  |  |
| CTRL 1 | miR-517a-4395513 | Undetermined |  |  |  |
| CTRL 2 | miR-517a-4395513 | Undetermined |  |  |  |
| CTRL 3 | miR-517a-4395513 | Undetermined |  |  |  |
| ACM 1 | miR-517b-4373244 | Undetermined |  |  |  |
| ACM 2 | miR-517b-4373244 | Undetermined |  |  |  |
| ACM 3 | miR-517b-4373244 | Undetermined |  |  |  |
| CTRL 1 | miR-517b-4373244 | Undetermined |  |  |  |
| CTRL 2 | miR-517b-4373244 | Undetermined |  |  |  |
| CTRL 3 | miR-517b-4373244 | Undetermined |  |  |  |
| ACM 1 | miR-517c-4373264 | Undetermined |  |  |  |
| ACM 2 | miR-517c-4373264 | Undetermined |  |  |  |
| ACM 3 | miR-517c-4373264 | Undetermined |  |  |  |
| CTRL 1 | miR-517c-4373264 | Undetermined |  |  |  |
| CTRL 2 | miR-517c-4373264 | Undetermined |  |  |  |
| CTRL 3 | miR-517c-4373264 | Undetermined |  |  |  |
| ACM 1 | miR-518a-3p-4395508 | Undetermined |  |  |  |
| ACM 2 | miR-518a-3p-4395508 | Undetermined |  |  |  |
| ACM 3 | miR-518a-3p-4395508 | Undetermined |  |  |  |
| CTRL 1 | miR-518a-3p-4395508 | Undetermined |  |  |  |
| CTRL 2 | miR-518a-3p-4395508 | Undetermined |  |  |  |
| CTRL 3 | miR-518a-3p-4395508 | Undetermined |  |  |  |
| ACM 1 | miR-518a-5p-4395507 | Undetermined |  |  |  |
| ACM 2 | miR-518a-5p-4395507 | Undetermined |  |  |  |
| ACM 3 | miR-518a-5p-4395507 | Undetermined |  |  |  |
| CTRL 1 | miR-518a-5p-4395507 | Undetermined |  |  |  |
| CTRL 2 | miR-518a-5p-4395507 | Undetermined |  |  |  |
| CTRL 3 | miR-518a-5p-4395507 | Undetermined |  |  |  |
| ACM 1 | miR-518b-4373246 | Undetermined |  |  |  |
| ACM 2 | miR-518b-4373246 | Undetermined |  |  |  |
| ACM 3 | miR-518b-4373246 | Undetermined |  |  |  |
| CTRL 1 | miR-518b-4373246 | Undetermined |  |  |  |
| CTRL 2 | miR-518b-4373246 | Undetermined |  |  |  |
| CTRL 3 | miR-518b-4373246 | Undetermined |  |  |  |
| ACM 1 | miR-518c-4395512 | Undetermined |  |  |  |
| ACM 2 | miR-518c-4395512 | Undetermined |  |  |  |
| ACM 3 | miR-518c-4395512 | Undetermined |  |  |  |
| CTRL 1 | miR-518c-4395512 | Undetermined |  |  |  |
| CTRL 2 | miR-518c-4395512 | Undetermined |  |  |  |
| CTRL 3 | miR-518c-4395512 | Undetermined |  |  |  |
| ACM 1 | miR-518d-3p-4373248 | Undetermined |  |  |  |
| ACM 2 | miR-518d-3p-4373248 | Undetermined |  |  |  |
| ACM 3 | miR-518d-3p-4373248 | Undetermined |  |  |  |
| CTRL 1 | miR-518d-3p-4373248 | Undetermined |  |  |  |
| CTRL 2 | miR-518d-3p-4373248 | Undetermined |  |  |  |
| CTRL 3 | miR-518d-3p-4373248 | Undetermined |  |  |  |
| ACM 1 | miR-518d-5p-4395500 | Undetermined |  |  |  |
| ACM 2 | miR-518d-5p-4395500 | Undetermined |  |  |  |
| ACM 3 | miR-518d-5p-4395500 | Undetermined |  |  |  |
| CTRL 1 | miR-518d-5p-4395500 | Undetermined |  |  |  |
| CTRL 2 | miR-518d-5p-4395500 | Undetermined |  |  |  |
| CTRL 3 | miR-518d-5p-4395500 | Undetermined |  |  |  |
| ACM 1 | miR-518e-4395506 | Undetermined |  |  |  |
| ACM 2 | miR-518e-4395506 | Undetermined |  |  |  |
| ACM 3 | miR-518e-4395506 | Undetermined |  |  |  |
| CTRL 1 | miR-518e-4395506 | Undetermined |  |  |  |
| CTRL 2 | miR-518e-4395506 | Undetermined |  |  |  |
| CTRL 3 | miR-518e-4395506 | Undetermined |  |  |  |
| ACM 1 | miR-518f-4395499 | Undetermined |  |  |  |
| ACM 2 | miR-518f-4395499 | Undetermined |  |  |  |
| ACM 3 | miR-518f-4395499 | Undetermined |  |  |  |
| CTRL 1 | miR-518f-4395499 | Undetermined |  |  |  |
| CTRL 2 | miR-518f-4395499 | Undetermined |  |  |  |
| CTRL 3 | miR-518f-4395499 | Undetermined |  |  |  |
| ACM 1 | miR-519a-4395526 | Undetermined |  |  |  |
| ACM 2 | miR-519a-4395526 | Undetermined |  |  |  |
| ACM 3 | miR-519a-4395526 | Undetermined |  |  |  |
| CTRL 1 | miR-519a-4395526 | Undetermined |  |  |  |
| CTRL 2 | miR-519a-4395526 | Undetermined |  |  |  |
| CTRL 3 | miR-519a-4395526 | Undetermined |  |  |  |
| ACM 1 | miR-519c-3p-4373251 | Undetermined |  |  |  |
| ACM 2 | miR-519c-3p-4373251 | Undetermined |  |  |  |
| ACM 3 | miR-519c-3p-4373251 | Undetermined |  |  |  |
| CTRL 1 | miR-519c-3p-4373251 | Undetermined |  |  |  |
| CTRL 2 | miR-519c-3p-4373251 | Undetermined |  |  |  |
| CTRL 3 | miR-519c-3p-4373251 | Undetermined |  |  |  |
| ACM 1 | miR-519d-4395514 | Undetermined |  |  |  |
| ACM 2 | miR-519d-4395514 | Undetermined |  |  |  |
| ACM 3 | miR-519d-4395514 | Undetermined |  |  |  |
| CTRL 1 | miR-519d-4395514 | Undetermined |  |  |  |
| CTRL 2 | miR-519d-4395514 | Undetermined |  |  |  |
| CTRL 3 | miR-519d-4395514 | Undetermined |  |  |  |
| ACM 1 | miR-519e-4395481 | Undetermined |  |  |  |
| ACM 2 | miR-519e-4395481 | Undetermined |  |  |  |
| ACM 3 | miR-519e-4395481 | Undetermined |  |  |  |
| CTRL 1 | miR-519e-4395481 | Undetermined |  |  |  |
| CTRL 2 | miR-519e-4395481 | Undetermined |  |  |  |
| CTRL 3 | miR-519e-4395481 | Undetermined |  |  |  |
| ACM 1 | miR-520a-3p-4373268 | Undetermined |  |  |  |
| ACM 2 | miR-520a-3p-4373268 | Undetermined |  |  |  |
| ACM 3 | miR-520a-3p-4373268 | Undetermined |  |  |  |
| CTRL 1 | miR-520a-3p-4373268 | Undetermined |  |  |  |
| CTRL 2 | miR-520a-3p-4373268 | Undetermined |  |  |  |
| CTRL 3 | miR-520a-3p-4373268 | Undetermined |  |  |  |
| ACM 1 | miR-520a-5p-4378085 | Undetermined |  |  |  |
| ACM 2 | miR-520a-5p-4378085 | Undetermined |  |  |  |
| ACM 3 | miR-520a-5p-4378085 | Undetermined |  |  |  |
| CTRL 1 | miR-520a-5p-4378085 | Undetermined |  |  |  |
| CTRL 2 | miR-520a-5p-4378085 | Undetermined |  |  |  |
| CTRL 3 | miR-520a-5p-4378085 | Undetermined |  |  |  |
| ACM 1 | miR-520b-4373252 | Undetermined |  |  |  |
| ACM 2 | miR-520b-4373252 | Undetermined |  |  |  |
| ACM 3 | miR-520b-4373252 | Undetermined |  |  |  |
| CTRL 1 | miR-520b-4373252 | Undetermined |  |  |  |
| CTRL 2 | miR-520b-4373252 | Undetermined |  |  |  |
| CTRL 3 | miR-520b-4373252 | Undetermined |  |  |  |
| ACM 1 | miR-520d-5p-4395504 | Undetermined |  |  |  |
| ACM 2 | miR-520d-5p-4395504 | Undetermined |  |  |  |
| ACM 3 | miR-520d-5p-4395504 | Undetermined |  |  |  |
| CTRL 1 | miR-520d-5p-4395504 | Undetermined |  |  |  |
| CTRL 2 | miR-520d-5p-4395504 | Undetermined |  |  |  |
| CTRL 3 | miR-520d-5p-4395504 | Undetermined |  |  |  |
| ACM 1 | miR-520e-4373255 | Undetermined |  |  |  |
| ACM 2 | miR-520e-4373255 | Undetermined |  |  |  |
| ACM 3 | miR-520e-4373255 | Undetermined |  |  |  |
| CTRL 1 | miR-520e-4373255 | Undetermined |  |  |  |
| CTRL 2 | miR-520e-4373255 | Undetermined |  |  |  |
| CTRL 3 | miR-520e-4373255 | Undetermined |  |  |  |
| ACM 1 | miR-520f-4373256 | Undetermined |  |  |  |
| ACM 2 | miR-520f-4373256 | Undetermined |  |  |  |
| ACM 3 | miR-520f-4373256 | Undetermined |  |  |  |
| CTRL 1 | miR-520f-4373256 | Undetermined |  |  |  |
| CTRL 2 | miR-520f-4373256 | Undetermined |  |  |  |
| CTRL 3 | miR-520f-4373256 | Undetermined |  |  |  |
| ACM 1 | miR-520g-4373257 | Undetermined |  |  |  |
| ACM 2 | miR-520g-4373257 | Undetermined |  |  |  |
| ACM 3 | miR-520g-4373257 | Undetermined |  |  |  |
| CTRL 1 | miR-520g-4373257 | Undetermined |  |  |  |
| CTRL 2 | miR-520g-4373257 | Undetermined |  |  |  |
| CTRL 3 | miR-520g-4373257 | Undetermined |  |  |  |
| ACM 1 | miR-521-4373259 | Undetermined |  |  |  |
| ACM 2 | miR-521-4373259 | Undetermined |  |  |  |
| ACM 3 | miR-521-4373259 | Undetermined |  |  |  |
| CTRL 1 | miR-521-4373259 | Undetermined |  |  |  |
| CTRL 2 | miR-521-4373259 | Undetermined |  |  |  |
| CTRL 3 | miR-521-4373259 | Undetermined |  |  |  |
| ACM 1 | miR-522-4395524 | Undetermined |  |  |  |
| ACM 2 | miR-522-4395524 | Undetermined |  |  |  |
| ACM 3 | miR-522-4395524 | Undetermined |  |  |  |
| CTRL 1 | miR-522-4395524 | Undetermined |  |  |  |
| CTRL 2 | miR-522-4395524 | Undetermined |  |  |  |
| CTRL 3 | miR-522-4395524 | Undetermined |  |  |  |
| ACM 1 | miR-523-4395497 | Undetermined |  |  |  |
| ACM 2 | miR-523-4395497 | Undetermined |  |  |  |
| ACM 3 | miR-523-4395497 | Undetermined |  |  |  |
| CTRL 1 | miR-523-4395497 | Undetermined |  |  |  |
| CTRL 2 | miR-523-4395497 | Undetermined |  |  |  |
| CTRL 3 | miR-523-4395497 | Undetermined |  |  |  |
| ACM 1 | miR-524-5p-4395174 | Undetermined |  |  |  |
| ACM 2 | miR-524-5p-4395174 | Undetermined |  |  |  |
| ACM 3 | miR-524-5p-4395174 | Undetermined |  |  |  |
| CTRL 1 | miR-524-5p-4395174 | Undetermined |  |  |  |
| CTRL 2 | miR-524-5p-4395174 | Undetermined |  |  |  |
| CTRL 3 | miR-524-5p-4395174 | Undetermined |  |  |  |
| ACM 1 | miR-525-3p-4395496 | Undetermined |  |  |  |
| ACM 2 | miR-525-3p-4395496 | Undetermined |  |  |  |
| ACM 3 | miR-525-3p-4395496 | Undetermined |  |  |  |
| CTRL 1 | miR-525-3p-4395496 | Undetermined |  |  |  |
| CTRL 2 | miR-525-3p-4395496 | Undetermined |  |  |  |
| CTRL 3 | miR-525-3p-4395496 | Undetermined |  |  |  |
| ACM 1 | miR-525-5p-4378088 | Undetermined |  |  |  |
| ACM 2 | miR-525-5p-4378088 | Undetermined |  |  |  |
| ACM 3 | miR-525-5p-4378088 | Undetermined |  |  |  |
| CTRL 1 | miR-525-5p-4378088 | Undetermined |  |  |  |
| CTRL 2 | miR-525-5p-4378088 | Undetermined |  |  |  |
| CTRL 3 | miR-525-5p-4378088 | Undetermined |  |  |  |
| ACM 1 | miR-526b-4395493 | Undetermined |  |  |  |
| ACM 2 | miR-526b-4395493 | Undetermined |  |  |  |
| ACM 3 | miR-526b-4395493 | Undetermined |  |  |  |
| CTRL 1 | miR-526b-4395493 | Undetermined |  |  |  |
| CTRL 2 | miR-526b-4395493 | Undetermined |  |  |  |
| CTRL 3 | miR-526b-4395493 | Undetermined |  |  |  |
| ACM 1 | miR-539-4378103 | Undetermined |  |  |  |
| ACM 2 | miR-539-4378103 | Undetermined |  |  |  |
| ACM 3 | miR-539-4378103 | Undetermined |  |  |  |
| CTRL 1 | miR-539-4378103 | Undetermined |  |  |  |
| CTRL 2 | miR-539-4378103 | Undetermined |  |  |  |
| CTRL 3 | miR-539-4378103 | Undetermined |  |  |  |
| ACM 1 | miR-541-4395312 | Undetermined |  |  |  |
| ACM 2 | miR-541-4395312 | Undetermined |  |  |  |
| ACM 3 | miR-541-4395312 | Undetermined |  |  |  |
| CTRL 1 | miR-541-4395312 | Undetermined |  |  |  |
| CTRL 2 | miR-541-4395312 | Undetermined |  |  |  |
| CTRL 3 | miR-541-4395312 | Undetermined |  |  |  |
| ACM 1 | miR-542-3p-4378101 | Undetermined |  |  |  |
| ACM 2 | miR-542-3p-4378101 | Undetermined |  |  |  |
| ACM 3 | miR-542-3p-4378101 | Undetermined |  |  |  |
| CTRL 1 | miR-542-3p-4378101 | Undetermined |  |  |  |
| CTRL 2 | miR-542-3p-4378101 | Undetermined |  |  |  |
| CTRL 3 | miR-542-3p-4378101 | Undetermined |  |  |  |
| ACM 1 | miR-542-5p-4395351 | Undetermined |  |  |  |
| ACM 2 | miR-542-5p-4395351 | Undetermined |  |  |  |
| ACM 3 | miR-542-5p-4395351 | Undetermined |  |  |  |
| CTRL 1 | miR-542-5p-4395351 | Undetermined |  |  |  |
| CTRL 2 | miR-542-5p-4395351 | Undetermined |  |  |  |
| CTRL 3 | miR-542-5p-4395351 | Undetermined |  |  |  |
| ACM 1 | miR-544-4395376 | Undetermined |  |  |  |
| ACM 2 | miR-544-4395376 | Undetermined |  |  |  |
| ACM 3 | miR-544-4395376 | Undetermined |  |  |  |
| CTRL 1 | miR-544-4395376 | Undetermined |  |  |  |
| CTRL 2 | miR-544-4395376 | Undetermined |  |  |  |
| CTRL 3 | miR-544-4395376 | Undetermined |  |  |  |
| ACM 1 | miR-545-4395378 | Undetermined |  |  |  |
| ACM 2 | miR-545-4395378 | Undetermined |  |  |  |
| ACM 3 | miR-545-4395378 | Undetermined |  |  |  |
| CTRL 1 | miR-545-4395378 | Undetermined |  |  |  |
| CTRL 2 | miR-545-4395378 | Undetermined |  |  |  |
| CTRL 3 | miR-545-4395378 | Undetermined |  |  |  |
| ACM 1 | miR-548a-3p-4380948 | Undetermined |  |  |  |
| ACM 2 | miR-548a-3p-4380948 | Undetermined |  |  |  |
| ACM 3 | miR-548a-3p-4380948 | Undetermined |  |  |  |
| CTRL 1 | miR-548a-3p-4380948 | Undetermined |  |  |  |
| CTRL 2 | miR-548a-3p-4380948 | Undetermined |  |  |  |
| CTRL 3 | miR-548a-3p-4380948 | Undetermined |  |  |  |
| ACM 1 | miR-548a-5p-4395523 | Undetermined |  |  |  |
| ACM 2 | miR-548a-5p-4395523 | Undetermined |  |  |  |
| ACM 3 | miR-548a-5p-4395523 | Undetermined |  |  |  |
| CTRL 1 | miR-548a-5p-4395523 | Undetermined |  |  |  |
| CTRL 2 | miR-548a-5p-4395523 | Undetermined |  |  |  |
| CTRL 3 | miR-548a-5p-4395523 | Undetermined |  |  |  |
| ACM 1 | miR-548b-3p-4380951 | Undetermined |  |  |  |
| ACM 2 | miR-548b-3p-4380951 | Undetermined |  |  |  |
| ACM 3 | miR-548b-3p-4380951 | Undetermined |  |  |  |
| CTRL 1 | miR-548b-3p-4380951 | Undetermined |  |  |  |
| CTRL 2 | miR-548b-3p-4380951 | Undetermined |  |  |  |
| CTRL 3 | miR-548b-3p-4380951 | Undetermined |  |  |  |
| ACM 1 | miR-548b-5p-4395519 | Undetermined |  |  |  |
| ACM 2 | miR-548b-5p-4395519 | Undetermined |  |  |  |
| ACM 3 | miR-548b-5p-4395519 | Undetermined |  |  |  |
| CTRL 1 | miR-548b-5p-4395519 | Undetermined |  |  |  |
| CTRL 2 | miR-548b-5p-4395519 | Undetermined |  |  |  |
| CTRL 3 | miR-548b-5p-4395519 | Undetermined |  |  |  |
| ACM 1 | miR-548c-3p-4380993 | Undetermined |  |  |  |
| ACM 2 | miR-548c-3p-4380993 | Undetermined |  |  |  |
| ACM 3 | miR-548c-3p-4380993 | Undetermined |  |  |  |
| CTRL 1 | miR-548c-3p-4380993 | Undetermined |  |  |  |
| CTRL 2 | miR-548c-3p-4380993 | Undetermined |  |  |  |
| CTRL 3 | miR-548c-3p-4380993 | Undetermined |  |  |  |
| ACM 1 | miR-548c-5p-4395540 | Undetermined |  |  |  |
| ACM 2 | miR-548c-5p-4395540 | Undetermined |  |  |  |
| ACM 3 | miR-548c-5p-4395540 | Undetermined |  |  |  |
| CTRL 1 | miR-548c-5p-4395540 | Undetermined |  |  |  |
| CTRL 2 | miR-548c-5p-4395540 | Undetermined |  |  |  |
| CTRL 3 | miR-548c-5p-4395540 | Undetermined |  |  |  |
| ACM 1 | miR-548d-3p-4381008 | Undetermined |  |  |  |
| ACM 2 | miR-548d-3p-4381008 | Undetermined |  |  |  |
| ACM 3 | miR-548d-3p-4381008 | Undetermined |  |  |  |
| CTRL 1 | miR-548d-3p-4381008 | Undetermined |  |  |  |
| CTRL 2 | miR-548d-3p-4381008 | Undetermined |  |  |  |
| CTRL 3 | miR-548d-3p-4381008 | Undetermined |  |  |  |
| ACM 1 | miR-548d-5p-4395348 | Undetermined |  |  |  |
| ACM 2 | miR-548d-5p-4395348 | Undetermined |  |  |  |
| ACM 3 | miR-548d-5p-4395348 | Undetermined |  |  |  |
| CTRL 1 | miR-548d-5p-4395348 | Undetermined |  |  |  |
| CTRL 2 | miR-548d-5p-4395348 | Undetermined |  |  |  |
| CTRL 3 | miR-548d-5p-4395348 | Undetermined |  |  |  |
| ACM 1 | miR-551b-4380945 | Undetermined |  |  |  |
| ACM 2 | miR-551b-4380945 | Undetermined |  |  |  |
| ACM 3 | miR-551b-4380945 | Undetermined |  |  |  |
| CTRL 1 | miR-551b-4380945 | Undetermined |  |  |  |
| CTRL 2 | miR-551b-4380945 | Undetermined |  |  |  |
| CTRL 3 | miR-551b-4380945 | Undetermined |  |  |  |
| ACM 1 | miR-556-3p-4395456 | Undetermined |  |  |  |
| ACM 2 | miR-556-3p-4395456 | Undetermined |  |  |  |
| ACM 3 | miR-556-3p-4395456 | Undetermined |  |  |  |
| CTRL 1 | miR-556-3p-4395456 | Undetermined |  |  |  |
| CTRL 2 | miR-556-3p-4395456 | Undetermined |  |  |  |
| CTRL 3 | miR-556-3p-4395456 | Undetermined |  |  |  |
| ACM 1 | miR-556-5p-4395455 | Undetermined |  |  |  |
| ACM 2 | miR-556-5p-4395455 | Undetermined |  |  |  |
| ACM 3 | miR-556-5p-4395455 | Undetermined |  |  |  |
| CTRL 1 | miR-556-5p-4395455 | Undetermined |  |  |  |
| CTRL 2 | miR-556-5p-4395455 | Undetermined |  |  |  |
| CTRL 3 | miR-556-5p-4395455 | Undetermined |  |  |  |
| ACM 1 | miR-561-4380938 | Undetermined |  |  |  |
| ACM 2 | miR-561-4380938 | Undetermined |  |  |  |
| ACM 3 | miR-561-4380938 | Undetermined |  |  |  |
| CTRL 1 | miR-561-4380938 | Undetermined |  |  |  |
| CTRL 2 | miR-561-4380938 | Undetermined |  |  |  |
| CTRL 3 | miR-561-4380938 | Undetermined |  |  |  |
| ACM 1 | miR-570-4395458 | Undetermined |  |  |  |
| ACM 2 | miR-570-4395458 | Undetermined |  |  |  |
| ACM 3 | miR-570-4395458 | Undetermined |  |  |  |
| CTRL 1 | miR-570-4395458 | Undetermined |  |  |  |
| CTRL 2 | miR-570-4395458 | Undetermined |  |  |  |
| CTRL 3 | miR-570-4395458 | Undetermined |  |  |  |
| ACM 1 | miR-576-5p-4395461 | Undetermined |  |  |  |
| ACM 2 | miR-576-5p-4395461 | Undetermined |  |  |  |
| ACM 3 | miR-576-5p-4395461 | Undetermined |  |  |  |
| CTRL 1 | miR-576-5p-4395461 | Undetermined |  |  |  |
| CTRL 2 | miR-576-5p-4395461 | Undetermined |  |  |  |
| CTRL 3 | miR-576-5p-4395461 | Undetermined |  |  |  |
| ACM 1 | miR-579-4395509 | Undetermined |  |  |  |
| ACM 2 | miR-579-4395509 | Undetermined |  |  |  |
| ACM 3 | miR-579-4395509 | Undetermined |  |  |  |
| CTRL 1 | miR-579-4395509 | Undetermined |  |  |  |
| CTRL 2 | miR-579-4395509 | Undetermined |  |  |  |
| CTRL 3 | miR-579-4395509 | Undetermined |  |  |  |
| ACM 1 | miR-582-3p-4395510 | Undetermined |  |  |  |
| ACM 2 | miR-582-3p-4395510 | Undetermined |  |  |  |
| ACM 3 | miR-582-3p-4395510 | Undetermined |  |  |  |
| CTRL 1 | miR-582-3p-4395510 | Undetermined |  |  |  |
| CTRL 2 | miR-582-3p-4395510 | Undetermined |  |  |  |
| CTRL 3 | miR-582-3p-4395510 | Undetermined |  |  |  |
| ACM 1 | miR-582-5p-4395175 | Undetermined |  |  |  |
| ACM 2 | miR-582-5p-4395175 | Undetermined |  |  |  |
| ACM 3 | miR-582-5p-4395175 | Undetermined |  |  |  |
| CTRL 1 | miR-582-5p-4395175 | Undetermined |  |  |  |
| CTRL 2 | miR-582-5p-4395175 | Undetermined |  |  |  |
| CTRL 3 | miR-582-5p-4395175 | Undetermined |  |  |  |
| ACM 1 | miR-589-4395520 | Undetermined |  |  |  |
| ACM 2 | miR-589-4395520 | Undetermined |  |  |  |
| ACM 3 | miR-589-4395520 | Undetermined |  |  |  |
| CTRL 1 | miR-589-4395520 | Undetermined |  |  |  |
| CTRL 2 | miR-589-4395520 | Undetermined |  |  |  |
| CTRL 3 | miR-589-4395520 | Undetermined |  |  |  |
| ACM 1 | miR-597-4380960 | Undetermined |  |  |  |
| ACM 2 | miR-597-4380960 | Undetermined |  |  |  |
| ACM 3 | miR-597-4380960 | Undetermined |  |  |  |
| CTRL 1 | miR-597-4380960 | Undetermined |  |  |  |
| CTRL 2 | miR-597-4380960 | Undetermined |  |  |  |
| CTRL 3 | miR-597-4380960 | Undetermined |  |  |  |
| ACM 1 | miR-598-4395179 | Undetermined |  |  |  |
| ACM 2 | miR-598-4395179 | Undetermined |  |  |  |
| ACM 3 | miR-598-4395179 | Undetermined |  |  |  |
| CTRL 1 | miR-598-4395179 | Undetermined |  |  |  |
| CTRL 2 | miR-598-4395179 | Undetermined |  |  |  |
| CTRL 3 | miR-598-4395179 | Undetermined |  |  |  |
| ACM 1 | miR-615-3p-4386777 | Undetermined |  |  |  |
| ACM 2 | miR-615-3p-4386777 | Undetermined |  |  |  |
| ACM 3 | miR-615-3p-4386777 | Undetermined |  |  |  |
| CTRL 1 | miR-615-3p-4386777 | Undetermined |  |  |  |
| CTRL 2 | miR-615-3p-4386777 | Undetermined |  |  |  |
| CTRL 3 | miR-615-3p-4386777 | Undetermined |  |  |  |
| ACM 1 | miR-615-5p-4395464 | Undetermined |  |  |  |
| ACM 2 | miR-615-5p-4395464 | Undetermined |  |  |  |
| ACM 3 | miR-615-5p-4395464 | Undetermined |  |  |  |
| CTRL 1 | miR-615-5p-4395464 | Undetermined |  |  |  |
| CTRL 2 | miR-615-5p-4395464 | Undetermined |  |  |  |
| CTRL 3 | miR-615-5p-4395464 | Undetermined |  |  |  |
| ACM 1 | miR-616-4395525 | Undetermined |  |  |  |
| ACM 2 | miR-616-4395525 | Undetermined |  |  |  |
| ACM 3 | miR-616-4395525 | Undetermined |  |  |  |
| CTRL 1 | miR-616-4395525 | Undetermined |  |  |  |
| CTRL 2 | miR-616-4395525 | Undetermined |  |  |  |
| CTRL 3 | miR-616-4395525 | Undetermined |  |  |  |
| ACM 1 | miR-618-4380996 | Undetermined |  |  |  |
| ACM 2 | miR-618-4380996 | Undetermined |  |  |  |
| ACM 3 | miR-618-4380996 | Undetermined |  |  |  |
| CTRL 1 | miR-618-4380996 | Undetermined |  |  |  |
| CTRL 2 | miR-618-4380996 | Undetermined |  |  |  |
| CTRL 3 | miR-618-4380996 | Undetermined |  |  |  |
| ACM 1 | miR-624-4395541 | Undetermined |  |  |  |
| ACM 2 | miR-624-4395541 | Undetermined |  |  |  |
| ACM 3 | miR-624-4395541 | Undetermined |  |  |  |
| CTRL 1 | miR-624-4395541 | Undetermined |  |  |  |
| CTRL 2 | miR-624-4395541 | Undetermined |  |  |  |
| CTRL 3 | miR-624-4395541 | Undetermined |  |  |  |
| ACM 1 | miR-625-4395542 | Undetermined |  |  |  |
| ACM 2 | miR-625-4395542 | Undetermined |  |  |  |
| ACM 3 | miR-625-4395542 | Undetermined |  |  |  |
| CTRL 1 | miR-625-4395542 | Undetermined |  |  |  |
| CTRL 2 | miR-625-4395542 | Undetermined |  |  |  |
| CTRL 3 | miR-625-4395542 | Undetermined |  |  |  |
| ACM 1 | miR-627-4380967 | Undetermined |  |  |  |
| ACM 2 | miR-627-4380967 | Undetermined |  |  |  |
| ACM 3 | miR-627-4380967 | Undetermined |  |  |  |
| CTRL 1 | miR-627-4380967 | Undetermined |  |  |  |
| CTRL 2 | miR-627-4380967 | Undetermined |  |  |  |
| CTRL 3 | miR-627-4380967 | Undetermined |  |  |  |
| ACM 1 | miR-628-5p-4395544 | Undetermined |  |  |  |
| ACM 2 | miR-628-5p-4395544 | Undetermined |  |  |  |
| ACM 3 | miR-628-5p-4395544 | Undetermined |  |  |  |
| CTRL 1 | miR-628-5p-4395544 | Undetermined |  |  |  |
| CTRL 2 | miR-628-5p-4395544 | Undetermined |  |  |  |
| CTRL 3 | miR-628-5p-4395544 | Undetermined |  |  |  |
| ACM 1 | miR-651-4381007 | Undetermined |  |  |  |
| ACM 2 | miR-651-4381007 | Undetermined |  |  |  |
| ACM 3 | miR-651-4381007 | Undetermined |  |  |  |
| CTRL 1 | miR-651-4381007 | Undetermined |  |  |  |
| CTRL 2 | miR-651-4381007 | Undetermined |  |  |  |
| CTRL 3 | miR-651-4381007 | Undetermined |  |  |  |
| ACM 1 | miR-653-4395403 | Undetermined |  |  |  |
| ACM 2 | miR-653-4395403 | Undetermined |  |  |  |
| ACM 3 | miR-653-4395403 | Undetermined |  |  |  |
| CTRL 1 | miR-653-4395403 | Undetermined |  |  |  |
| CTRL 2 | miR-653-4395403 | Undetermined |  |  |  |
| CTRL 3 | miR-653-4395403 | Undetermined |  |  |  |
| ACM 1 | miR-654-3p-4395350 | Undetermined |  |  |  |
| ACM 2 | miR-654-3p-4395350 | Undetermined |  |  |  |
| ACM 3 | miR-654-3p-4395350 | Undetermined |  |  |  |
| CTRL 1 | miR-654-3p-4395350 | Undetermined |  |  |  |
| CTRL 2 | miR-654-3p-4395350 | Undetermined |  |  |  |
| CTRL 3 | miR-654-3p-4395350 | Undetermined |  |  |  |
| ACM 1 | miR-654-5p-4381014 | Undetermined |  |  |  |
| ACM 2 | miR-654-5p-4381014 | Undetermined |  |  |  |
| ACM 3 | miR-654-5p-4381014 | Undetermined |  |  |  |
| CTRL 1 | miR-654-5p-4381014 | Undetermined |  |  |  |
| CTRL 2 | miR-654-5p-4381014 | Undetermined |  |  |  |
| CTRL 3 | miR-654-5p-4381014 | Undetermined |  |  |  |
| ACM 1 | miR-655-4381015 | Undetermined |  |  |  |
| ACM 2 | miR-655-4381015 | Undetermined |  |  |  |
| ACM 3 | miR-655-4381015 | Undetermined |  |  |  |
| CTRL 1 | miR-655-4381015 | Undetermined |  |  |  |
| CTRL 2 | miR-655-4381015 | Undetermined |  |  |  |
| CTRL 3 | miR-655-4381015 | Undetermined |  |  |  |
| ACM 1 | miR-671-3p-4395433 | Undetermined |  |  |  |
| ACM 2 | miR-671-3p-4395433 | Undetermined |  |  |  |
| ACM 3 | miR-671-3p-4395433 | Undetermined |  |  |  |
| CTRL 1 | miR-671-3p-4395433 | Undetermined |  |  |  |
| CTRL 2 | miR-671-3p-4395433 | Undetermined |  |  |  |
| CTRL 3 | miR-671-3p-4395433 | Undetermined |  |  |  |
| ACM 1 | miR-672-4395438 | Undetermined |  |  |  |
| ACM 2 | miR-672-4395438 | Undetermined |  |  |  |
| ACM 3 | miR-672-4395438 | Undetermined |  |  |  |
| CTRL 1 | miR-672-4395438 | Undetermined |  |  |  |
| CTRL 2 | miR-672-4395438 | Undetermined |  |  |  |
| CTRL 3 | miR-672-4395438 | Undetermined |  |  |  |
| ACM 1 | miR-674-4395193 | Undetermined |  |  |  |
| ACM 2 | miR-674-4395193 | Undetermined |  |  |  |
| ACM 3 | miR-674-4395193 | Undetermined |  |  |  |
| CTRL 1 | miR-674-4395193 | Undetermined |  |  |  |
| CTRL 2 | miR-674-4395193 | Undetermined |  |  |  |
| CTRL 3 | miR-674-4395193 | Undetermined |  |  |  |
| ACM 1 | miR-708-4395452 | Undetermined |  |  |  |
| ACM 2 | miR-708-4395452 | Undetermined |  |  |  |
| ACM 3 | miR-708-4395452 | Undetermined |  |  |  |
| CTRL 1 | miR-708-4395452 | Undetermined |  |  |  |
| CTRL 2 | miR-708-4395452 | Undetermined |  |  |  |
| CTRL 3 | miR-708-4395452 | Undetermined |  |  |  |
| ACM 1 | miR-758-4395180 | Undetermined |  |  |  |
| ACM 2 | miR-758-4395180 | Undetermined |  |  |  |
| ACM 3 | miR-758-4395180 | Undetermined |  |  |  |
| CTRL 1 | miR-758-4395180 | Undetermined |  |  |  |
| CTRL 2 | miR-758-4395180 | Undetermined |  |  |  |
| CTRL 3 | miR-758-4395180 | Undetermined |  |  |  |
| ACM 1 | miR-871-4395465 | Undetermined |  |  |  |
| ACM 2 | miR-871-4395465 | Undetermined |  |  |  |
| ACM 3 | miR-871-4395465 | Undetermined |  |  |  |
| CTRL 1 | miR-871-4395465 | Undetermined |  |  |  |
| CTRL 2 | miR-871-4395465 | Undetermined |  |  |  |
| CTRL 3 | miR-871-4395465 | Undetermined |  |  |  |
| ACM 1 | miR-872-4395375 | Undetermined |  |  |  |
| ACM 2 | miR-872-4395375 | Undetermined |  |  |  |
| ACM 3 | miR-872-4395375 | Undetermined |  |  |  |
| CTRL 1 | miR-872-4395375 | Undetermined |  |  |  |
| CTRL 2 | miR-872-4395375 | Undetermined |  |  |  |
| CTRL 3 | miR-872-4395375 | Undetermined |  |  |  |
| ACM 1 | miR-873-4395467 | Undetermined |  |  |  |
| ACM 2 | miR-873-4395467 | Undetermined |  |  |  |
| ACM 3 | miR-873-4395467 | Undetermined |  |  |  |
| CTRL 1 | miR-873-4395467 | Undetermined |  |  |  |
| CTRL 2 | miR-873-4395467 | Undetermined |  |  |  |
| CTRL 3 | miR-873-4395467 | Undetermined |  |  |  |
| ACM 1 | miR-875-3p-4395315 | Undetermined |  |  |  |
| ACM 2 | miR-875-3p-4395315 | Undetermined |  |  |  |
| ACM 3 | miR-875-3p-4395315 | Undetermined |  |  |  |
| CTRL 1 | miR-875-3p-4395315 | Undetermined |  |  |  |
| CTRL 2 | miR-875-3p-4395315 | Undetermined |  |  |  |
| CTRL 3 | miR-875-3p-4395315 | Undetermined |  |  |  |
| ACM 1 | miR-876-3p-4395336 | Undetermined |  |  |  |
| ACM 2 | miR-876-3p-4395336 | Undetermined |  |  |  |
| ACM 3 | miR-876-3p-4395336 | Undetermined |  |  |  |
| CTRL 1 | miR-876-3p-4395336 | Undetermined |  |  |  |
| CTRL 2 | miR-876-3p-4395336 | Undetermined |  |  |  |
| CTRL 3 | miR-876-3p-4395336 | Undetermined |  |  |  |
| ACM 1 | miR-876-5p-4395316 | Undetermined |  |  |  |
| ACM 2 | miR-876-5p-4395316 | Undetermined |  |  |  |
| ACM 3 | miR-876-5p-4395316 | Undetermined |  |  |  |
| CTRL 1 | miR-876-5p-4395316 | Undetermined |  |  |  |
| CTRL 2 | miR-876-5p-4395316 | Undetermined |  |  |  |
| CTRL 3 | miR-876-5p-4395316 | Undetermined |  |  |  |
| ACM 1 | miR-885-3p-4395483 | Undetermined |  |  |  |
| ACM 2 | miR-885-3p-4395483 | Undetermined |  |  |  |
| ACM 3 | miR-885-3p-4395483 | Undetermined |  |  |  |
| CTRL 1 | miR-885-3p-4395483 | Undetermined |  |  |  |
| CTRL 2 | miR-885-3p-4395483 | Undetermined |  |  |  |
| CTRL 3 | miR-885-3p-4395483 | Undetermined |  |  |  |
| ACM 1 | miR-887-4395485 | Undetermined |  |  |  |
| ACM 2 | miR-887-4395485 | Undetermined |  |  |  |
| ACM 3 | miR-887-4395485 | Undetermined |  |  |  |
| CTRL 1 | miR-887-4395485 | Undetermined |  |  |  |
| CTRL 2 | miR-887-4395485 | Undetermined |  |  |  |
| CTRL 3 | miR-887-4395485 | Undetermined |  |  |  |
| ACM 1 | miR-888-4395323 | Undetermined |  |  |  |
| ACM 2 | miR-888-4395323 | Undetermined |  |  |  |
| ACM 3 | miR-888-4395323 | Undetermined |  |  |  |
| CTRL 1 | miR-888-4395323 | Undetermined |  |  |  |
| CTRL 2 | miR-888-4395323 | Undetermined |  |  |  |
| CTRL 3 | miR-888-4395323 | Undetermined |  |  |  |
| ACM 1 | miR-889-4395313 | Undetermined |  |  |  |
| ACM 2 | miR-889-4395313 | Undetermined |  |  |  |
| ACM 3 | miR-889-4395313 | Undetermined |  |  |  |
| CTRL 1 | miR-889-4395313 | Undetermined |  |  |  |
| CTRL 2 | miR-889-4395313 | Undetermined |  |  |  |
| CTRL 3 | miR-889-4395313 | Undetermined |  |  |  |
| ACM 1 | miR-890-4395320 | Undetermined |  |  |  |
| ACM 2 | miR-890-4395320 | Undetermined |  |  |  |
| ACM 3 | miR-890-4395320 | Undetermined |  |  |  |
| CTRL 1 | miR-890-4395320 | Undetermined |  |  |  |
| CTRL 2 | miR-890-4395320 | Undetermined |  |  |  |
| CTRL 3 | miR-890-4395320 | Undetermined |  |  |  |
| ACM 1 | miR-891a-4395302 | Undetermined |  |  |  |
| ACM 2 | miR-891a-4395302 | Undetermined |  |  |  |
| ACM 3 | miR-891a-4395302 | Undetermined |  |  |  |
| CTRL 1 | miR-891a-4395302 | Undetermined |  |  |  |
| CTRL 2 | miR-891a-4395302 | Undetermined |  |  |  |
| CTRL 3 | miR-891a-4395302 | Undetermined |  |  |  |
| ACM 1 | miR-891b-4395321 | Undetermined |  |  |  |
| ACM 2 | miR-891b-4395321 | Undetermined |  |  |  |
| ACM 3 | miR-891b-4395321 | Undetermined |  |  |  |
| CTRL 1 | miR-891b-4395321 | Undetermined |  |  |  |
| CTRL 2 | miR-891b-4395321 | Undetermined |  |  |  |
| CTRL 3 | miR-891b-4395321 | Undetermined |  |  |  |
| ACM 1 | miR-892a-4395306 | Undetermined |  |  |  |
| ACM 2 | miR-892a-4395306 | Undetermined |  |  |  |
| ACM 3 | miR-892a-4395306 | Undetermined |  |  |  |
| CTRL 1 | miR-892a-4395306 | Undetermined |  |  |  |
| CTRL 2 | miR-892a-4395306 | Undetermined |  |  |  |
| CTRL 3 | miR-892a-4395306 | Undetermined |  |  |  |
| ACM 1 | miR-9-4373285 | Undetermined |  |  |  |
| ACM 2 | miR-9-4373285 | Undetermined |  |  |  |
| ACM 3 | miR-9-4373285 | Undetermined |  |  |  |
| CTRL 1 | miR-9-4373285 | Undetermined |  |  |  |
| CTRL 2 | miR-9-4373285 | Undetermined |  |  |  |
| CTRL 3 | miR-9-4373285 | Undetermined |  |  |  |
| ACM 1 | miR-96-4373372 | Undetermined |  |  |  |
| ACM 2 | miR-96-4373372 | Undetermined |  |  |  |
| ACM 3 | miR-96-4373372 | Undetermined |  |  |  |
| CTRL 1 | miR-96-4373372 | Undetermined |  |  |  |
| CTRL 2 | miR-96-4373372 | Undetermined |  |  |  |
| CTRL 3 | miR-96-4373372 | Undetermined |  |  |  |
| ACM 1 | miR-98-4373009 | Undetermined |  |  |  |
| ACM 2 | miR-98-4373009 | Undetermined |  |  |  |
| ACM 3 | miR-98-4373009 | Undetermined |  |  |  |
| CTRL 1 | miR-98-4373009 | Undetermined |  |  |  |
| CTRL 2 | miR-98-4373009 | Undetermined |  |  |  |
| CTRL 3 | miR-98-4373009 | Undetermined |  |  |  |
| ACM 1 | miR-99a-4373008 | Undetermined |  |  |  |
| ACM 2 | miR-99a-4373008 | Undetermined |  |  |  |
| ACM 3 | miR-99a-4373008 | Undetermined |  |  |  |
| CTRL 1 | miR-99a-4373008 | Undetermined |  |  |  |
| CTRL 2 | miR-99a-4373008 | Undetermined |  |  |  |
| CTRL 3 | miR-99a-4373008 | Undetermined |  |  |  |
| ACM 1 | MammU6-4395470 | Undetermined |  |  |  |
| ACM 1 | MammU6-4395470 | Undetermined |  |  |  |
| ACM 1 | MammU6-4395470 | Undetermined |  |  |  |
| ACM 1 | MammU6-4395470 | Undetermined |  |  |  |
| ACM 2 | MammU6-4395470 | Undetermined |  |  |  |
| ACM 2 | MammU6-4395470 | Undetermined |  |  |  |
| ACM 2 | MammU6-4395470 | Undetermined |  |  |  |
| ACM 2 | MammU6-4395470 | Undetermined |  |  |  |
| ACM 3 | MammU6-4395470 | Undetermined |  |  |  |
| ACM 3 | MammU6-4395470 | Undetermined |  |  |  |
| ACM 3 | MammU6-4395470 | Undetermined |  |  |  |
| ACM 3 | MammU6-4395470 | Undetermined |  |  |  |
| CTRL 1 | MammU6-4395470 | Undetermined |  |  |  |
| CTRL 1 | MammU6-4395470 | Undetermined |  |  |  |
| CTRL 1 | MammU6-4395470 | Undetermined |  |  |  |
| CTRL 1 | MammU6-4395470 | Undetermined |  |  |  |
| CTRL 2 | MammU6-4395470 | Undetermined |  |  |  |
| CTRL 2 | MammU6-4395470 | Undetermined |  |  |  |
| CTRL 2 | MammU6-4395470 | Undetermined |  |  |  |
| CTRL 2 | MammU6-4395470 | Undetermined |  |  |  |
| CTRL 3 | MammU6-4395470 | Undetermined |  |  |  |
| CTRL 3 | MammU6-4395470 | Undetermined |  |  |  |
| CTRL 3 | MammU6-4395470 | Undetermined |  |  |  |
| CTRL 3 | MammU6-4395470 | Undetermined |  |  |  |
| ACM 1 | RNU44-4373384 | Undetermined |  |  |  |
| ACM 2 | RNU44-4373384 | Undetermined |  |  |  |
| ACM 3 | RNU44-4373384 | Undetermined |  |  |  |
| CTRL 1 | RNU44-4373384 | Undetermined |  |  |  |
| CTRL 2 | RNU44-4373384 | Undetermined |  |  |  |
| CTRL 3 | RNU44-4373384 | Undetermined |  |  |  |
| ACM 1 | RNU48-4373383 | Undetermined |  |  |  |
| ACM 2 | RNU48-4373383 | Undetermined |  |  |  |
| ACM 3 | RNU48-4373383 | Undetermined |  |  |  |
| CTRL 1 | RNU48-4373383 | Undetermined |  |  |  |
| CTRL 2 | RNU48-4373383 | Undetermined |  |  |  |
| CTRL 3 | RNU48-4373383 | Undetermined |  |  |  |
